# Ethnoscientific expertise and knowledge specialisation in 55 traditional cultures

**DOI:** 10.1017/ehs.2021.31

**Published:** 2021-06-14

**Authors:** Aaron D. Lightner, Cynthiann Heckelsmiller, Edward H. Hagen

**Affiliations:** Department of Anthropology, Washington State University, Pullman, WA, USA

**Keywords:** Ethnoscience, expertise, cultural transmission, conceptual knowledge, eHRAF

## Abstract

People everywhere acquire high levels of conceptual knowledge about their social and natural worlds, which we refer to as *ethnoscientific expertise*. Evolutionary explanations for expertise are still widely debated. We analysed ethnographic text records (*N* = 547) describing ethnoscientific expertise among 55 cultures in the Human Relations Area Files to investigate the mutually compatible roles of collaboration, proprietary knowledge, cultural transmission, honest signalling, and mate provisioning. We found relatively high levels of evidence for collaboration, proprietary knowledge, and cultural transmission, and lower levels of evidence for honest signalling and mate provisioning. In our exploratory analyses, we found that whether expertise involved proprietary vs. transmitted knowledge depended on the domain of expertise. Specifically, medicinal knowledge was positively associated with secretive and specialised knowledge for resolving uncommon and serious problems, i.e. proprietary knowledge. Motor skill-related expertise, such as subsistence and technological skills, was positively associated with broadly competent and generous teachers, i.e. cultural transmission. We also found that collaborative expertise was central to both of these models, and was generally important across different knowledge and skill domains.

## Graphical abstract

**Figure:**
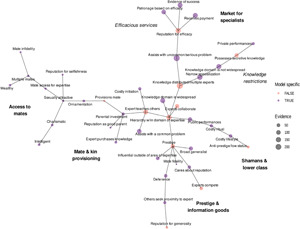

**Social media summary:** In a cross-cultural study we found that experts with observable motor skills like toolmaking were often teachers, but specialists with conceptual know-how for uncommon problems like illness used secretive knowledge to help clients.

## 1. Introduction

Humans are intuitive scientists (Kuhn, 1989; Szollosi & Newell, 2020). People everywhere acquire knowledge about fitness-relevant properties of their social and natural worlds (Albuquerque, Medeiros, & Casas, 2015; Atran, 1993; Gopnik, Meltzoff, & Kuhl, 2000), sort novel stimuli into classification systems (Ellen, 2006; Lakoff, 1987; Ortony & Medin, 1989) and infer patterns and causation from noisy phenomena (Cosmides & Tooby, 1996; Gigerenzer & Murray, 2015; Sperber, Premack, & Premack, 1995). Individual knowledge becomes cultural knowledge via social learning (Henrich et al., 2001; Henrich & McElreath, 2003; Richerson & Boyd, 2005), and discourse and argumentation (Mercier & Sperber, 2017).

Existing research has focused on the cognitive, social and ecological factors influencing the formation and dissemination of *ethnoscientific knowledge*, defined as culturally varying and locally useful bodies of conceptual knowledge about the social and natural world (Atran & Medin, 2008; see also Heintz, 2007). It is less clear, however, how and why some individuals might pursue relatively high levels of domain-specific conceptual knowledge compared with others within their population, which we will refer to as *ethnoscientific expertise*. (‘Ethnoscience’ can also refer to a particular Western scientific approach to ethnographic research on indigenous knowledge systems (Sturtevant, 1964), which today is usually referred to as cognitive anthropology (Kronenfeld, 2011). This is in contrast to our usage, which refers to the *content* of these indigenous knowledge systems, which are often culturally specific.)

### 1.1. The conundrum of ethnoscientific expertise

Levels of knowledge and skill vary for almost any ability. Expertise refers to domain-specific knowledge or skills reliably performed in a way that is superior to that of most other people (Ericsson & Charness, 1994). (For our purposes, expertise will be defined relative to other people in an individual's local community.) Explanations of how and why experts emerge with extensive knowledge often focus on proximate-level descriptions (Tinbergen, 1963). In the mainstream psychology literature these explanations typically focus on natural ability, favourable circumstances during development, and/or dedicated and systematic practice (Ericsson & Charness, 1994). In the genetic-developmental literature, these explanations focus on the closely related cognitive, genetic and developmental aspects of expertise (Dukas, 2019; Plomin, Shakeshaft, McMillan, & Trzaskowski, 2014). Some consensus exists among scholars about the necessity-but-insufficiency of each of these predisposing factors. Ultimate-level functional and strategic explanations for investing in expertise vs. other uses of one's time and energy, however, are lacking.

Multiple open questions therefore remain about the evolution of expertise. First, acquiring expertise in one domain, such as botany, zoology, physiology or meteorology, incurs an opportunity cost owing to time and energetic constraints, e.g. by reducing knowledge of other domains and reducing investment in other fitness-increasing activities. For example, an adaptive learning strategy could be to acquire practical knowledge about the world during early stages of development, and to divert this investment towards other efforts upon reaching adulthood, such as reproduction and subsistence (i.e. optimising an explore–exploit tradeoff; see Gopnik, 2020).

Second, environments are typically structured in ways that favour simple heuristics (Gigerenzer & Todd, 2001; Sloman & Fernbach, 2017). A forager, for example, could discern edible vs. poisonous berries with simple, locally relevant rules (e.g. discriminating based on colour, taste or location) that make a complex botanical theory unnecessary for survival. Simple rules can be useful for a range of computationally complex tasks, such as predicting seasonal weather patterns and animal behaviours, or navigating social relationships (Gigerenzer, Hertwig, & Pachur, 2011). Nevertheless, complex and elaborate theories about these tasks, which might or might not be useful on a day-to-day basis, are well documented in a variety of ethnoscientific domains across cultures (Albuquerque, Medeiros, & Casas, 2015). Moreover, across a population, individual knowledge about these elaborate theories often varies (Kronenfeld, 2011) and might only be mastered by relatively few individuals in the population, i.e. experts (Berlin & Berlin, 2015; Reyes-García et al., 2009).

Finally, it is not clear how expertise relates to cultural transmission. Cultural evolutionary theorists often highlight trait variation and imitation of skills that involve easily observable and transmissible behaviours, such as boat making or food preparation (Boyd, Richerson, & Henrich, 2011; Henrich, 2016). The costs and benefits of adopting easily observable traits, however, can be difficult to evaluate. It can therefore be adaptive to adopt a ‘package’ of transmissible traits that are either common in the population or are exhibited by ‘successful’ individuals (Henrich, 2016; Richerson & Boyd, 2005).

It is less obvious that *unobservable* and mostly *conceptual* knowledge, e.g. about plants, animals or weather patterns, can be grouped with easily observable behaviours in a single overarching category of transmissible cultural traits (Morin, 2015; Read & Andersson, 2019). Assumptions about expertise, such as the scope for improving the competence of most individuals in the population on a task, might need to vary according to whether the skills and knowledge are easy to observe, such as motor skills, or more difficult to observe, such as conceptual knowledge, and how often the knowledge and skills are useful (Ericsson & Charness, 1994).

Further, if expertise requires an individual learning cost (e.g. time spent practising, innovating or experimenting to improve a skill), and socially learning from an expert is possible, then evolutionary models show that without additional benefits to the expert, populations with social learning gain no fitness advantages relative to those without it (Rogers, 1988). This suggests that expertise requires a fitness advantage to offset the costs of individual learning (Boyd & Richerson, 1995).

In short, why do some individuals invest more heavily in ethnoscientific knowledge than others? If a given knowledge domain is not obviously practical or useful on a day-to-day basis, how do experts apply their knowledge, if at all, to increase their inclusive fitness?

### 1.2. Study aims: investigating evolutionary theories of expertise in the ethnographic record

In this study, which is largely exploratory, we consider multiple theoretical perspectives on knowledge and skill acquisition that might explain expertise as an evolutionary strategy, examine them in the ethnographic record, and then consider if our results warrant a new theory that is specific to expertise. Many previous cultural evolutionary theories have modelled skill acquisition and transmission in practical knowledge domains. These theories emphasise *imitation*, i.e. copying observable behaviours involving substantial motor activity, such as boat making (Boyd et al., 2011) and food preparation (Henrich, 2016).

We diverge from prior studies by emphasising *conceptual* knowledge, a consequence of our focus on ethnoscientific expertise. Ethnoscience in this study includes elaborate systems of knowledge, such as botany or medicine, where concepts are interrelated via rules and principles ‘concerning phenomena of the external world and of the human organism’ (Human Relations Area Files, 2021). These social or natural principles might (or might not) be used for practical applications with observable motor activity, such as bone-setting, weaving or hunting. It is therefore inevitable that practical activities will emerge from our systematic search of ethnography. Our restricted search criteria, however, ensures that practical applications are secondary to the ethnoscientific knowledge on which we focus (discussed further in the Methods section).

We also make an important distinction between *products* of knowledge vs. *know-how*. *Products* refer to observable applications of knowledge, whereas *know-how* refers to the underlying cognitive system or process – sometimes easily inferred from behaviour and sometimes not – that reliably yields a desired product. Importantly, *products do not necessarily reveal know-how*. For example, a doctor might know how to diagnose and treat illness. The patient, however, cannot gain the know-how used for her diagnosis and treatment by imitating the doctor. That is, if a skill requires complex conceptual knowledge, then observation is often insufficient to acquire that skill (Morin, 2015).

In order to systematically code ethnographic texts for variables that might explain how and why individuals would invest heavily in know-how (conceptual knowledge), we identify influential evolutionary theories of knowledge and skill acquisition. It is important to note that these theories were not necessarily formulated to explain the acquisition of ethnoscientific expertise or conceptual knowledge. We therefore take them as our starting point, operationalising each into five sets of overlapping variables, which we term our ‘theoretical models’ (see the Supporting Information for extended supporting quotations and further discussion that support our operationalisations).

We evaluate the degree to which each theoretical model is supported by the ethnographic evidence on expertise. These theoretical models are best understood as theoretical *perspectives* based on ‘family resemblances’, however, rather than as formal hypotheses. Our overarching aim is therefore not to simply support one model over the others but instead to start from existing ideas, assess the circumstances under which each model applies and use exploratory methods to move towards a data-driven theory of conceptual expertise.

#### 1.2.1. Cultural transmission model

Dual inheritance theorists characterise the human brain as a device for learning, storing and transmitting cultural information (Muthukrishna, Doebeli, Chudek, & Henrich, 2018; Richerson & Boyd, 2005), and argue that social learning is strictly necessary to explain human evolutionary success (Boyd et al., 2011; Henrich, 2016). In the cultural transmission model, experts commit high levels of cultural knowledge to memory, and are a source of socially transmitted knowledge to others in the population (Boyd & Richerson, 1985), generally in exchange for a fitness benefit (Boyd et al., 2011; Henrich & Gil-White, 2001). Fitness benefits conferred to experts might include material resources, cooperative partnerships or services from an apprentice or peer, all of which may contribute to reproductive success (Jiménez & Mesoudi, 2019; Price & Van Vugt, 2015).

The *cultural transmission model* also explains *how* skillful persons are identified: prestige is a cue of competence that allows others to selectively learn from, and direct freely conferred deference to, experts (Henrich & Gil-White, 2001; Henrich & McElreath, 2003). Prestige might include culturally specific concepts, symbols or other conspicuous indications of success (Boyd & Richerson, 1985; Henrich et al., 2001). For the *cultural transmission model*, experts do not have proprietary or secretive knowledge that they withhold from laypersons, but instead transmit their valuable knowledge based on proximity sought by acolytes, who somehow benefit experts in return. Importantly, valuable knowledge for the *cultural transmission model* should not only include products such as advice or assistance, but also know-how that the expert possesses, such as plant knowledge or technological skills that acolytes can use in the future.

*Cultural transmission model* predictions include widely distributed knowledge addressing common, day-to-day problems (e.g. subsistence-related activities, technological skills); experts with reputations for efficacious solutions to those problems; and experts who share know-how with other experts or non-experts, often in the context of mentorship or apprenticeship. Additional predictions include prestigious and high-status experts, deference to experts, experts who have a reputation for generosity and/or are preferred social partners beyond their domains of expertise, and experts with influence beyond their domains of expertise (e.g. medicinal experts who also have political authority).

#### 1.2.2. Proprietary knowledge model

Many services provided by experts, such as predictions, advice or medical care, require underlying know-how that is not readily transmissible without effortful teaching and practice (Ericsson & Charness, 1994; Hagen & Garfield, 2019; Morin, 2015). In contrast to the *cultural transmission model*, which emphasises an adaptive capacity for culture and its attendant wide distribution of knowledge (e.g. Boyd et al., 2011), we formulated the proprietary knowledge model, which proposes that experts’ conceptual knowledge is not readily transmissible, but restricted to specialists. A central idea for the *proprietary knowledge model* is that experts can use know-how to provide a valuable service or product to other people, who themselves do not possess solutions of their own, and who cannot subsequently transmit this learned information to others. A cost-effective strategy for addressing rare and consequential problems might be to consult a specialised expert in exchange for a complementary service or payment. According to the *proprietary knowledge model*, the value of an expert is determined by his or her relatively rare ability to provide a specific efficacious service (Tooby & Cosmides, 1996).

The commodities in a *biological market* of mutually beneficial partnerships (Hammerstein and Noë, 2016) can include providing information (Bouhlel, Wu, Ilanaki, & Goldstone, 2018) in exchange for similarly valuable benefits (e.g. payments and continuing patronage to the expert). An expert's high value in this market requires that she is hard to replace (Tooby & Cosmides, 1996). Hence, according to the *proprietary knowledge model*, services (*products*) are readily shared, but the rare *know-how* used to generate shared outcomes is proprietary, difficult to reverse engineer and difficult for non-experts to apply and achieve similar outcomes (Hagen & Garfield, 2019).

*Proprietary knowledge model* predictions include: experts' services successfully conferring some kind of benefit; experts having reputations for efficacy and gaining patronage based on efficacy; narrow specialisation in a domain-specific problem that is uncommon and serious when it arises; rare, secretive know-how employed in an opaque (i.e. not easily observable) process by which products are provided; and material resources received in return as payment.

#### 1.2.3. Collaborative cognition model

The *collaborative cognition model* emphasises that knowledge and expertise are highly social. Activities among multiple specialist types are collaborative on this view, and each expert has complementary roles, insights and areas of specialisation. Contrary to popular images of science, creativity and innovations, the *collaborative cognition model* proposes that concepts are not improved by lone geniuses or individual experts, and rarely if ever emerge as fully formed ideas (Mercier & Sperber, 2017; Sloman & Fernbach, 2017). Instead, cognitive tasks are often distributed across multiple interdependent roles, allowing experts to invest heavily in some areas of expertise while relying on other experts for information in other areas, a division of cognitive labour (Heintz, 2004; Hutchins, 2000; Keil, 2003). According to the *collaborative cognition model*, a high level of interaction between cognitive, sociocultural and ecological factors collectively shapes concepts, theories, solutions to domain-specific problems and even the questions that experts consider in the first place (Nersessian, 2010; Sperber, 1996).

*Collaborative cognition model* predictions include distributed expertise across multiple types of narrow specialist (i.e. a division of labour), collaboration among specialists that collectively produces more knowledge than each individual possesses, and knowledge (know-how) that is shared or exchanged among multiple experts.

#### 1.2.4. Honest signalling model

Spence (1978) argued that candidates in a job market can honestly signal their general competence, which is otherwise opaque to employers, with credentials that are relatively less costly to acquire for those with greater general competence. In evolutionary biology, a similar argument suggests that costly traits reliably signal genetic quality in a mating market (Grafen, 1990; Penn & Számadó, 2020; Zahavi, 1975). Sexual selection based on costly signals of fitness is hypothesised to explain a number of human traits, such as male hunting behaviour and conspicuous meat sharing to gain mating opportunities (Smith & Bird, 2000). Abilities must not only be successfully broadcast, but typically gain traction in a given cultural milieu in the form of social standing, locally relevant indicators of success and prestige (Hawkes & Bliege Bird, 2002; Winegard, Winegard, & Geary, 2018).

Applied to expertise, the honest signalling model proposes that displays of knowledge serve as a costly signal of genetic quality to prospective mates (i.e. that expertise is less costly to obtain for those with higher genetic quality). On this view, culture largely consists of conspicuous ‘courtship adaptations’ (Geher & Miller, 2007; Miller, 2011; Miller, 1999: 81), and creativity and intelligence are relevant underlying traits that are signalled by displays of expertise. This might not only include displays of erudition, however, but also proximate indicators of expert status, such as ornamentation, among other indications of prestige (Cheng & Tracy, 2014).

Predictions based on the *honest signalling model* prioritise overt displays of knowledgeability and skill, status and prestige; a short-term mating market in which signals are produced; and experts’ access to multiple mates. The *honest signalling model* refers specifically to signalling genetic quality to potential mates, and is not meant to represent a comprehensive model of the role of signalling in all forms of status competition. Hence, fitness indicators, such as culturally relevant displays of expertise, should be difficult for those with low genetic quality to achieve. Because our data did not have measures of genetic quality, we looked for evidence that acquisition of expertise involved clear costs.

#### 1.2.5. Mate provisioning model

Human social hierarchies and mate competition involve not only physical formidability, as seen in gorillas and chimpanzees, but also ‘prestige’ – culturally defined symbols of success that often involve valued skills and knowledge (Barkow, 1989; Maner & Case, 2016; Van Vugt & Smith, 2019). Humans also diverged from chimpanzees and gorillas in their shift towards strong male–female pair-bonding and increased paternal investment in offspring (Alger, Hooper, Cox, Stieglitz, & Kaplan, 2020; Kaplan, Hooper, & Gurven, 2009), sexual selection for which would have included individuals choosing mates based on their relatively high levels of resource access and provisioning behaviour (Buss, 1989; Gavrilets, 2012).

In contrast to the short-term mating strategy outlined in the *honest signalling model*, the *mate provisioning model* suggests that expertise is a means of competing for mates by increasing one's ability to provision their offspring either directly or by controlling resource production (Barkow, Cosmides, & Tooby, 1992; Stewart-Williams & Thomas, 2013). That is, mates choose prestigious and high-skilled experts based on their prospects for long-term parental investment and mate provisioning (Barkow, 1989; Barkow et al., 1992; Schmitt, 2008). Researchers have suggested similar hypotheses about sexually selected hunting behaviours among males who preferentially provision food to their kin (Buss, 1995; Hill & Hurtado, 1989).

Predictions based on the *mate provisioning model* include skill and knowledge acquisition involved in expertise that is best understood in terms of its practical applications, which are preferentially used to acquire resources for mates and offspring (Kaplan, Hill, Lancaster, & Hurtado, 2000). Hence, predictions based on the *mate provisioning model* include status and prestige, mate choice based on male provisioning prospects (e.g. reputations for generosity, commitment to offspring), actual evidence of investment towards offspring and high levels of mate fidelity.

### 1.3. Similarities and differences among theoretical models

Although many of the predictions described here are specific to one theoretical model, some are compatible with multiple models. We refer to these predictions as model-*specific* and model-*generic*, respectively. Four of the five models (*cultural transmission model*, *proprietary knowledge model*, *mate provisioning model*, *honest signalling model*) are premised on a hierarchy among skill levels, and prestige is central to at least three of these (*cultural transmission model*, *mate provisioning model*, *honest signalling model*). The *cultural transmission model*, *proprietary knowledge model*, *mate provisioning model* and *honest signalling model* emphasise fitness benefits to experts, but these models largely differ on how and why expertise is beneficial. This is especially clear, for example, in the mate access conferred for prestige described in sexual selection models (*honest signalling model* and *mate provisioning model*) vs. the deference and resource access in *cultural transmission model*. Resource access is common to the *mate provisioning model*, *cultural transmission model* and *proprietary knowledge model*, but the latter two make no predictions about provisioning of those resources to mates.

The *proprietary knowledge model* sharply diverges from the *cultural transmission model* by focusing on shared products and secretive know-how, which might be conditionally shared for a direct benefit. The *proprietary knowledge model*, which emphasises *barriers* to knowledge transmission, would nevertheless require some transmission of knowledge systems from experts to novices, meaning that it is at least partially compatible with the *cultural transmission model*.

The *collaborative cognition model* is uniquely compatible with other models. Rather than providing a strictly evolutionary explanation for expertise, it emphasises the distributed and collaborative social structure that might underlie expertise at a group level, in addition to the competition inherent to the other four models.

## 2. Methods

To characterise ethnoscientific expertise and assess the level of cross-cultural support for each theoretical model, we used data from the electronic Human Relations Area Files (eHRAF). The eHRAF is a digitised database of over a million pages of primary ethnographic documents, spanning several centuries, from over 400 cultures around the world. We restricted our search to the Probability Sample Files, a stratified subset of 60 cultures in the eHRAF that includes one randomly selected culture from 60 geographically diverse areas (Naroll, 1967). All documents in the eHRAF are coded at the paragraph level using an Outline of Cultural Materials (OCM), a hierarchically organised coding scheme containing several hundred numeric codes that are assigned to a unique and specific topic (Murdock et al., 2006). Paragraphs usually relate to multiple topics and are therefore usually assigned multiple codes. For example, if a single paragraph explains a cultural theory about plants, animals and disease, then the paragraph would be coded with OCM codes for ‘ethnobotany’ (824), ‘ethnozoology’ (825) and ‘theory of disease’ (753).

We searched the Probability Sample Files for 68 OCM codes that could plausibly result in descriptions of *conceptual* knowledge in social or natural domains, such as ethnometeorology, ethnophysiology and genealogy (see the Supporting Information for a complete list). We narrowed this search using six keywords that refer to highly knowledgeable experts in those domains, such as ‘expert*’, ‘specialist*’ and ‘practitioner*’ (where the ‘*’ is a wildcard that would match any suffix). We did not include OCM codes or keywords that referred to specific skills, such as woodworking or boatmaking. Focusing on knowledge about the social and natural world, we also did not include OCM codes relating to religious or spiritual leaders in our search terms (but did not exclude them or expertise in supernatural domains from our results). See the Supporting Information for our full search parameters. This search resulted in 1595 paragraphs from 483 documents.

### 2.1. Inclusion criteria for text records

Many OCM topics are quite broad, and some paragraphs did not contain any information that was relevant to ethnoscientific expertise. If a description was relevant to ethnoscientific expertise, then we included the contiguous set of paragraphs of which it was part, which we refer to as a ‘text record’ henceforth. Because our primary aim was to collect text records about ethnoscientific expertise, we included text records from the ethnographic literature based on two key criteria, which we set out prior to searching: both (1) evidence of ethnoscientific knowledge and (2) evidence of expertise. In this section, we clarify our inclusion criteria.

First, and for the purposes of including vs. excluding text, we defined *ethnoscientific knowledge* as conceptual systems where principles about the natural or social world are socially or individually acquired. Although knowledge can be usefully applied to a number of possible types of practical applications (e.g. curing/healing, certain crafts, hunting/trapping, conflict resolution, ethical quandaries), the OCM codes in our search prioritised the underlying conceptual theories that *can* be applied (rather than descriptions of applications themselves).

Second, as evidence of *expertise* we considered indications of within-group variation in knowledgeability that included descriptions of ‘experts’, or individuals who were highly knowledgeable compared with others. If a text record described an expert individual or a specific group of experts, then it met this criterion. If a text record was vague about individual or within-group variation (e.g. ‘the Maasai are expert herders’), then it did not meet this criterion and was therefore excluded. Expert knowledge might be specific to a single domain such as plants, animals, meteorology or social exchange, but it might also be general and include multiple distinct knowledge domains possessed by a single expert and/or multiple types of expert. See the discussion of our coded variables in the Supporting Information for details.

Practical skills such as hunting, herding, agriculture or conflict resolution were not included in our search terms, but did appear in search results. If a conceptual knowledge domain was included in our search (e.g. ethnozoology) and was frequently linked to text records about a skill that was not included in our search (e.g. hunting), then we retained these records because they were an informative result about that knowledge domain being commonly applied to hunting, rather than a simple result of ‘hunting’ being included in the search.

The final dataset contained 547 text records discussing ethnoscientific experts and specialists from 257 documents (e.g. books, articles) and 55 cultures.

### 2.2. Operationalising and coding evidence for our theoretical models

Each theoretical model was operationalised as a set of coded variables. We coded each text record on the presence or absence of evidence for each of the variables in our theoretical models and its domain(s) of conceptual knowledge. Domains involved the conceptual knowledge in our search terms, as well as motor skills and other additional domains found in our results (i.e. not included in our search terms). For example, for an ethnozoology expert with exceptional hunting skill, we coded ‘ethnozoology’ and ‘hunting’ as expertise domains despite only the former being included in our search terms.

We simplified the wide range of domains in our final dataset by additionally coding each domain of expertise in each text record on three non-mutually exclusive, high-level *domain types*: conceptual, motor skill-related and/or medicinal domains. Because our inclusion criteria were based on the presence of ethnoscientific expertise, conceptual knowledge was included to some extent in each text record. Nevertheless, there was considerable variation in the extent to which text records described conceptual knowledge. A text record's domain of expertise was therefore coded as ‘conceptual’ only if the domain *primarily* involved conceptual knowledge (the *conceptual* domain type refers to ethnoscientific conceptual domains, largely designated by the eHRAF OCM codes; see the Supporting Information for details). The distinction between conceptual vs. motor skills was motivated by how observable a skill might be: motor skills are observable, whereas conceptual domains often are not. The medicinal domain was also included as a domain type because it was both recurrent in the literature and highly inclusive (e.g. herbalists, pharmaceutical experts, diviners and curing specialists). Importantly, these domain types often co-occurred in single text records. For example, experts with motor skills such as boat makers, construction specialists and woodworkers often had high levels of ethnobotanical knowledge, a conceptual domain. See the Supporting Information for more details about the overlap among conceptual, motor and medicinal domain types.

**Figure 1.**
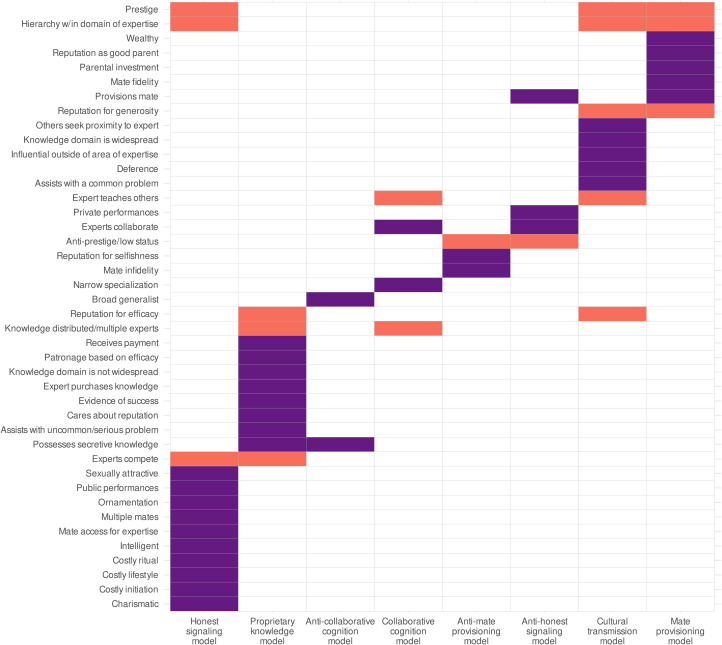
Coded variables corresponding to predictions outlined in our theoretical models. Variables are listed along the *y*-axis, and each theoretical model is listed with its opposing model along the *x*-axis. Filled cells indicate which variables are included in each theoretical model. Purple cells indicate a variable that is unique to one theoretical model (model-specific) and orange cells indicate a variable that is general to multiple theoretical models (model-generic).

We coded each text record for presence/absence on each of the 42 variables operationalising our five theoretical models (described above and detailed in the Supporting Information). See Figure 1 for a list of these variables and their relationships to each theoretical model. Specifically, for each variable and each text record, we coded 1 if there was evidence for the variable and 0 if there was no evidence for the variable. Some text records had evidence against certain variables. For these variables, we therefore created a complementary ‘anti-variable’ indicating evidence against that variable (e.g. low status experts were coded as evidence against prestige, or ‘anti-prestige’). We term the set of anti-variables complementary to a theoretical model its ‘anti-model’. We also included variables for age, sex and case vs. cultural model. The latter indicated if a text record discussed specific individuals who were experts (cases) and/or a general description of domain experts in that culture (cultural models). Finally, we coded two additional *ad hoc* variables that struck the coders as important but were not part of our theoretical models: religious leaders and teaching among kin. The former characterised many experts. The latter was an important special case of the *cultural transmission model* variable *experts teaching others* (but kinship is not a feature of the *cultural transmission model*, so we did not include this variable in the *cultural transmission model*). See the Supporting Information for coding examples and other details.

The first and second authors independently coded for presence/absence (1/0) of evidence for each variable on each text record. The second author was familiar with relevant theories but blind to specific hypotheses in this study, and the first author was not blind to the study hypotheses. Results of the independently coded datasets were an 88.1% match with a Cohen's kappa indicating moderate agreement (*κ* =0.48). See the Supporting Information for more details about interrater reliability. Afterwards, both coders discussed and reconciled all disagreements to produce the coded dataset used in our analyses.

**Figure 2.**
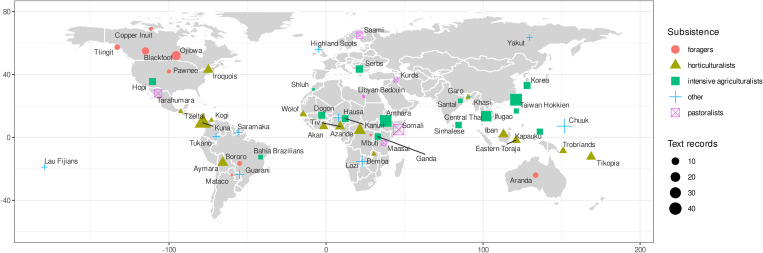
Geographic region of each culture included in our dataset. Colours and shapes indicate subsistence strategy for each cultural group, and sizes indicate the number of text records for each culture in our dataset.

Finally, each of the Probability Sample Files cultures in our search was coded for geographic region and mode of subsistence, which we obtained from the eHRAF (shown in Figure 2).

### 2.3. Statistical analyses

Our data comprised a 547 row by 42 column binary matrix of 0′s and 1′s, where each row was one text record and each column was one variable (0, no evidence; 1, evidence for). Each text record belonged to one culture (i.e. no text records discussed multiple cultures); thus, text records were nested within authors, who were nested within cultures.

Our first analytical goal was to explore which variables clustered together (i.e. variables for which evidence tended to co-occur in text records), as a means of assessing the extent to which the structure of the data corresponded to our theoretical constructs. Our second analytical goal was to more formally assess the extent to which the data supported each theoretical model by determining the level of evidence for each variable (i.e. the mean of each column), the proportion of cultures with at least some evidence for each variable and the total evidence for each model (as described in detail later). Our third analytical goal was to explore the extent to which the variables and each of the theoretical models were associated with three broad types of knowledge: medicinal, motor skills and conceptual knowledge domains.

#### 2.3.1. Goal 1: exploring the structure in our dataset

To explore inherent structure in our entire binary data matrix, i.e. ignoring our *a priori* theoretical models, we used two different clustering methods.

In the first clustering method, we visualised the entire binary matrix with a heatmap (dark colour = 0; light colour = 1). We ordered the rows and columns, so that ‘similar’ rows were adjacent and ‘similar’ columns were adjacent. Similarity between two row vectors or two column vectors was defined as the angle between the vectors when projected onto the first two principal components of the entire matrix, which approximates the correlation between the vectors. Specifically, the ordering was determined by a principal components analysis (PCA) angle seriation method (Hahsler, Buchta, & Hornick, 2021).

To use row ordering as an example, this method conducts a PCA on the row vectors, projects each row vector onto the first two principal components and then orders each row by the angle between the row vector and the first principal component (smallest to largest; see Friendly, 2002). It then splits this ordering by the largest ‘jump’ between adjacent angles (specifically, the absolute value of the difference between adjacent angles), and rearranges these two split orderings so that the largest jump is at the end of the entire ordering. Column ordering is similarly determined, but the PCA is instead conducted on the column vectors. The result is that similar rows (text records) and columns (variables) in the heatmap are adjacent (Friendly & Kwan, 2002; Hahsler et al., 2021).

The second clustering method is based on another measure of similarity among our variables. Here, we computed the square matrix of all pairwise binary distances between column vectors, where binary distance is defined as the proportion of element pairs (i.e. (0, 0), (0, 1), (1, 0) or (1, 1)) in which only one element = 1, amongst the pairs in which at least one element = 1. A binary distance = 0 therefore means that two variables both had evidence in exactly the same text records. A binary distance = 1 means that two variables never had evidence in the same text records. The resulting matrix can be conceptualised as an adjacency matrix, which defines a weighted graph *G*, where each vertex is a variable and each weighted edge is the distance between these variables.

We then computed the minimum spanning tree of this distance matrix, a subgraph of *G* in which every node is connected in a single path that minimises the total weighted path distance without creating any loops (i.e. with no closed paths; Dijkstra, 1959; Prim, 1957; Zahn, 1971). As a result, only similar variables (the vertices) are connected to each other in the minimum spanning tree. We then identified ‘clusters’ of variables by visual inspection of the minimum spanning tree, seeking groups of adjacent variables that were conceptually related. Given the subjectivity of cluster identification, we performed this step after completing the remaining goals.

#### 2.3.2. Goal 2: assessing how the evidence supports different theoretical perspectives

Each theoretical model was operationalised as a set of coded variables. These sets of variables overlapped to some degree, indicating overlap between the theoretical models. To assess each theoretical model, we determined the proportion of text records that supported each of its binary variables (i.e. the proportion of 1′s). Because multiple text records often came from the same document, and multiple documents often reported on the same culture, the text records from an ethnographer who focused on, e.g. uncommon and serious medical problems, would have a misleadingly high proportion of evidence for the ‘uncommon and serious problem’ variable. It was therefore necessary to account for the hierarchical structure of our data.

Specifically, we fit an intercept-only generalised linear mixed effects logistic regression model (GLMM) for each binary variable, with random intercepts for authors nested within cultures. We fit 42 models, one for each binary variable, using all 547 data points, to predict for each text record whether the variable was 0 or 1, adjusting for the structure of the data. The model structure for each variable's proportion of text record-level evidence was therefore as follows:
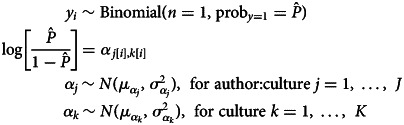
where the value of *y_i_* (and its 95% confidence interval) represents the proportion of text records with evidence for a given variable *i*, adjusted for the hierarchical structure of the data.

To compare theoretical models, we computed a ‘total model score’ as the proportion of evidence for the model in each text record. Specifically, we summed the model variables in each text record and divided by the number of variables. The ‘weight’ of this value was the number of variables. For example, the *mate provisioning model* has eight variables. The total score for this model in each text record was the sum of these variables in each text record, divided by 8 (with weight = 8). This proportion was the outcome variable. The mean proportion and its 95% confidence interval were computed identically to the GLMM structure shown above. Theoretical models with a higher total model score were judged to have more support. (Note that the ‘anti’ models, i.e. the models shown in Figure 1, with variables that refuted its corresponding theoretical models, were analysed identically to the theoretical models.)

We also examined evidence for each variable at the culture level, i.e. the simple proportion of cultures with at least one text record supporting each variable, with 95% confidence intervals for the proportion computed with cluster bootstrapping (i.e. first resampling cultures, then resampling authors within cultures). We converted all proportions and their confidence intervals into percentages, which we report as the variable's level of support.

#### 2.3.3. Goal 3: exploring how support for variables were associated with presence of domain types

At the text record level only, we explored the extent to which evidence for our three high-level domain types (conceptual, motor and medicine) was associated with evidence for each of our 42 variables (e.g. prestige, teaching, secretive knowledge). Specifically, we fit three logistic regression models with our three high-level domain types as binary outcome variables and, in each model, our theoretical model variables as predictors. (Prior to fitting, we removed variables that were >95% zeros, i.e. for which there was almost no evidence; otherwise these variables had spuriously large estimates. See the Supporting Information for more details, and a discussion of our rationale and filtering process.) Because inclusion of many predictors risks overfitting, we used *elasticnet* regression (Friedman et al., 2010), a popular type of penalised regression that was developed for use in situations where the number of predictors, *p* is large relative to the number of observations, *n* (see Supporting Information for a brief description). We used the ‘lasso’ penalty, which sets some coefficients to 0, with the non-zero coefficients representing the ‘best’ predictors, given the limitations of the data. As in a standard logistic regression, the coefficients are log odds, which we transformed to odds ratios, i.e. the ratio of the odds that a text record has evidence for the outcome (domain type) if it has evidence for the predictor variable to the odds that it has evidence for the outcome if it does not have evidence for the predictor variable. This analysis will therefore identify three subsets of variables that are most closely associated with the conceptual, motor and medicinal domains. Note that this model cannot include random effects (i.e. cannot adjust for the hierarchical structure in our data).

We then assessed the association of our five theoretical models with the three domain types as follows. First, we computed text record-level model scores as the proportion of evidence for each model in each text record. For example, the *collaborative cognition model* comprised four variables. If a text record had evidence for all four *collaborative cognition model* variables, its text record-level *collaborative cognition model* model score was 1; if it had evidence for two *collaborative cognition model* variables, its *collaborative cognition model* score was 0.5. We computed text record-level model scores similarly for the other four theoretical models.

We then fit three generalised linear mixed effects logistic regression models, each predicting the presence/absence of evidence for one domain type (medicinal, motor and conceptual) as a function of the five text record-level model scores. Similar to the GLMM described above, we included random intercepts for authors nested within cultures. The estimated coefficients of these three models would then represent the association between the evidence for each theoretical model in each text record with the evidence for the domain type in each text record.

All data and analysis code are available at: https://github.com/alightner/conceptualExpertsHRAF.

## 3. Results

The dataset contained 547 text records, and we found evidence supporting one or more variables from our theoretical models in 528 (97%) of these records. Each text record had an average of five variables coded as present, with 95% of text records containing evidence for 10 or fewer variables (median was 4 present per text record, standard deviation = 3.3, minimum = 1, maximum = 25). The geographic distribution of the cultures in this sample, along with their subsistence strategy and number of text records, are shown in Figure 2. We did not find evidence for ethnoscientific expertise in five of the 60 cultures in the Probability Sample Files. Text records per culture ranged from 1 to 46 with a median of seven. In total, our text records included 115 cases describing specific experts and 473 cultural models, i.e. general descriptions of experts. Publication dates of the 257 documents from which the text records came ranged from 1704 to 2000, with 99% of documents published during the twentieth century (median year was 1968). See the Supporting Information for details and text analyses.

Sex was unknown in 55% of all text records. Of the 45% of text records that identified at least one sex, 82% included males (37% of all text records), 42% included females (19% of all text records), 22% included both males and females within the same text (10% of all text records) and 15% described specialist roles that were exclusive to males and/or females (7% of all text records). (About 2% of all text records described exclusively male experts, and about 2% described exclusively female experts.) Individuals pursuing expertise in ethnoscientific domains were described as older or elderly adults in 13% of all text records, whereas 5% described younger adults and 4% described children or adolescents. Age was unknown in 86% of our text records (for age categories, the percentages in each category and the percentages of uninformative records add to values greater than 100, because some text records described experts in multiple age categories within the same record).

### 3.1. Exploring structure in the data matrix

**Figure 3.**
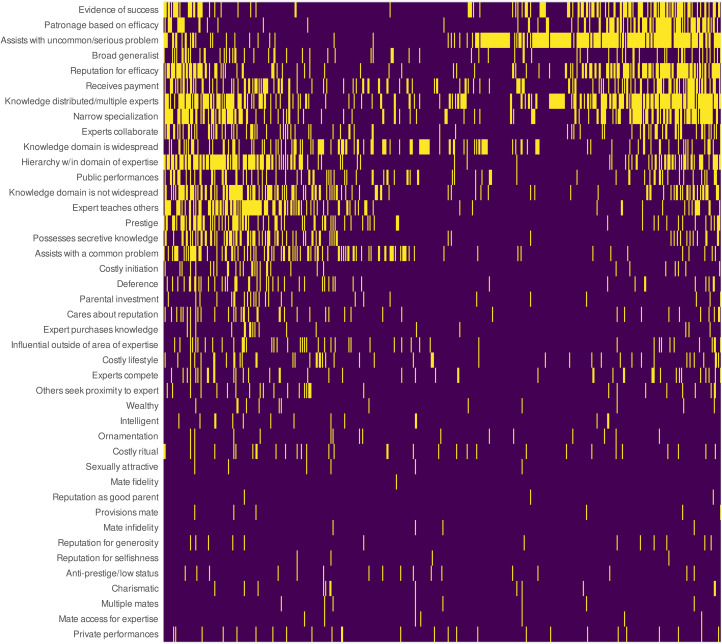
Heatmap visualising the coded dataset based on presence (light cells) vs. absence (dark cells) of evidence for each variable in each text record. For readability, the dataset shown here is transposed, i.e. each row represents a variable and each column represents a single text record. Rows and columns were ordered using the principal components analysis angle seriation method. See text for details.

We visualised our dataset with a heatmap, ordering the rows and columns using the PCA angle seriation method (Hahsler et al., 2021). See Figure 3. The heatmap revealed that there was considerable evidence for some variables (top) and much less evidence for others (bottom). The seriation method also shows two partially overlapping clusters among our well-supported variables. In the left cluster of text records, expertise includes hierarchies in knowledge or skill level, widespread knowledge, assistance with common problems or activities, and experts who teach other people their skill. In the right cluster of text records, expertise includes assistance with uncommon and serious problems, patronage based on efficacious services provided by experts and evidence of success in an expert's task domain. These clusters of text records are somewhat interpretable as primarily relating to the *cultural transmission model* (left) and the *proprietary knowledge model* (right), although it is worth noting that they overlap with each other, and the left cluster is diffuse and includes high levels of support in a number of columns that are not specific to the *cultural transmission model*. Also, the partial overlap between these clusters hinges, in part, on reputations for efficacy and distributed experts among multiple experts. The latter, which was well supported in both of these clusters, was central to the *collaborative cognition model*.

### 3.2. Knowledge domains and types of skill

**Figure 4.**
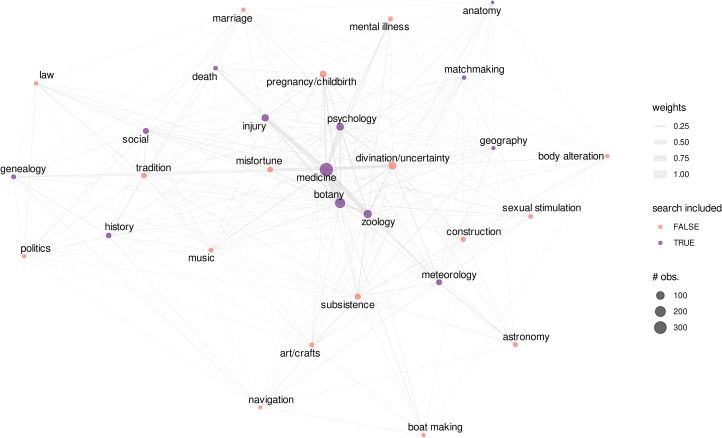
Graph representing commonly occurring domains of knowledge and skill that occurred in text records in our dataset. Vertices indicate domains that occurred in at least 10 text records, and vertex size corresponds to the number of text records including that domain. Vertex colours indicate whether or not the domain was included in our original search query. Each edge indicates that a pair of knowledge/skill domains co-occurred in at least one text record. Edge widths correspond to the frequency with which each domain pair co-occurred (as determined by the number of text records describing them together, normalised by the maximum frequency = 113).

Our search for ethnoscientific experts yielded a variety of knowledge and skill domains, many of which were not included in our search query. Among the conceptual knowledge domains that we included in our search, ethnomedical specialists, largely resulting from our search for theories of disease, were the most common. Expertise in ethnobotany, ethnozoology, ethnopsychology and healing injuries was also relatively common, and frequently co-occurred in text records describing medicinal specialists. However, some knowledge or skill domains that we did *not* include in our search query reliably co-occurred with domains that we did include. Text records describing medicinal specialists, for example, often included descriptions of divination, which was not specified in our search terms. See Figure 4.

Some domains tended to co-occur more frequently than others: medicine, ethnobotany and ethnozoology often co-occurred, for example, forming a ‘cluster,’ but medicine, ethnopsychology and divination also frequently co-occurred, forming a different ‘cluster’. Social expertise, an inclusive category motivated by the ethnosociology OCM code (e.g. conflict resolution, intergroup relations), similarly clustered with traditional domains, such as mythology and norms of behaviour, and with traditional history and law. Skills relating to a culture's subsistence strategy (not included in our search query) clustered with ethnozoology, ethnobotany and ethnometeorology, and often described the use of knowledge to improve subsistence outcomes (e.g. ethnozoology among hunter–gatherers, ethnobotany among agriculturalists, and ethnometeorology among pastoralists and horticulturalists; see Figure 4).

### 3.3. Theoretical model results at the text record and culture levels

**Figure 5.**
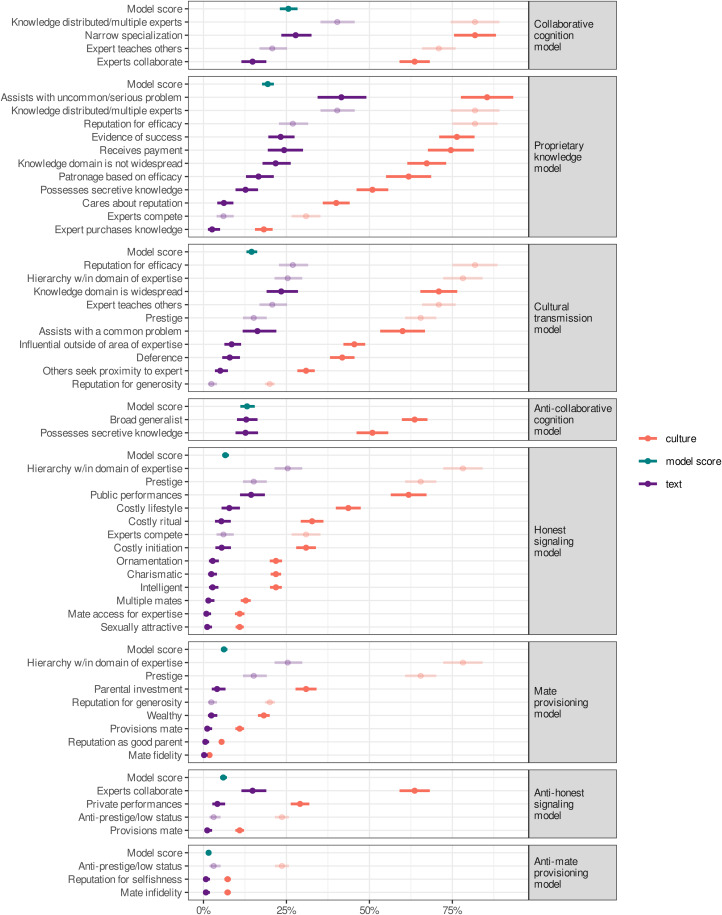
Support for each variable, faceted by theoretical model. Points represent the percentage of evidence for that variable (the fixed-effect intercept from a generalised linear mixed effects model), and colours indicate whether that percentage is at the level of text record (percentage of text records with evidence), culture (percentage of cultures with evidence) or total model score (percentage of text records with evidence for any variable defining a given model). Solid colours indicate variables that are specific to theoretical models, whereas faded colours indicate variables that are generic, i.e. included in more than one theoretical model. Error bars are 95% confidence intervals of the fixed-effect intercept from a generalised linear mixed model, with random intercepts for author nested within culture.

Support for each variable was determined by the percentage of text records containing evidence for it (text record level support) and the percentage of cultures containing at least one text record with evidence for each of the same variables (culture level support). Each ‘total model score’ was the percentage of text record level support across all variables defining a model. (See the Methods section for details.) As indicated in Figures 1 and 5, some variables were consistent with multiple theoretical models, and were therefore included in multiple total model scores. As we also show in Figure 1 and the Methods section, some text records contained evidence that explicitly refuted a theoretical model, which we coded as an anti-model. Anti-models were analysed identically to the other theoretical models.

Based on the total model scores, the *collaborative cognition model* received the highest level of support at the text record level (25.6%), but it also had considerable evidence against it (13.1%). The *proprietary knowledge model* and *cultural transmission model* were both relatively well supported and made largely distinct predictions, indicating mixed support for each (*proprietary knowledge model*, 19.4%; *cultural transmission model*, 14.5%). Although the *honest signalling model* and *mate provisioning model* received similar but relatively low levels of support (*honest signalling model*, 6.6%; *mate provisioning model*, 6.2%), evidence against the *honest signalling model* and *mate provisioning model* was also relatively low (anti-*honest signalling model*, 6%; anti-*mate provisioning model*, 1.5%). See Figure 5 for text record level support, culture level support and total model scores. At the culture level, we found no meaningful variation in model scores by geographic region or subsistence strategy. See the Supporting Information for details.

### 3.4. Exploring associations between the variables and conceptual, medicinal and motor domains

At the text record level, we fit three elasticnet logistic regression models to explore which model variables in Figure 3 (as predictors) were associated with evidence for each of our three high-level domain types (as outcomes), i.e. medicinal domains, conceptual domains (e.g. ethnobotany, ethnometeorology, genealogy) and motor skill-related domains (e.g. construction, boat making, art/crafts).

**Figure 6.**
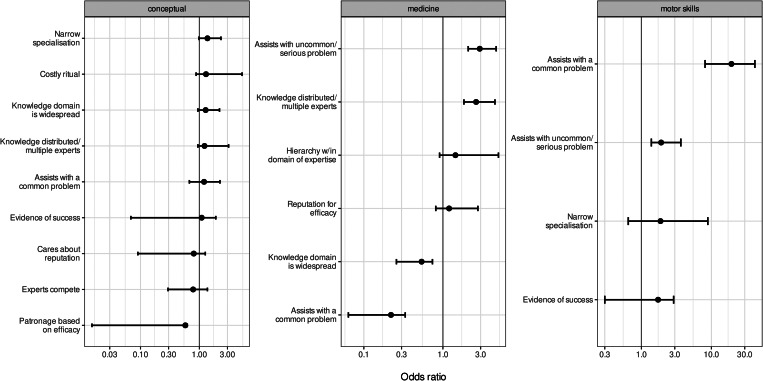
Coefficients for the ‘best predictors’ of each domain type in our three elasticnet logistic regression models. Each facet shows the coefficients of each regression model. Each domain type, shown in the facet labels, was the outcome variable, and each variable along the *y*-axes was a best predictor in its regression model (i.e. had a non-zero coefficient). Regression coefficients are reported as odds ratios (*x*-axes), and error bars are 95% confidence intervals. Note that each *x*-axis is log-scaled.

The coefficients of each of our three elasticnet regression models are shown in Figure 6. The elasticnet regression models showed two key results. First, evidence of experts assisting with routine or common problems was strongly associated with individuals working in motor skill-related domains, such as woodworking, crafting and subsistence. (To a much lesser extent, motor skill-related domains were also positively associated with some uncommon and serious problems, such as bone-setting after an injury.) Second, medicinal domains were positively associated with assistance with uncommon and serious problems, and positively associated with highly specialised knowledge among multiple types of expert. Medicinal domains were negatively associated with widespread or readily accessible knowledge, and in contrast with motor skills, medicine was also negatively associated with assistance with common problems. A handful of variables were weakly positively or negatively associated with conceptual domains, which frequently overlapped with medicinal domains and, to a lesser extent, motor skill-related domains (Figure 4). However, no clear pattern emerged with conceptual domains as the outcome variable (Figure 6).

### 3.5. Exploring associations between the theoretical models and conceptual, medicinal and motor domains

**Figure 7.**
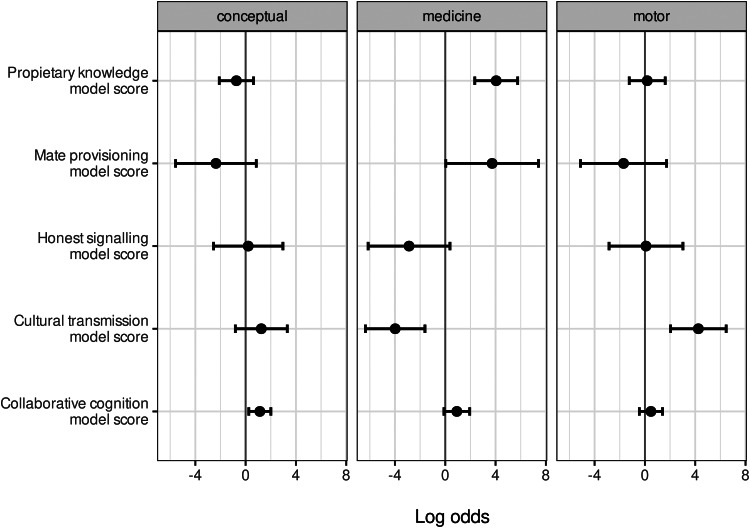
Regression coefficients for three generalised linear mixed effects logistic regression models of each domain type (conceptual, medicine, motor) as a function of theoretical model scores at the text record level. Theoretical model names are listed along the *y*-axis and domain types are shown in the facet labels. Estimates are reported in log odds, and are shown on the *x*-axis. Error bars are 95% confidence intervals.

To assess associations of our five theoretical models with each of the three domains, we first computed text record-level models scores for each of our five theoretical models to determine how well each text record supported each model (see Methods section). We then fit three separate GLMMs, with domain types as outcomes and text record-level model scores (*collaborative cognition model*, *cultural transmission model*, *proprietary knowledge model*, *honest signalling model* and *mate provisioning model*) as predictors. The results of these regression models showed that the *cultural transmission model* was positively associated with motor skill-related domains and negatively associated with medicinal domains, whereas the *proprietary knowledge model* was positively associated with medicinal domains (Figure 7).

### 3.6. Minimum spanning tree: exploring structure among the variables

**Figure 8.**
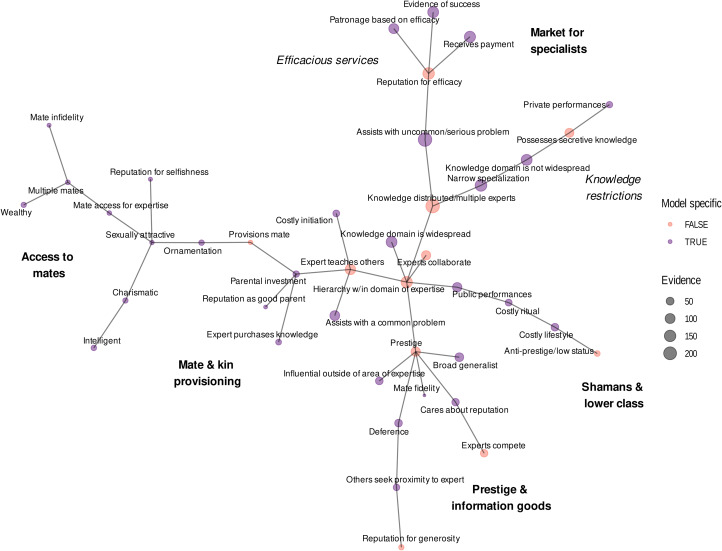
Minimum spanning tree of the variable binary distance matrix. Vertices represent variables, vertex sizes correspond to levels of text record support for each variable and vertex colours to whether or not the variable is model specific vs. model generic. Edge lengths represent binary distances between variables. Annotations refer to our interpretations of each cluster.

In our minimum spanning tree, variable clusters only partially mapped onto our *a priori* theoretical models. See Figure 8. Specifically, we found a variable cluster that we characterise as a ‘market for specialists’, which includes two subclusters: efficacious services and knowledge restrictions relevant to the *proprietary knowledge model*. The variables in this cluster had high levels of evidence across text records (indicated by the size of the nodes).

A second cluster included experts with prestige, deference, reputations for generosity, influence and skill outside of one's area of expertise (i.e. ‘broad generalists’), and expert competition. We interpret this cluster, which also had high levels of evidence across text records, as support for the prestige and ‘information goods’ theories associated with the *cultural transmission model* (Henrich & Gil-White, 2001).

Further, we found variable clusters that resembled aspects of the *honest signalling model* and *mate provisioning model*, although the levels of evidence across text records was relatively low. A final cluster appeared to relate largely to shamans and low-status occupational specialisations. See the Supporting Information for details and examples of text records supporting this interpretation.

We re-ran all of the foregoing analyses separately by sex to explore possible sex-specific patterns. We found no major sex-specific patterns, except that three variables – prestige, public performances and narrow specialisation – were associated with evidence for male experts. See the Supporting Information for a more detailed analysis of sex differences.

## 4. Discussion

In this study, we considered the extent to which multiple evolutionary perspectives on knowledge and skill acquisition explain ethnoscientific expertise, and whether our exploratory results would suggest a new theory of expertise.

Ethnoscientific experts were skilled in a variety of conceptual domains, with medicinal expertise being especially common. Although we restricted our search query and post-search filtering to only include text records describing ethnoscientific expertise, these text records also frequently included discussion of multiple knowledge and skill domains, conceptual and/or motor skill-related, such as boat making, woodworking, subsistence and construction (Figure 4).

Our analyses were generally supportive of three theoretical models developed from the existing literature on the acquisition of knowledge and skill: the collaborative cognition model, the proprietary knowledge model and the *cultural transmission model*. The *proprietary knowledge model* and *cultural transmission model* make some contrasting predictions, however, and the anti-*collaborative cognition model* (evidence against *collaborative cognition model*) also received a moderate level of support. The *collaborative cognition model*, *proprietary knowledge model* and *cultural transmission model* therefore received mixed support overall. We found similarly mixed support for the *honest signalling model* and *mate provisioning model*, but in general there was much less evidence in the text records for these models, and for their counterparts (anti-*honest signalling model* and anti-*mate provisioning model*; Figure 5). The mixed support for our *a priori* models indicates that reformulation is in order.

### 4.1. Toward a data-driven theory of expertise

Our exploratory analyses revealed factors that were associated with greater or lesser support for the three models with greatest support overall: the *collaborative cognition model*, *proprietary knowledge model* and *cultural transmission model*. Here, we distill these insights as a first step towards the development of a more general theory of ethnoscientific expertise.

Our first exploratory analysis revealed two clusters of text records associated with two clusters of variables. The upper left quadrant of the heatmap (Figure 3) mostly involved text records that discussed hierarchies of experts that assisted with common problems in widespread knowledge domains, who had prestige and taught others. This cluster included support for many *cultural transmission model* variables, among others. The upper-right quadrant, on the other hand, mostly involved text records that discussed experts who *assist with serious, uncommon problems* and who were *patronised based on their efficacy*. This cluster included support for many *proprietary knowledge model* variables. There was considerable overlap between these clusters, however, which hinged on high levels of support in each cluster for the presence of experts with *reputations for efficacious services*, *narrow specialisation* and *distributed knowledge among multiple experts*. Distributed knowledge and narrow specialisation were motivated by the *collaborative cognition model*, and their support is consistent with the idea that distributed and complementary social structures are key factors for both widely transmitted knowledge and proprietary knowledge. The *collaborative cognition model*, in other words, seems to serve as a bridge between the *proprietary knowledge model* and *cultural transmission model*.

In the text records, knowledge domains and their characteristics (e.g. hidden and conceptual vs. observable and motor skill-related) varied considerably. Nevertheless, the two foregoing clusters seemed to be distinguished by an emphasis on motor skills (left cluster) vs. medicinal skills (right cluster). We therefore categorised specific expertise domains in each text record (Figure 4) into more general domain types: motor skills, medicinal knowledge and/or our ethnoscientific domains that were primarily conceptual knowledge.

Our elasticnet regression models found that evidence supporting the *cultural transmission model* and *proprietary knowledge model* was largely conditional on these domain types (Figures 6 and 7). Specifically, two variables associated with the *proprietary knowledge model* (*assists with uncommon and serious problems* and *distributed knowledge across multiple experts*) were positively associated with medicine, whereas two variables associated with evidence for the *cultural transmission model* (and against the *proprietary knowledge model*) – *knowledge domain that is widespread* and *assists with common and routine problems* – were negatively associated with medicinal domains. Evidence for assistance with common problems (from the *cultural transmission model*), such as subsistence and construction, was strongly associated with motor skill-related domains. Assistance with uncommon and serious problems was also associated with motor skill-related domains (albeit to a much lesser extent), but these text records involved bone-setting and similar types of injury healing (see Figure 6).

Aggregating text record-level evidence for each variable into total model scores showed a similar trend: support for the *cultural transmission model* was positively associated with expertise in motor skill-related domains, whereas support for the *proprietary knowledge model* was positively associated with expertise in medicinal domains (Figure 7). In general, medicinal domains were occasionally linked to motor skill-related domains, such as injury healing, but they were far more often linked to conceptual and non-motor skill-related domains, such as botany, psychology, and more surprisingly, divination during times of uncertainty (Figure 4).

Our finer-grained exploratory analysis of structure in our data matrix, using a graph-based clustering method (minimum spanning tree), further clarified the relationship between the *proprietary knowledge model*, *collaborative cognition model* and *cultural transmission model* seen in our heatmap. In the minimum spanning tree, our variables clustered in ways that largely, but not completely, corresponded to our theoretical models (Figure 8). A large and relatively well-supported cluster contained associations among prestige, deference, reputations for generosity and high competence outside of one's area of expertise (termed ‘broad generalists’). We interpreted this cluster as support for the ‘prestige and information goods’ theories associated with the *cultural transmission model* (Henrich & Gil-White, 2001). A separate cluster, which was also well supported, resembled what we call a ‘market for specialists’, which itself included two subclusters: efficacious services and knowledge restrictions that are relevant to the *proprietary knowledge model*.

Social dimensions of expertise, some part of the *collaborative cognition model*, served as ‘hubs’ linking the *cultural transmission model* and *proprietary knowledge model* clusters together: distributed expertise among multiple complementary expert roles and knowledge and skill hierarchies were each situated between the *market for specialists* and *prestige and information goods* clusters. Collaboration among experts was directly adjacent to these hubs, and occurred more frequently than competition overall (competitive experts in 8% of text records vs. collaborative experts in 15%).

The *skill level hierarchy* hub of the minimum spanning tree also connected the branches that largely corresponded to the *honest signalling model* and *mate provisioning model*. These are labelled as ‘access to mates’ and ‘mate and kin provisioning’ in Figure 8, respectively, and are difficult to draw inferences from given the low levels of evidence for their constituent variables.

Interestingly, a separate cluster that our theoretical models did not anticipate appeared to relate largely to shamans and low-status occupational specialisations. (See the Supporting Information for details about how we arrived at this interpretation.) Although this particular cluster consisted of low levels of support, ethnoscientific experts were also religious or spiritual leaders, such as priests or shamans, in 19% of all of our text records. Future research will further address this trend by investigating how religious leadership and ethnoscientific expertise might share a common evolutionary explanation (Lightner, Heckelsmiller, and Hagen, in preparation; see also Garfield, Syme, & Hagen, 2020).

We now focus on the major *market specialists* and *prestige and information goods* branches, and their linking hubs, to more thoroughly evaluate their theoretical implications and interrelationships, in light of the domain specificity revealed by our exploratory analyses.

### 4.2. A market for specialists

The *proprietary knowledge model* was more associated with ethnomedical specialists who were consulted during a crisis, sometimes using divination (Figure 4), whereas the *cultural transmission model* was associated with observable motor skills, knowledge that is widely distributed in lesser forms among the population (beyond the experts) and commonly occurring problems (Figures 3, 6 and 7).

If a problem is rare but serious, then for the average individual, the cost of learning to resolve it might be greater than the cost of paying a specialist to do so, if and when that problem arises. Outsourcing uncommon but serious problems creates a demand for solutions, and thus, a market niche for specialising in those solutions. Specialised knowledge can therefore allow some individuals to gain a fitness advantage (e.g. prestige, material resources, beneficial partnerships) in exchange for their services or *products* (Hammerstein & Noë, 2016; Price & Van Vugt, 2015; Sugiyama & Sugiyama, 2003; Tooby & Cosmides, 1996), what Hagen and Garfield (2019) refer to as computational services. If the market value of those products is undermined by sharing the underlying know-how used to generate them, then a beneficial strategy for specialists would be to keep their knowledge hidden, or proprietary.

This creates an apparent contradiction: the existence of cumulative cultural knowledge in *any* type of domain, proprietary or not, presupposes transmitted knowledge (Legare, 2017; Tennie, Call, & Tomasello, 2009). That is, cultural evolution literature correctly emphasises that transmitted knowledge is imperative for cumulative culture (Boyd & Richerson, 1996; Boyd et al., 2011; cf. Pinker, 2010). As our results clarify, however, it would be a mistake to conclude that transmitted and proprietary knowledge are at loggerheads, or that evidence for the *proprietary knowledge model* is evidence against the *cultural transmission model* (and vice versa). Instead, as we argue next, the relative importance of the *cultural transmission model* and *proprietary knowledge model* depends on properties of an expert's task domain.

### 4.3. The relationship between transmitted and proprietary knowledge

Under the *cultural transmission model*, there is one type of social transmission: a larger pool of naive individuals observes a smaller pool of skilled individuals, perhaps in exchange for deference, thus acquiring their skills. Under the *proprietary knowledge model*, in contrast, there are two types of ‘transmission’: first, there are the services (products) that a few experts provide to a large pool of customers in exchange for some kind of payment, e.g. doctors’ diagnoses and treatments to patients. This does not result in much, if any, increase in specialised knowledge by customers (e.g. patients do not gradually become doctors). Second, experts expend considerable effort training a small pool of future experts (again, perhaps in exchange for some kind of much larger payment or inclusive fitness benefit). Indeed, consistent with the *cultural transmission model*, experts were also teachers in 21% of our text records, and this was closely related to assistance with common problems (Figures 3 and 6). Of these observations, however, 11% involved purchased knowledge and 37% appeared to involve teaching among kin. These constraints on social learning are amenable to a variety of interpretations, but in any case they are consistent with the *proprietary knowledge model* proposal that know-how is a valuable resource, and might not be unconditionally shared. Questions about the roles of payment and nepotism in parting with valuable know-how can be explored in future research, but these findings do suggest that, in practice, a spectrum of expertise lies between the *cultural transmission model* and *proprietary knowledge model*.

Although the *proprietary knowledge model* does not rule out transmission, a possible concern about its constraints on transmission is knowledge loss, undermining the scope for cumulative culture. Task domains supported with the *proprietary knowledge model* are not commonly encountered, which by definition means that they are sampled rarely and provide fewer opportunities to learn (Strimling, Enquist, & Eriksson, 2009). For example, Reyes-García et al. (2013) found that medicinal knowledge was susceptible to knowledge loss among Tsimane forager–horticulturalists over time, whereas motor skill-related domains such as boat making and construction tended to either remain consistent or increase over time.

However, there are reasons to doubt that this concern is general to all types of skill domains. While much focus in cultural evolutionary theory is on behavioural copying, it is worth making explicit *how* knowledge in a particular domain is transmitted. One key difference between conceptual and motor skill-related domains is the degree to which information is public vs. private. Many motor skill-related domains, such as technological tasks, are achieved through specific, well-defined action sequences that can be observed and copied with high fidelity, even when underlying know-how is causally opaque (Flynn & Smith, 2012). In contrast, conceptual knowledge comprises mental representations, some of which are more easily and reliably constructed than others (Boyer, 1998; Sperber, 1996). Learning tools, such as ostensive communication, intuitive analogies and mnemonic devices, are available means for communicating conceptual knowledge, but these rely on reconstructive processes rather than high-fidelity copying (Acerbi & Mesoudi, 2015; Morin, 2016).

Our study suggests that, among experts, a tendency to broadly vs. conditionally share knowledge depends strongly on the type of knowledge/skill domain, i.e. common problems that are solved by acquiring motor skills, vs. rare and serious problems that are solved by acquiring conceptual knowledge. Future research can investigate how high market value of services might be associated with proprietary knowledge that is reluctantly shared or exchanged for a benefit (but see Lewis, 2015). A relevant factor for choosing whether or not to share knowledge might also be its scope for monopolising valuable services. An alternative hypothesis about knowledge loss, consistent with our account here, might be that some socioecological changes (e.g. market integration, developing clinics and infrastructure) introduce novel or expanded markets of knowledgeable specialists on whom individuals can rely for efficient and efficacious solutions in a given domain (Salali et al., 2020).

### 4.4. The distribution of cognitive labour

A central feature of the *collaborative cognition model*, which bridged the *cultural transmission model* and *proprietary knowledge model*, is a distribution of cognitive labour: multiple experts have elaborate but incomplete knowledge about their own domains of expertise and rely on others to share knowledge about similarly partial expertise in complementary areas (Heintz, 2004; Keil, 2003). An economic exchange of ideas, on this view, enables cumulative cultural knowledge among highly interdependent specialists, permitting mutually beneficial increases in group-level knowledge. This might suggest that for specialists in conceptual domains, the market value of know-how is based on its rarity. Specialists can therefore use their comparative advantage to beneficially trade their (otherwise proprietary) innovations for other innovations outside of their areas of expertise (Tooby & Cosmides, 1996).

On the other hand, the market dynamic described here at least partially resembles existing perspectives on group size and cultural innovations, where a larger number of specialists exchange know-how with each other and improve their overall scope for innovation (Henrich et al., 2016; cf. Vaesen, Collard, Cosgrove, & Roebroeks, 2016). Some empirical evidence has supported a relationship between group size and cultural innovations (Derex, Beugin, Godelle, & Raymond, 2013; Kline & Boyd, 2010), but these might be general to non-cumulative cultural copying, so long as the task at hand is sufficiently easy to copy (e.g. Ashton, Thornton, & Ridley, 2019).

Future research should therefore be vigilant about the different dynamics of cumulative culture in motor domains, which are relatively easy to copy with high fidelity, vs. conceptual domains, which are not. In conceptual domains, cumulative culture might resemble the evolution of scientific concepts, i.e. old ideas used to generate new ones, with lower demands on transmission fidelity and higher demands on sufficiently building up their underlying intuitions (Carey, 2011; Caporael, Griesemer, & Wimsatt, 2014; Wimsatt & Griesemer, 2007). Culture is complex, and when explaining its accumulation it is likely that one size does not fit all.

It is also worth noting that the *collaborative cognition model* does not necessarily represent ‘pure collaboration’ among experts. Instead, it is compatible with the coexistence of collaboration and competition, both of which are compatible with the *proprietary knowledge model* and *cultural transmission model*. (The *cultural transmission model* even clustered with competition among experts in Figure 8.) Indeed, a more restrictive version of the *collaborative cognition model* might have attenuated its emphasis on collaborative expertise, e.g. by including argumentation to gain influence in discourse (Mercier & Heintz, 2014; Mercier & Sperber, 2017) or by emphasising the role of prestige as an incentive for competition among specialists. In small-scale societies, prestige and general competence might be linked to transmitted knowledge and some mix of collaboration and competition: for example, traits that are frequently associated with elected leadership – such as intelligence, high-quality decision-making, prosociality and mentorship (Garfield & Hagen, 2020; Garfield, Hubbard, & Hagen, 2019) – reflect key aspects of the *cultural transmission model*, *proprietary knowledge model* and *collaborative cognition model*. Conversely, more specialised competences often emerge as societies and markets scale up in complexity (Cockburn, Crabtree, Kobti, Kohler, & Bocinsky, 2013; Johnson, 1982). This increased complexity can, for some historical or ecological reason, also lay the groundwork for a mix of collaboration and competition among prestigious groups of specialists, e.g. as seen in the shift from general medical practitioners towards widespread medical specialisation in nineteenth-century Paris (Weisz, 2003).

Open questions therefore remain about how competition among experts with proprietary knowledge is balanced against collaboration among experts, especially when they have complementary areas of knowledge. Do individual interests overlap as a consequence of mutually beneficial epistemic partnerships? What benefits calibrate otherwise conflicting interests, e.g. among apprentices and their acolytes, or possibly among leaders and followers more generally? These questions can be investigated in future research.

### 4.5. Limitations and caveats

Our source ethnographies varied in their theoretical commitments and aims. Some were broadly descriptive, but most focused on specific subjects other than expertise and few shared our theoretical questions. Our search strategy also relied heavily on eHRAF OCM codes. Our sample is therefore not random, but is biased towards the subjects drawing the attention of ethnographers in our dataset, and the paragraph coding schemes used by HRAF staff. This suggests that our sample and its analyses are representative of ethnographic writings about expertise, rather than direct observations of expertise.

Relatedly, an absence of evidence in this study should not be interpreted as evidence of absence. Ethnographers often emphasise the immediately relevant aspects of expertise, such as applications of knowledge, learning, social roles as experts and consequences of being highly knowledgeable. This might account for the high levels of support among the *collaborative cognition model*, *proprietary knowledge model* and *cultural transmission model* compared with the *honest signalling model* and *mate provisioning model*, especially if mating, parenting and resource flows are less observable for, or deemed irrelevant by, the ethnographers. A similar caveat should be applied to the large number of religious practitioners in our results (19% of text records); ethnoscientific concepts might be mistakenly seen as supernatural when the subject matter is abstract or involves invisible entities (Gottlieb, 2004).

The abstract nature of conceptual knowledge also complicated matters. It is difficult to measure and characterise the distribution of knowledge in a population. Compared with direct empirical observations, which themselves face formidable challenges (Kronenfeld, 2011), ethnographic studies are especially imprecise. Drawing inferences from ethnographic texts, especially the relatively short ones in our study, involves a further and inevitable lack of precision. Different variables in our coding scheme have different levels of overlap with each other, they might vary in their inclusiveness and specificity and they are coded based on multiple levels of interpretation. We attempted to minimise these limitations by using two independent coders and reconciling coding differences afterward. Nevertheless, our data were filtered through judgements made not only by an ethnographer (Sperber, 1985), but also by our own interpretations of how the text records related to our coding scheme.

### 4.6. Conclusion

In this study, we investigated the extent to which five mutually compatible evolutionary theories of knowledge and skill acquisition could account for ethnoscientific expertise, using 547 ethnographic text records from 55 geographically diverse societies. We found high levels of support for the *collaborative cognition model*, the *proprietary knowledge model* and the *cultural transmission model*, and low levels of support for the *honest signalling model* and the *mate provisioning model*. Our exploratory analyses revealed that the *proprietary knowledge model* was associated with medicinal knowledge, which was largely conceptual and involved solving rare and serious problems for clients. Conversely, the *cultural transmission model* was associated with motor skill-related knowledge, which involved solving common, everyday problems, such as subsistence and construction. Support for each of these theoretical models was often linked to support for the *collaborative cognition model*, which was broadly supported across knowledge domains. While many evolutionary theories imply competition among experts, our results suggest that collaboration among experts, who share know-how and/or services, is also important.

Taken together, our results suggest that, rather than applying a single theoretical framework across multiple cultural domains, cultural evolutionary theories about ethnoscientific expertise should explicitly focus on the private and conceptual vs. public and observable nature of knowledge and skill domains, and their applications to common vs. rare problems.

## Supplementary material

For supplementary material accompanying this paper visit https://doi.org/10.1017/ehs.2021.31. click here to view supplementary material

## Research transparency and reproducibility

All data and code are available at: https://github.com/alightner/conceptualExpertsHRAF.
