# Supplementary material for: Ethnoscientific expertise and knowledge specialisation in 55 traditional cultures
Source: Evol Hum Sci. 2021 Jun 14;3:e37. doi: 10.1017/ehs.2021.31 (PMC10427309; doi:10.1017/ehs.2021.31)
Supplement: Supplementary file 1 [file S2513843X21000311sup001.pdf]

Supplementary Information for *Ethnoscientific expertise in 55 traditional societies*

Aaron D. Lightner, Cynthiann Heckelsmiller, Edward H. Hagen

Contents

1 Operationalizing our theoretical models 1
1.1 Cultural transmission model . . . 2
1.2 Proprietary knowledge model . . . 4
1.3 Collaborative cognition model . . . 6
1.4 Honest signaling model . . . 7
1.5 Mate provisioning model . . . 9

2 Description of our search query 10
2.1 Inclusion criteria . . . 10

3 Complete description of our coding scheme 11

4 Examples of our coded variables 13

5 Coding our domains into domain types 17

6 Interrater reliability 22

7 Explanation of the elasticnet regression model 22

8 Filtering extremely sparse variables in our elasticnet regression 24

9 Publication dates 25

10 Analyzing the raw text in our dataset 27

11 Raw count data for each variable 32

12 Shamans and lower class individuals in our spanning tree results 34
12.1 Examples of shamans and other spiritual figures in this cluster . . . 34
12.2 Examples of low status occupations in this cluster . . . 34

13 Culture level support for each model 35

14 Analysis of sex differences 38

1 Operationalizing our theoretical models

In this section, we discuss how we operationalized our theoretical models in more detail, including quotations from key references that motivate the inclusion of our model predictions. All of the variables in our coding scheme are associated with, and based on, these predictions and their associated references.

1.1 Cultural transmission model

The cultural transmission model emphasizes the social transmission of knowledge as an indispensible core feature of human evolution. Coded variables supporting and clearly distinguishing the cultural transmission model from others include evidence of experts having relatively high skill in an ability which is, in some form, common knowledge and a solution of a common or day-to-day problem. Others include prestigious and high status experts; reputations for efficacious solutions to certain problems; same-sex deference; experts who have reputations for generosity and/or are preferred social partners beyond their domains of expertise; experts who share knowledge ("know-how") with other experts and/or non-experts, often in the context of mentorship or apprenticeship; experts with influence on others beyond their domain of expertise.

The cultural transmission model emphasizes two closely related ideas from dual inheritance theory, namely, the cultural niche hypothesis and the cumulative cultural brain hypothesis. The Cultural and Cumulative Cultural Brain Hypotheses of Muthukrishna, Doebeli, Chudek, & Henrich (2018) is defined as: "[T]he idea that brains have been selected for their ability to store and manage information via some combination of individual (asocial) or social learning. That is, we develop the idea that bigger brains have evolved for more learning and better learning" (p. 1). Muthukrishna et al. formally model these hypotheses and discuss them in more detail:

> "In contrast to competing explanation, the key message of the Cultural Brain Hypothesis (CBH) is that brains are primarily for the acquisition, storage and management of adaptive knowledge and that this adaptive knowledge can be acquired via asocial or social learning. Social learners flourish in an environment filled with knowledge (such as those found in larger groups and those that descend from smarter ancestors), whereas asocial learners flourish in environments where knowledge is socially scarce, or expensive but obtainable through individual efforts. … The Cumulative Cultural Brain Hypothesis posits that these very same processes can, under very specific circumstances, lead to the realm of cumulative cultural evolution. These circumstances include when transmission fidelity is sufficiently high, reproductive skew is in a Goldilocks' zone close to monogamy (or equally, there is some, but not too much individual-level selection), effective asocial learning has already evolved, and the ecology offers sufficient rewards for adaptive knowledge" (p. 29).

Citing Boyd and Richerson (1995), Muthukrishna et al. go on to state:

> "Our model supports both arguments [by Boyd and Richerson] showing that only high fidelity social learning gives rise to cumulative cultural evolution and that the parameter range to enter this realm expands if social learning is more common. In our model, cumulative cultural evolution exerts a selection pressure for larger brains that, in turn, allows more culture to accumulate. Prior research has identified many mechanisms, such as teaching, imitation, and theory of mind, underlying high fidelity transmission and cumulative cultural evolution" (p. 30).

This is closely related to, and clearly summarized in, the cultural niche hypothesis, which is an alternative to the cognitive niche hypothesis favored in response to Pinker (2010) by Boyd, Richerson, & Henrich (2011):

> "We suggest, instead, that our uniquely developed ability to learn from others is absolutely crucial for human ecological success. This capacity enables humans to gradually accumulate information across generations and develop well-adapted tools, beliefs, and practices that no individual could invest on their own" (p. 10919).

A key feature of the cultural niche hypothesis, Boyd et al. conclude, is that "cultural evolution operating over generations has gradually accumulated and recombined adaptive elements, eventually creating adaptive packages beyond the causal understanding of the individuals who use them" (p. 10923).

In other words, even experts are not highly knowledgeable in a task domain that others lack; rather, they are highly skilled relative to others, but knowledge is widely disseminated via long, intergenerational transmission chains, often via imitation, in the population. This assumption is more clearly featured in Henrich (2004), which makes a number of assumptions about expertise that are consistent with the cultural transmission model:

> “This evidence [of human social learning], from both field and laboratory studies, shows that humans possess a psychological propensity to pay attention to, and attempt to imitate, particularly skillful, successful and/or prestigious individuals. A tendency to orient one’s social learning attention toward particularly skillful individuals (”cultural models“) creates a selective force in cultural transmission that may, under some circumstances, generate cumulative adaptation” (p. 200).

This quotation, in context, leads up to his model in which discrete, transmittable skills are characterized by a quantitative measure of how skillful a person is, and naive learners must copying this skill from more skillful experts in attempts to copy with varying degrees of resulting success (which is usually lower than the skill level of an expert). These skills “might involve such things as net-manufacturing preferences..., spear-throwing techniques, fishhook material selection, canoe-building techniques, bone-tool craft, and medicinal plant knowledge” (p. 200).

Key aspects of this model, which shed light on the relevant assumptions of imitation according to the cultural transmission model, include the parameterization Henrich (2004) uses of the distribution of skill levels in the population. Specifically, he uses a Gumbel distribution of skill levels to model imperfect imitation, such that experts are on the high-valued end of the distribution. The skew is modeled by α, and the dispersion is modeled by β:

> “If something is easy to imitate and people vary little in the inferences they make during the imitation process, then both α and β will be small – for perfect replication α and $\beta = 0$. If something is hard to imitate, but people tend to make the same kinds of mistakes, then α will be large, and β small. If something is difficult to accurately imitate, and people make wildly different inferences/mistakes, then α and β will both be large. If people generally make fairly accurate inferences in learning something, but sometimes diverge wildly in their efforts, α will be small and β large” (p. 201).

The α parameter represents, in other words, the assumption that inferences made by social learners “are biased so that the behaviors acquired by copiers are, on average, less skilled than that of their model”, whereas β reflects the assumption that they “are noisy, so that copiers never accurately replicate the [skill] value of their model” (p. 201). The resulting tradeoff (figure 2 in the Henrich [2004] paper) highlights the resulting necessity of transmission: as the ratio $\frac{\alpha}{\beta}$ increases (i.e., imitation is uniformly difficult and mistakes have low variation), the required population size for maintaining cumulative adaptive evolution – in contrast to maladaptive skill/knowledge loss – sharply increases. As Henrich (2004) describes it:

> “To get an intuitive sense of what is going on here, consider what happens if each learner picks only one person (N=1) and attempts to copy his skills. Under these conditions, learners would, on average, select only a model of average skill to copy, and thus would obtain a worse-than-average set of skills (assuming copies tend to be worse than the original). However, if learners can pick two models and learn from whichever of the two is the most skilled, then learners will (on average) learn from a better-than-average model, but they will still suffer the losses from imperfect inference and imitation. ... Cultural learning becomes cumulatively adaptive when the effect of having a larger set of models from which to pick the most skilled exceeds the losses from imperfect copying” (p. 203).

This necessity of knowledge dissemination and its relevance to expertise is reinforced even more clearly by Kline and Boyd (2010):

> “[S]ocial learning is subject to error, and since errors will usually degrade complex adaptive traits, most ‘pupils’ will not attain the level of expertise of their ‘teachers’. In this way, inaccurate learning creates a ‘treadmill’ of cultural loss, against which learners must constantly work to maintain the current level of expertise. This process is counteracted by the ability of individuals to learn selectively from expert practitioners, so that cumulative cultural adaptation happens when a rare pupil surpasses his/her teachers (Henrich 2004, 2006). Learners in larger populations have access to a larger pool of experts, making such improvements likely.”

Note from an earlier quotation by Henrich (2004) that imitation chains are not the only challenge associated

with social learning; another key aspect of this perspective is the question of *how* a highly skillful person is identified. Success and prestige are crucial solutions to this problem (Henrich and Gil-White 2001; Henrich and McElreath 2003). Thus, a key aspect of the cultural transmission model is its predictions about prestige. High levels of prestige is featured in most theoretical models of expertise. The cultural transmission model, however, characterizes it differently from the sexual selection models (honest signaling and mate provisioning) by conceptualizing it as a cue of competence. This is clearly stated, for example, in Henrich et al. (2001):

> "A substantial amount of cross-cultural ethnography (e.g., Dove 1993; Hammel 1964; Rogers 1995; Moore 1957) and laboratory psychology (for a summary, see Gil-White and Henrich 1999) suggests that humans everywhere possess a tendency to copy prestigious individuals, i.e., those who receive the most displays of respect/deference from others. This mechanism embodies two shortcut heuristics. First, by preferentially copying a"bundle" of cultural traits from prestigious individuals (prestige correlates with skill/knowledge and often wealth) copiers can rapidly acquire a repertoire of fitness-enhancing or success-oriented traits (i.e., better-than-average solutions to the problems of life). Second, rather than gradually learning via individual experience who the most successful, knowledgeable, or skillful individuals are, copiers rely on honest ethological and sociolinguistic signals of respect that other individuals display toward such high status individuals" (p. 345)

Predictions about prestige, particularly relating to its role in social learning (i.e., its relevance to the cultural transmission model) are conveniently summarized in Jiminez and Mesoudi (2019), and include: "skilled/knowledgeable individuals are prestigious"; "older individuals tend to be more prestigious than younger ones"; "generous individuals tend to be prestigious"; "knowledgeable/skillful/prestigious individuals receive freely conferred deference"; "people seek proximity to knowledgeable/skillful/prestigious individuals"; "people preferentially copy knowledgeable/skillful individuals"; and "prestigious individuals are influential/copied, even beyond their domain of expertise" (p. 3; see table 1 in the Jiminez and Mesoudi paper). Expanding on the rationale for including generosity with prestige, Jiminez and Mesoudi state:

> "Generosity has also been linked to prestige…in experiments (e.g., Flynn et al. 2006; Halevy et al. 2012; Hardy and Van Vugt, 2006; Willer, 2009) and ethnographic observations (e.g., Konečná and Urlacher, 2017; Price, 2003; Radcliffe-Brown, 1964). Because prestigious individuals tend to be both competent and generous (Cheng and Tracy, 2014; Cheng et al. 2010; Henrich, 2016), at least towards members of their ingroup, generosity can be used as a proxy for competence. According to [Henrich and Gil-White], this link is probably due to the fact that providing public goods is an excellent way to signal competence and, therefore, to receive further deference, which might be translated into fitness gains."

1.2 Proprietary knowledge model

The proprietary knowledge model focuses on the idea that know-how used by experts can allow them to provide extremely valuable services to other people. In contrast to the focus on cultural transmission in the foregoing section, this model proposes that experts' conceptual knowledge is restricted to specialists because it adds value to those specialists' services. Coded variables supporting and clearly distinguishing the proprietary knowledge model from others include expertise that is distributed among multiple experts; knowledge that is not widespread, or is rare, in a population; secretive and esoteric knowledge; assistance with uncommon and serious problems; evidence of successfully providing assistance; reputations for, and patronage based, efficacious services; and receiving payment for knowledge or services.

This model expands on existing ideas in the resource sharing and cooperation literature, where a person gains value from the services that s/he can provide that are difficult to replace.

Tooby and Cosmides (1996) argued that adaptations associated with reciprocal altruism are insufficient for dealing with uncommon and serious problems, such as health risks, because there are no profits to be gained by investing in someone unlikely to be able to reciprocate in the future. Thus, a better strategy would become "irreplaceable":

> "Consider X's choice between two potential objects of investment, Y and Z. Each helps X in

different ways; the magnitude of the benefits Z delivers are higher than the magnitude of the benefits that Y delivers, but the types of benefits that Y supplies can be supplied by no one else locally. Consider the alternative payoffs when one or the other enters a crisis and requires help. Extending 'credit' to a person in crisis may easily have a negative payoff if the *kind* of benefits that she customarily delivers could be easily supplied by others. ... A 'replaceable' person would have been extremely vulnerable to desertion. In contrast, extending credit has a higher payoff if the person who is currently in trouble customarily delivers types of benefits...that would be difficult to obtain in her absence. Selection should favour decision rules that cause X to exhibit loyalty to Y to the extent that Y is irreplaceably valuable to X. In other words, Y's associates will invest far more in rescuing her than they would if she lacked these unique distinguishing properties (Tooby & Cosmides 1984, 1989). Y may be helped, and Z abandoned even though the benefits Z delivers are greater" (p. 133).

Extrapolating from the Tooby and Cosmides paper and their own work, Sugiyama and Scalise Sugiyama (2003) note that Gurven (2000), in his "signaling generosity" model, shows that food exchanges among disabled individuals are largely driven by need. Sugiyama and Scalise Sugiyama go on to note, however, that these benefits can go beyond material resources to *services*, stating:

> "These include foraging and technological expertise, political savvy, medical aid, and alliance partnership in warfare (Sugiyama 1996; Sugiyama and Chacon 2000). Benefits that can be provided by one individual but not by others (or less well by others) are expected to be especially valued (Sugiyama and Chacon 2000; Tooby and Cosmides 1996). *Social niche specialization* (i.e., the cultivation of recognized, useful roles within the social group) is thus one hypothesized outcome of the selection pressure exerted by health risk, yielding interlacing networks of cooperative endeavors encompassing numerous benefit classes" (p. 169-170).

More broadly, services might straightforwardly maximize inclusive fitness (e.g., alarm calls among closely related social animals). For humans, these services – which are central to the proprietary knowledge model – are what give know-how its "market value". A key idea here is pseudoreciprocity, a cooperative strategy driven by byproduct benefits conferred by one's own self-interested actions (Connor 1986). This is likely to evolve in a wide range of species and domains, and Tooby and Cosmides (1996) apply it to friendship:

> "Behaviours that are not undertaken as intentional acts of altruism often have side-effects that are beneficial to others – what economists call positive externalities. Some potential associates exude more positive externalities than others. For a knowledge-generating and knowledge intensive species such as ours, such situations abound. Someone who is a better wayfinder, game locator, tool-maker, or who speaks neighbouring dialects is a better associate, independent of the intentional altruistic acts she might direct toward you. Similarly, there are an entire array of joint returns that come about through coordinated action, such as group hunting or joint problem-solving. Individuals may vary in their value as friends and associates because they contribute to the general success, or because their attributes mesh especially well with yours or with other members of your cooperative unit" (p. 137).

They go on to argue that if these scenarios were relevant for selection, then one should "be motivated to cultivate specialized skills, attributes, and habitual activities that increase their relative irreplaceability", and "preferentially seek, cultivate, or maintain social associations...where their package of valued attributes is most indispensible, because what they can offer is what others differentially lack" (p. 170).

Applied to know-how associated with a valuable service, "increasing their relative irreplaceability" is, by definition, guaranteed by possessing a service that is not widely disseminated in the population (i.e., cannot be replaced by someone else).

In some ways, the focus on individual cognition in this model might be seen as a counterargument against the cultural transmission model. Indeed, Pinker (2010) notes:

> "Given the undeniable practical advantages of reasoning, cooperation, and communication, it seems superfluous, when explaining the evolution of human mental mechanisms, to assign a primary role to macromutations, exaptation, runaway sexual selection, group selection, memetics,

> complexity theory, cultural evolution (other than what we call 'history'), or gene-culture coevolution (other than the commonplace that the products of an organisms's behavior are part of its selective environment)" (p. 8996).

It is not obvious how contradictory these positions ought to be, but this is a separate issue from our aim in this section, which is supplementing our motivation for the proprietary knowledge model. Nevertheless, because this quotation was from a publication eliciting the critical Boyd et al. (2011) reply (cited in the cultural transmission model in the section above), these models are clearly distinct.

1.3 Collaborative cognition model

The collaborative cognition model suggests that knowledge and expertise are a highly social and collaborative activity among multiple experts with complementary roles, insights, and areas of specialization. Variables supporting the social cognition model include distributed expertise across multiple types of specialist, each with narrow specialization; collaboration among experts to collectively produce more knowledge than each individual possesses; and knowledge ("know-how") shared among multiple experts.

Hutchins (1995) characterized the distributed cognition onboard a Naval ship in his seminal ethnography, which informs much of the later work that we operationalize in the social cognition model. His descriptions of the collaborative workings of the ship and its navigational capabilities describe an elaborate division of labor that is greater than its parts:

> "None of the component cognitive abilities has been amplified by the use of any of the tools. Rather, each tool presents the task to the user as a different sort of cognitive problem requiring a different set of cognitive abilities or a different organization of the same set of abilities" (p. 154).

Examples of this dynamic between social relationships and cognition as interdependent components of knowledge have been applied by cognitive of science researchers studying science and scientific concepts. Nersessian (2008) illustrates this in her book about this very subject, emphasizing that the questions scientists think to address ("problem situations") are, themselves, defined by the ongoing discourse defined by social context:

> "Novel concepts arise from attempts to solve specific problems, using the conceptual, analytical, and material resources provided by the cognitive-social-cultural context in which they are created. They are located within 'problem situations'. So, to understand creativity, it must be located not in the act but in these problem-solving processes' (p. ix).

More recently, similar views have been described by cognitive scientists (e.g., Sloman and Fernbach 2017) expanding on the notion that knowledge is a group activity, and that individuals are prone to varying levels of the so-called "illusion of explanatory depth". This idea, relevant to the social cognition model, is referenced in Keil (2003):

> "In the philosophy of science it has become evident that scientific explanations are often much shallower and less complete than they might seem to the outsider. … As outsiders we are often surprised at how interdependent scientists are on the expertise of others that have come before them and work elsewhere; but even with such dependencies, most individual scientists usually do know quite deep causal patterns in local domains" (p. 368).

In many ways, the social cognition model is similar to the cultural transmission model and should be viewed as a complementary to, rather than competing with, the cultural transmission model. However, we keep these two models distinct because the social cognition model makes no assumptions about the compulsory sharing of information. For example, Boyd et al. (2011, p. 10921) explicitly take issue with and diverge from the following attempt to incorporate focus on both cognitive capacities and cultural transmission by Barrett, Cosmides, & Tooby (2007):

> "Cognitive mechanisms underlying cultural transmission coevolved with improvisational intelligence, distributing the costs of the acquisition of nonrivalrous information over a much greater number of individuals, and allowing its cost to be amortized over a much greater number of advantageous events and generations. Unlike other species, cultural transmission in humans results in a ratchet-like accumulation of knowledge" (p. 244).

Similarly, Heintz (2013) cites Wimsatt and Griesemer (2007) while diverging only in the details from the cultural transmission model:

> "The cumulative aspect of cultural evolution has been used by proponents of dual inheritance theory to argue that transmission of cultural knowledge, rather than mere reaction of populations to the specifics of their habitats, was the factor that could account for cultural phenomena (Richerson and Boyd 2005). An image that easily comes to mind is one of piling up: during cultural evolution, humans pile up new ideas, traditions, and know-how. There is certainly some trust in this image. However, I would argue that Wimsatt and Griesemer's terms of 'scaffolding' and 'generative entrenchment' better grasp a central aspect of cumulative cultural evolution (Wimsatt and Griesemer 2007). The notion of 'generative entrenchment' suggests that faithful transmission is not a necessary characteristic of cumulative cultural evolution. Rather, old ideas are used in the generation of new ideas, and this is why they are stabilized. Old ideas are stabilized in time especially when they have become the basis of other widespread ideas and practices – they become entrenched" (p. 211).

Heintz goes on to expand on conceptual changes over developmental and generational timeframes, emphasizing the importance of distributed specialization:

> "[T]he distribution of cognitive labor enables people to become more knowledgeable in specific domains and ignorant in other domains that are covered by others. Economic exchange permits people to remain ignorant in certain domains and invest their cognitive resources in others. As a consequence, the group as a whole is more knowledgeable than when all members of the group know the same basic survival knowledge" (p. 211).

1.4 Honest signaling model

The honest signaling model emphasizes short-term mating, gaining relatively higher levels of access to multiple mates, and signaling fitness. The variables that most clearly support the honest signaling model, and distinguish it from the others, include competition among experts; prestigious or high status experts; public performances conducted by experts; costly displays by experts, either in their initiation rites, lifestyles, and/or rituals; experts who are described as highly charismatic, intelligent, and/or sexually attractive; and relatively high levels of mate access (e.g., multiple mates).

The honest signaling model is largely motivated by the idea that "human culture is mainly a set of adaptations for courtship" (Miller 1999, p. 72). On this view, expertise is a signal, the value of which creates a "within-species arms race that plays out within rather than across lifespans" (Winegard, Winegard, & Geary 2017, p. 45). Miller (2001) clearly outlines his perspective in a way that not only emphasizes competition for mates, but distinguishes it from the mate provisioning model that we also operationalized in this study:

> "In modern market economies people put a high value on wealth indicators during courtship ... David Buss has amassed a lot of evidence that human females across many cultures tend to prefer males who have high social status, good income, ambition, intelligence, and energy–contrary to the views of some cultural anthropologists, who assume that people vary capriciously in their sexual preferences across different cultures. He interpreted this as evidence that women evolved to prefer good providers who could support their families by acquiring and defending resources I respect his data enormously, but disagree with his interpretation.
> The traits women prefer are certainly correlated with male abilities to provide material benefits, but they are also correlated with heritable fitness. If the same traits can work both as fitness indicators and as wealth indicators, so much the better. The problem comes when we try to project wealth indicators back into a Pleistocene past when money did not exist, when status did not imply wealth, and when bands did not stay in one place long enough to defend piles of resources. Ancestral women may have preferred intelligent, energetic men for their ability to hunt more effectively and provide their children with more meat. But I would suggest it was much more important that intelligent men tended to produce intelligent, energetic children more likely to survive and reproduce, whether or not their father stayed around. In other words, I think

> evolutionary psychology has put too much emphasis on male resources instead of male fitness in explaining women's sexual preferences" (p. 210-211).

Although a highly sexually dimorphic runaway brain model is rejected in Miller (2001), his alternative mutual mate choice model nevertheless "emphasizes how sexual ornaments advertise each sex's fitness to the other sex – a function of mate choice that may stretch back to the origins of sexual reproduction itself" (p. 98). In other words, experts can be expected to use evidence of their creativity and intelligence to acquire mates, such that productivity "should increase rapidly after puberty, peak at young adulthood when sexual competition is greatest, and gradually decline over adult life as parenting eclipses courtship" (Miller 1999, p. 81).

Miller (2001) often uses competition among artists to illustrate his arguments. For example, he describes Hollywood as a failure "from a military point of view", going on to state: "Its avoidance of physical conflict allows it to amass, quietly and discreetly, enormous resources and expertise to produce ever more impressive shows" (p. 154). Here is another example in which Miller (2000) applies these ideas to competition among artists:

> "It evolved through sexual selection to serve the same courtship functions as almost all other examples of organic beauty and complex behavioral signals observable in nature. Such ornamentation often evolves as a reliable, costly indicator of the signaler's good health, good brain, and good genes. This leads to the further proposal that many design features of art function as indicators of the artist's virtuosity, creativity, intelligence, conscientiousness, and other important heritable mental and physical traits" (p. 25).

As demonstrated by these quotations, costly and ostentatious signals of ability, typically in a courtship context, are an important part of the honest signaling model. These courtship displays are "costly, conspicuous displays of cognitive prowess in language, music, art, and humor…[and] may be thought of as reflecting a person's overall genetic quality" (Geher, Camargo, & O'Rourke 2008). Winegard et al. (2017) favors, at least to some extent, the public and costly signaling aspects of the honest signaling model in helping explain the evolution of expertise:

> "We believe that expertise (or elite performance) often, but not always, functions as a costly signal of some desirable underlying trait (Miller 2001; Winegard, Winegard, & Geary 2014). Consider these features of expert performance that make it a good candidate for a costly signal: (1) expect performance is often broadcast publicly; (2) there are enormous individual differences in the domains in which people care about expert performance (music, sports, art), making for obvious rankings between competitors or performers; (3) performances are generally ritualized or organized in such a way that they can be assessed, also facilitating ranking of performers; and (4) expertise is difficult to achieve and quite rare, meaning it is costly and that it relies upon unique constellations of underlying traits and large amounts of leisure time. These traits may consist of, but are not limited to, conscientiousness, athleticism, intelligence, the size of one's social network, and ambition (Hawkes & Bliege Bird 2002; McAndrew 2002; Miller 2001)" (p. 44-45).

Their citation of Hawkes and Bliege Bird (2002) is in reference to a discussion of the "show-off hypothesis" of male hunting, i.e., the idea that hunting skill is a sexually selected signal of ability. Hawkes and Bliege Bird (2002) argue that costly signals can range in the benefits that they provide, shortly after citing Veblen (1899) on conspicuous consumption and Zahavi (1975) on handicapped signaling:

> "Costly signals are enormously variable. One important dimension of variation is between signals that provide little but information and those that provide benefits to the audience in addition to information. For example, when a display consists of providing feasts, others gain from participating in the feast. By signaling in this way, the show-off provides something besides information about a hidden quality to the audience" (p. 58).

With respect to skills and their displays, Hawkes and Bliege Bird (2002) go on to emphasize "broadcast effectiveness", i.e., that "signals must be detected effectively by appropriate recipients". They then argue that:

"Signals designed to acquire or maintain higher social standing in a group should be directed to the group at large; other more specialized signals may be directed to smaller subsets of the population. Signalers competing for popular prestige should seek to gain a larger and larger share of the advertising market. They gain a larger share by providing more of what the viewers want to see or consume than the competition provides. The provisioning of collective goods may serve the purpose of reaching a wide audience better. Both competition among signalers and audience preference for particular signals can play a role in shaping the display" (p. 65).

1.5 Mate provisioning model

The mate provisioning model is based on the idea that high standing based on skill is sexually selected, but in contrast to the honest signaling model, it emphasizes long-term mating strategies, parental investment, and a preference for resource access. The variables that support and most clearly distinguish the mate provisioning model are prestigious or high status experts; reputations for generosity, commitment to parenting, and mate fidelity; and instances of mate provisioning and/or investment in offspring.

A clear statement of the mate provisioning model is outlined in summary after a discussion of his arguments in previous works, and similar works of others on prestige and parental investment, in which Barkow (1992, p. 635) states:

"Thus, I have in effect hypothesized that (a) human beings tend to strive for higher relative standing and this striving usually takes the form of seeking control over surplus production or over the means of production; (b) human beings everywhere tend to be nepotistic; (c) the view of social exchange algorithms presented by Cosmides and Tooby (1989, this volume) is essentially correct; and (d) both cross-culturally and historically, surplus production is associated with differences in social rank in all cases, and with social stratification in most. If any of these four hypotheses is inaccurate, then the entire argument must fall."

As suggested in the honest signaling model, the mate provisioning model is more consistent with human mating in the sense of Buss (1989), which he characterizes based on his reading of Trivers (1972):

"Males may provide mates with food, find or defend territories, defend the female against aggressors, and feed and protect the young. Human males may also provide opportunities for learning, they may transfer status, power, or resources, and they may aid their offspring in forming reciprocal alliances. These forms of male investment, when provided, tend to decrease the investment disparities between males and females. … In species with male parental investment, such as *Homo sapiens* (Alexander & Noonan 1979), females should seek to mate with males who have the ability and willingness to provide resources related to parental investment such as food, shelter, territory, and protection. … The hypothesis that females will mate preferentially with males bearing greater gifts, holding better territories, or displaying higher rank has been confirmed empirically in many nonhuman species (Calder 1976; Lack 1940; Trivers 1985; see also Betzig et al. 1988)" (p. 2).

This view of human mating is also summarized in Buss (1992):

"The female tendency to favor high-status males is only one part of the constellation of evaluative mechanisms expected to underlie mate choice in women. Selection should also have favored mechanisms in females designed to detect and prefer males who were willing to convert status and ability into paternal assistance. … All else equal, therefore women should find men who demonstrate the willingness to devote time and resources to a chosen female and her offspring more attractive than men who do not" (p. 272).

The mate provisioning model therefore emphasizes human mating as a long-term investment strategy rather than a short-term one, a framing explicitly used by Schmitt (2010). He concludes that "among humans, many men invest heavily in their children, teaching them social skills, emotionally nurturing them, and investing both resources and prestige in them" (p. 57). The rationale for a strategy in which individuals seek status, but maintain high mate provisioning plus parental investment, is clarified from an evolutionary perspective by Gavrilets (2012) in his model of pairbonding in human evolution:

"Top-ranked males can easily beat out or chase away the low-ranked males and steal the paternity, making the investment of low-ranked males in production wasteful. However, after females start developing preference for being provisioned, the low-ranked males' investments start to pay off. In the model presented here, male provisioning and female faithfulness coevolve in a self-reinforcing manner. At the end, except for a very small proportion of the top-ranked individuals, males invest exclusively in provisioning females who have evolved very high fidelity to their mates" (p. 4-5).

Note from the foregoing quotation that mate fidelity and self-imposed "reproductive ceilings" are not a limitation, but a feature of the mate provisioning model. This is also explicitly featured in Stewart-Williams and Thomas (2013):

"As brain size increased in the hominin lineage, our young became progressively more dependent and the childhood period became progressively longer. As a result, pair bonding and male parental care became central elements in our reproductive repertoire. This dramatically reduced the discrepancy in the maximum number of offspring that men versus women could produce. Although in principle a man could impregnate hundreds of women every year, in practice the reproductive ceiling for even the most attractive men was almost always much lower" (p. 138).

2 Description of our search query

We collected text records from the electronic Human Relations Area Files World Cultures database while intentionally targeting those that reflect some kind of "[ethno]scientific expertise" in the ethnographic literature. Inclusion was therefore based on descriptions of expertise in a potentially relevant conceptual knowledge domains, which we describe in the main text. Our exact search query, i.e., the set of OCM codes and search terms that generated our initial results, was:

((Cultures = (Any Culture)) AND (((Subjects = ('173' OR '278' OR '571' OR '577' OR '578' OR '581' OR '582' OR '583' OR '584' OR '751' OR '752' OR '753' OR '757' OR '758' OR '759' OR '761' OR '802' OR '803' OR '804' OR '805' OR '810' OR '811' OR '812' OR '813' OR '814' OR '815' OR '816' OR '820' OR '821' OR '822' OR '823' OR '824' OR '825' OR '826' OR '827' OR '828' OR '829' OR '830' OR '831' OR '832' OR '833' OR '834' OR '835' OR '836' OR '837' OR '838' OR '839' OR '840' OR '841' OR '842' OR '843' OR '844' OR '845' OR '846' OR '847' OR '848' OR '860' OR '861' OR '862' OR '863' OR '864' OR '865' OR '866' OR '867' OR '868' OR '869' OR '875' OR '877')) AND (Text = (knowledgeable OR expert* OR proficient* OR skilled OR specialist* OR practitioner*)))))))

The initial results from the entire eHRAF database found 5734 paragraphs in 1901 documents in 311 cultures, and filtering Probability Sample Files (PSF) found 1595 paragraphs in 483 documents in 60 cultures. ADL read, contextualized, and filtered these remaining 1595 paragraphs to determine whether or not they were appropriate examples of ethnoscientific expertise, i.e., requiring high levels of culturally specific conceptual knowledge about the social and/or natural world. Text records that were included consisted of the focal paragraph from the results, and a contiguous set of paragraphs that provided context necessary for understanding the paragraph that turned up in the results.

2.1 Inclusion criteria

The *a priori* justification for including text records was based on whether or not some kind of "[ethno]scientific" expertise was described. We defined scientific domains in this context as conceptual domains where knowledge is ultimately acquired though observation, testing, and/or learning about natural and/or social principles, which in turn result in developing theories, either over the life course or via cumulative culture, that could potentially be usefully applied to a number of possible types of tasks or applications (e.g., crafts, healing, hunting, trapping, conflict resolution, or ethical quandaries). These applications are referred to and distinguished as "products" in our main text, and their underlying conceptual theories are similarly referred to as "know-how". Crucially, we were searching for knowledge that is not specific to any particular use, but uses might recur because they are relevant to certain types of knowledge. For example, it is unsurprising that plant knowledge (ethnobotany) is frequently relevant to

medicine, despite its potential uses in basket weaving. Similarly, animal knowledge (ethnozoology) is useful to hunting and trapping, but might not be limited to these activities alone. In other words, knowledge might be "for something", but what that "something" is not the topic being queried. Rather, it is high levels of investment in the underlying knowledge (expertise) that are our outcomes of interest.

3 Complete description of our coding scheme

Each text record was coded by two independent coders (ADL and CH) for each variable in our coding scheme, as described in the main text. Note that some variables in our dataset are evidence against variables in our coding scheme.

Categorical variables are indicated in the descriptions, and were coded as presence/absence for each category as a separate column/variable in our data matrix. This meant that multiple categories could be present in a single text record. For example, if male and female experts were both present in a text record, then we coded presence for both males and females.

Here are our coded variables and a brief description of each:

Text ID: Provides a unique key id number corresponding to a text record, citation, and OCM codes.

Case/model: Does the text record describe a specific case about an expert, a cultural model of expertise, or both? (*Categorical:* Case, model, both)

Age: How old are the experts described? (*Categorical:* Child/adolescent, Adult, Older adult/elderly)

Sex: What is the sex of the experts described? (*Categorical:* Male, female, either/both present)

Costly lifestyle: Does being an expert entail some kind of costly lifestyle, where the expert must fulfill certain obligations on a regular basis, making his/her life substantially more difficult than it would be otherwise?

Costly ritual: When applying his/her expertise (e.g., to perform a service, teaching), does the expert perform some kind of ritual (can be either public or private) in which s/he takes on some kind of cost, e.g., in the form of fitness, money, resources, pain, or risk?

Costly initiation: Does the expert perform some kind of initiation rite, in which s/he takes on some kind of cost (e.g., in the form of fitness, money, resources, pain, or risk) as a condition of being considered an expert by other people?

Expert teaches others: Do experts teach other people what they know? Does becoming an expert entail some kind of apprenticeship, mentorship, assistantship, or knowledge sharing, for example? The expert is described sharing his "know-how" (knowledge or skills) with others, which might or might not be exchanged for something else.

Expert purchases knowledge: Experts conferred a benefit to another expert in exchange for acquiring their knowledge/skill from another person.

Public performance: The expert demonstrates his/her abilities in a public setting, perhaps involving some kind of performance that is visible to others.

Private performance: The expert demonstrates his/her abilities or provides services to others in a private setting.

Experts compete: If multiple experts exist, then they are described as having a competitive relationship with each other.

Experts collaborate: If multiple experts exist, then they are described collaborating or as having a collaborative relationship with each other.

Distributed knowledge among multiple experts: If multiple experts exist, then their expertise is distributed across different roles or types of expertise, which do not strongly overlap with each other.

Hierachy within the domain of expertise: If multiple experts exist, then their expertise involves a hierarchical structure *among the experts* within a given domain, either in seniority among experts or level of skill. (This is not a reference to prestige in the broader community; it is strictly within-domain structure among experts, e.g., senior vs. novice specialists.)

Sexually attractive: The expert is described as being sexually attractive.

Charismatic: The expert is described as being charismatic.

Intelligent: The expert is described as, or has a reputation for being, intelligent.

Multiple mates: The expert has multiple mates.

Deference: People (non-experts or novices) are described as deferring to the expert by displaying signs of respect. (For example, this might include deferring to someone by paying them a respectful greeting, but would *not* include people simply deferring to a medical expert for medical advice.)

Prestige: The expert is considered prestigious, high status, and/or is well-respected in the community. This might include people who are generally paid more attention than others, but is distinct from reputation for efficacy (see below).

Others seek proximity to expert: Is the expert a preferred social partner (e.g., friend, coworker), or frequently/preferentially sought out for advice? Do people (non-experts) seek proximity to the expert, including for reasons that are not directly to the benefits or traditions relating directly to their domain of expertise?

Generosity: Is the expert generous, or does s/he have a reputation for being generous?

Mate fidelity: Does the expert have a mate who gives him/her exclusive sexual access? (Note: this one mostly comes up when violations/evidence against arise. It can include involuntary examples, such as cheating on partners, and voluntary ones, such as wife sharing among experts.)

Reputation as good parent: The expert has a reputation for being a good parent.

Parental investment: The expert is described conferring some kind of benefit to his/her offspring.

Provisions mate: The expert is described conferring some kind of benefit to his/her mate.

Narrow specialization: An expert or experts is/are described as having a narrowly specialized knowledge in their domain of expertise.

Broad generalist: An expert or experts is/are described as having a broad range of general knowledge, such as multiple domains of expertise (e.g., medicine, geography, and meteorology) or multiple unrelated specialties in a domain of expertise (herbal medicine, bone-setting, and childbirth).

Influential outside of area of expertise: The expert has influence over other people in domains outside of his/her domain of expertise. (Examples might include political influence, if the expert's domain is not related to politics, or trusted with medical advice if the expert's domain is not related to medicine.)

Evidence success: Evidence is documented by the ethnographer or his informant describing the expert as applying his/her knowledge or skill for a patron, and successfully bringing about an intended and/or desired outcome.

Secretive knowledge: An expert or experts is/are secretive about their knowledge or skills ("know-how"), and attempt to keep it hidden from (e.g., uninitiated) non-experts.

Assists with an uncommon and serious problem: Does the expert have patronage or clientele for their applied knowledge and/or services based on an uncommon and serious problem that arises for the patron (which the expert might be able to help with)?

Receives payment: Does the expert receive money or other material resources (e.g., gift or payment) for his/her applied knowledge and/or services?

Access to mates: Does the expert receive mates or sexual favors for his/her applied knowledge and/or services?

Patronage based on efficacy: Does the expert have patronage or a clientele for their applied knowledge and/or services based on an apparent expectation that the expert will bring about desired and/or beneficial outcomes for the patron? (Instead of costs, familiarity, or tradition, for example.)

Cares about reputation: The expert cares about his/her reputation and takes measures to present himself/herself in a positive and self-serving way.

Reputation for efficacy: The expert has a reputation for applying his/her knowledge in a way that can bring about desirable results for other people.

Possesses widely distributed knowledge: While the expert is highly knowledgeable in his/her domain (by definition), other "non-experts" in the community also tend to be fairly knowledgeable in the same domain.

Ornamentation: The expert wears some kind of ornamentation or body alteration that symbolizes to others his/her special status as an expert.

4 Examples of our coded variables

In this section, we include examples of text that would be coded as supportive for each variable in this study. Note that some of these might be multiply coded, i.e., some text records would also constitute evidence for additional variables, in addition to the variables for which they are illustrative examples.

Possesses secretive knowledge

> The secret language follows the grammatical patterns of standard Trukese, but it is laced with cryptic distortions. Secret words, altered regular words, regular words with special meanings, and esoteric metaphors make this language incomprehensible to those who speak only standard Trukese. Masters of strategy use this language among themselves and in speeches at formal meetings. It is also the medium in which they preserve "significant history" (wuruwo), that is, history that justifies claims to valuables.

Parental investment

> Knowledge of this kind is freely applied to the curing of a lineage mate or other close relative as a personal favor. Any one else, however, must pay in advance. The patient brings goods to the specialist and requests his aid. When cured, he is further obligated to the practioner and makes him a present. Such knowledge, then, not only has practical social value, but through its application can provide other forms of wealth for the specialist. To this knowledge, too, the specialist's children and lineage mates have a claim. Without their permission, he may not teach it to another unless he receives compensation for it. For if the specialist teaches someone else, he dissipates the monopoly his heirs will someday enjoy and lowers their future earning power.

Costly initiation

> Offers of help soon give way to competition as the established students come to regard him as a rival for the master's personal instruction and encouragement. To be the master's favorite means rapid advancement through the curriculum and a seat at his table. Without his patronship, a student is committed to years of physical misery, scraping to meet his subsistence needs, and paying his "tuition" in arduous and humbling ways, by gathering wood for the master's fire, tilling his garden, and washing the feet of the master and his visitors.

Assists with uncommon/serious problem

> The ng'aka, therefore, is employed where a malady or misfortune is caused by something mysterious and inexplicable to ordinary people. He is consulted only on the advice of a diviner though...he may himself be the diviner.

Hierarchy w/in domain of expertise

Most adult men know something about the curing of minor ailments, but very few know how to cure serious illness. There is a graded series of curing techniques and only the most widely known shamans know all of them. Shamans are thus ranked according to their knowledge and abilities. Their powers are founded upon their knowledge of myths. Most adult men know a considerable number of myths but shamans differ from the rest in two respects: first, they know more myths, and secondly, they know and understand the esoteric meaning behind them.

Assists with a common problem

Wood for building purposes is felled when one comes across a suitable tree in the forest. … Carpenters keep wood stored at home. Some men are so interested in woodwork that they cannot make any conversation except about wood. An old man in Utsjoki called Tor'te-t seähtsi ("Uncle Spinning Wheel") had gathered so much wood in his home that he could hardly find room enough to sleep in. Boats and sleds require the longest time to prepare, but making a spinning wheel requires the greatest skill. Specialists in making spinning wheels have been known in Utsjoki since at least the middle of the previous century.

Broad generalist

A few people are experts in several different forms of special knowledge; some know one kind, and some know only fragments of one kind. Although everyone knows that many kinds of special knowledge exists, any particular type is important because few others share it.

Charismatic

Another reason why the Toradja are set on the treatment with kajoe sina by a Mohammedan is because he combines with his work all sorts of mysterious things, which are impressive. With great ostentation the medical expert pronounces a magic formula over the pot, and if the sick person recovers, then he ascribes this more to the power of this formula than to the effect of the medicine.

Reputation for efficacy

When a somewhat more complicated disease develops, one has recourse to the known herbal specialists. These specialists…have become known for their general skill or perhaps for success in treating special diseases.

Receives payment

The patient pays the doctor a fee, usually tobacco and some common article, but one person charges a fee of $5.00 per treatment.

Knowledge domain is not widespread

There are types of special knowledge relating to medicine and curing, house and canoe construction, navigation, sorcery, divination, and fighting. In general, only older people have special knowledge. … Not everyone in the older generations possesses special knowledge. It is carefully guarded from outsiders and can only be learned from a close kinsman, or purchased at considerable expense from a non-kinsmen.

Deference

There are types of special knowledge relating to medicine and curing, house and canoe construction, navigation, sorcery, divination, and fighting. In general, only older people have special knowledge. … Not everyone in the older generations possesses special knowledge. It is carefully guarded from outsiders and can only be learned from a close kinsman, or purchased at considerable expense from a non-kinsmen.

Knowledge domain is widespread

Laymen have little difficulty in selecting an appropriate kind of wogeysa , since the ailments treated by each category of wogeysa are characterized by unambiguous syndromes which are easily identified by laymen.

Costly lifestyle

The agamiy exposes himself to certain dangers in the performance of this therapy. Because he must remove a pathogenic substance through the medium of the sickman's blood, there is the danger that, should any of the blood enter his own mouth, the agamiy may himself fall ill with the ailment he is treating. In addition, contact with this blood threatens those chronic ailments from which the sickman suffers (such as the rheumatoid pains associated with kitin, syphilis).

Public performances

The wabeno was a highly honoured public official, in a sense, for he was the servant of his community, even though he was entitled to charge a fee for his services. Since his prestige depended partly on his popularity he generally gave a public feast and dance whenever a patient paid for the remedy that dispelled his sickness, or a hunter offered fitting compensation for the medicine that had delivered the game into his hands.

Costly ritual

At intervals in the dances one of the medicine-men might drum and dance alone around the fire, then, stooping, pick up from the embers a hot stone; or he might dip his fingers into a boiling cauldron, extract a piece of meat, and swallow it without evidence of pain. Some Indians say that he derived these powers from the medicines he rubbed on his hands, and that although he neither ate nor drank for four days and four nights previously, his strength remained totally unimpaired.

Knowledge distributed/multiple experts

Knowledge of magic and privileged access to spirits is distributed among four categories of people: herbalists, ecclesiastics, shamans, and debtera. Most herbalists incorporate some magic into their recipes. The rural herbalist is usually a farmer, indistinguishable from his fellows except for his curing. He is known to his public by his ability to treat a specific, named ailment, and his professional title indicates only his disease name specialty and his degree of proficiency, e.g., "expert curer for body ulcers." Few herbalists offer therapies for more than three or four ailments.

Influential outside of area of expertise

Throughout these years, the intellectuals, as literati associated with the court (and often the court itself: some of Thailand's greatest poets and dramatists were kings) helped maintain, develop, and glorify this civilization, but not change it radically.

Evidence of success

Besides the priestess, in each village there are several men and women about whom it is known that for certain indispositions they are familiar with a remedy that has already been applied with success many times. Such a person is then asked to come to try his skill on the sick person.

Experts collaborate

Every tagañ owns a recipe for an unguent, and the medicaments used in it are kept a professional secret. However, not all tagañs have recipes for kusil prophylaxis. Without the latter, a tagañ who wishes to treat compound fractures must obtain the services of an herbalist whose specialty is the treatment of kusil.

Experts compete

In their social role, midwives felt personal but not group solidarity. A solid bond existed between an older midwife and the particular younger one to whom he or she had given training and magic. ... The expert who brought mother and baby through before the deposed midwives glowed inwardly with pride and skill and satisfaction that the magic of his particular teacher had proved

effective. The sharpness of the competition reverberated through the words, "I pushed the other midwives down." Community standing or "face" was not lost by an unsuccessful case because of the midwives' fundamental role as helpers only, their faith in their own experiences, and again, the conviction that the course of childbirth was merit-determined.

Reputation for generosity

These first abinet [esoteric skill] are generally gifts from student friends … Among these companions favors are expected to be freely given and no formal accounting is made. Beyond this circle all students are implicitly rivals (towdaderiy). Another source of abinet at this time is some older, more knowledgeable student. Here, the relationship between donor and recipient is framed as an exchange between a patron (radat) and his client (taraj), a customary dyad contrasting power with dependency, and generosity with deference. The new boy appeals that he is abject and threatened by mortal dangers; the donor, by his gift, publicly asserts a status difference which sets him off from novices in general.

Intelligent

Certain natives, served by a good memory and a livelier intelligence, took an interest in these traditions and legends to the extent of learning those of neighboring families and even of neighboring kingdoms and little by little acquired a reputation as specialists in these matters.

Multiple mates

First, by being a powerful ritual specialist, Roberto had political influence and social prestige in society. Therefore, he could claim a woman in exchange for his ritual services. Secondly, he knew well that Eulalia was the only child of an old, widowed and remarried man in the headwaters area of the Goya stream … Alberto, who indeed cared for Eulalia as for a proper sister, was indebted to Roberto and dependent upon his ritual services. So they could do nothing but accept the marriage. In this way, Roberto took Eulalia as his second wife and became one of the few polygynous men in the territory.

Narrow specialization

Aymara medicine is highly specialízed, and consequently has a great many different categories of practitioners. … It is probably safe to say that no other primitive group known to modern ethnology has such a rich specialization among practitioners of native medicine.

Ornamentation

As soon as possible, the patient's family presents the principal specialist with a string of multicolored glass beads, which he places around his neck for the ceremony to follow … The multicolored bead necklaces are frequently worn by snake medicine men even when they are not working on a case, and serve as identity badges.

Prestige

In fishing, especially deep-sea fishing, and in some other crafts, knowledge of the technical processes and of the associated ritual is unequally distributed. … One effect of the possession of such knowledge lacking in others is the opportunity of enlarging one's resources. This is done by resort to fishing banks not commonly known, or use of a special technique at times when the fish refuse all others; or, in the case of a craftsman, by more prompt replacement of his implements, or by gaining goods in payment for his work for others; or to a small extent by the securing of a material return for the imparting of his knowledge to others. Important as an inducement to the acquisition of such personal knowledge, however, is the prestige that it obtains.

Private performances

There is no particular moment that is better than any other for informal conversation about genealogies, except that people are most likely to want to discuss genealogy when they are contemplating a marriage, considering litigation that involves genealogical claims, or asking for

financial assistance or political support on grounds of kinship. The conversation usually occurs inside the hut or room of the person whose genealogy is being discussed. The occasion is quite private, and other griots are not likely to attend.

Provisions mate

When, as a result of a different political structure, Ogobara was no longer "canton chief", the village secretary was obliged to look for other occupations. He went to Bandiagara and in the hospital there began a course in male nursing. He lives there in the family house and provides for his two wives and his children who have stayed with Ogobara's family in Sanga.

Mate access for expertise

Most of the bridewealth and other marriage expenses are waived and the girl is given to the husband by those having authority over her marriage dispensation rights … A man may wish to express his gratitude to a mallam for supernatural aid having to do with medicine, prayers, or divination used by the practitioner for the benefit of the group bestowing the girl. In the same way, most individuals having power to bestow a girl would hesitate before refusing the request of a mallam because of the general awe in which he and his supernatural powers are held in the public eye.

Wealthy

The ideal type [of specialist] is a person of respectfulness, bravery, and strong thought who also controls abundant valuables.

Sexually attractive

Some experts may use the DixBo ne [ritual object] on their own account, in which case they become irresistible to all women in the village. Informant mentioned the name of an individual who was an exceedingly successful love expert on his own account.

Others seek proximity to expert

If the professor wants to discourse on the domain of a particular spirit residing in the jungle, he will do so, and on his own time. If he wants to collect medicines one day, the student may follow along. And if he decides to pass on some esoteric information about the plants being gathered – or if he decides not to – that is the way things will happen.

Expert teaches others

When a young Kuna becomes a disciple of an experienced curing specialist he places himself in a thoroughly submissive position with regard to his teacher. Once the branch of knowledge he wishes to learn has been decided upon and fixed, the specialist controls the manner in which instruction proceeds, the sequence in which all knowledge is passed out, and the topics to be discussed during each session … When learning proper begins, the student characteristically listens attentively to a short portion of a chant several times, then is told to repeat it. When he has mastered this task adequately, another piece of the chant is given to him in the same fashion, and he repeats it. On and on it goes until the entire chant has been learned. As the student progresses and demonstrates his capacity for learning he is given information or symbols and their underlying meanings, commentaries on the nature of curing and disease in general, and instruction in accompanying ritual. Such information is conveyed to the student either by demonstration or verbally.

5 Coding our domains into domain types

Text records were categorized on a large number of knowledge and skill domains, as shown in network of coded domains in the main text. We assigned most of these domains, particularly those which were sufficiently informative to allow us to adhere to our assignment criteria (see below), to a specific domain type.

Conceptual domains types ($N = 322$, 58.9% of text records; referred to as conceptual domains, henceforth) were largely designated by the OCM codes in the eHRAF referred to as "ethnoscience", described as "ideas about nature and people". Some conceptual domains were frequently associated with these designated codes, and were agreed upon by both coders (ADL and CH) based on their tendency to involve mostly private and mental processes remaining opaque to others. Conceptual domains included ethnobotany, ethnopsychology, ethnometeorology, ethnozoology, traditional history, astronomy, genealogy, life and death, ethnogeography, ethnoanatomy, mathematics/measures, physiology, philosophy, ethnocosmology, ethnophysics, divination and uncertainty, interpreting misfortune, and literature.

Motor skill-related domain types ($N = 156$, 28.5% of text records; referred to as motor domains, henceforth) were agreed upon by both coders (ADL and CH). The general criteria for this domain type was that it necessarily involved some kind of behavior (i.e., motor activity) that was observable to other people, and was relatively transparent compared to primarily private and mental processes, such as plant knowledge or medical diagnoses. Motor domains included subsistence, art/crafts, boat making, body alteration, woodworking, food preparation, construction, navigation, warfare, music, dancing, fighting, dentistry, and injury.

Medicine ($N = 341$, 62.3% of text records) was its own large and overarching domain type, which linked to many conceptual domains (shown in the domain network, in the main text). In general, medicine was more often linked to conceptual domains than motor domains (57.8% vs. 47.4%, respectively), but the difference between these associations was not exceptionally large because conceptual domains were often co-present with motor domains (58.3% of conceptual cases included motor domains). Further, injury was highly linked to medicine but routinely involved motor skills, such as massage and bone-setting.

In sum, each *domain* was assigned to a unique *domain type*: conceptual, motor, or medicine. However, because each text record typically described (and was coded on) multiple domains, each text record often had multiple domain types. For example, if a medical specialist was described as using private, conceptual plant knowledge to prepare a medicine, then this text record would be coded on "medicine" and "ethnobotany" domains, and assigned "medicine" and "conceptual" domain types. If a hunter was described as using animal knowledge to improve his prospects on a hunting expedition, then this text record would be coded on "ethnozoology" and "subsistence" domains, and "conceptual" and "motor" domain types. See figures S1 through S3 for agreement plots indicating the proportion of overlap among each pair of domain types.

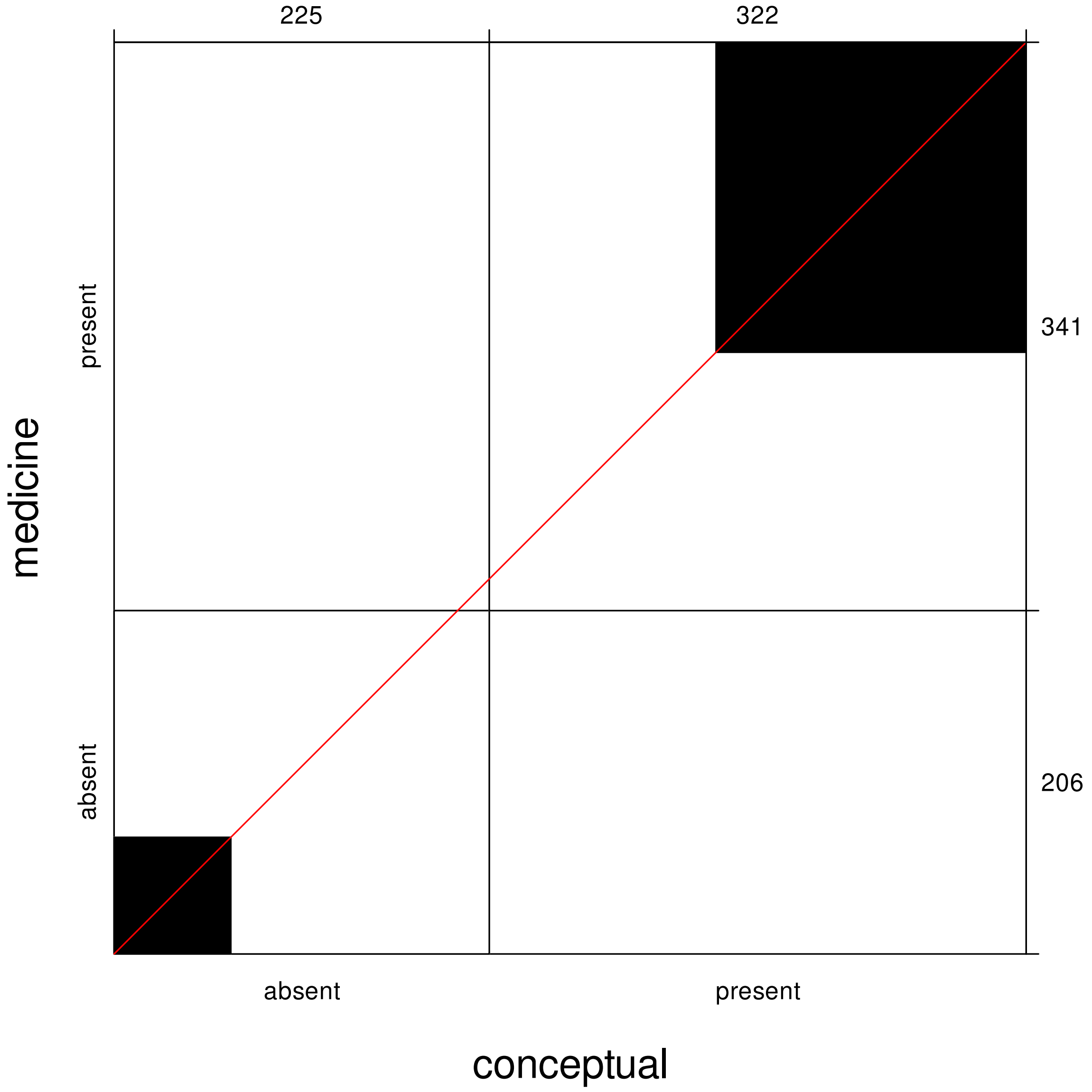

Figure S1: Agreement plot showing the proportions of presence/absence of conceptual and medicinal domain types, and their levels of overlap, among each of our text records. The dark spaces represent the proportion of overlap (agreement) for the presence and absence these domain types.

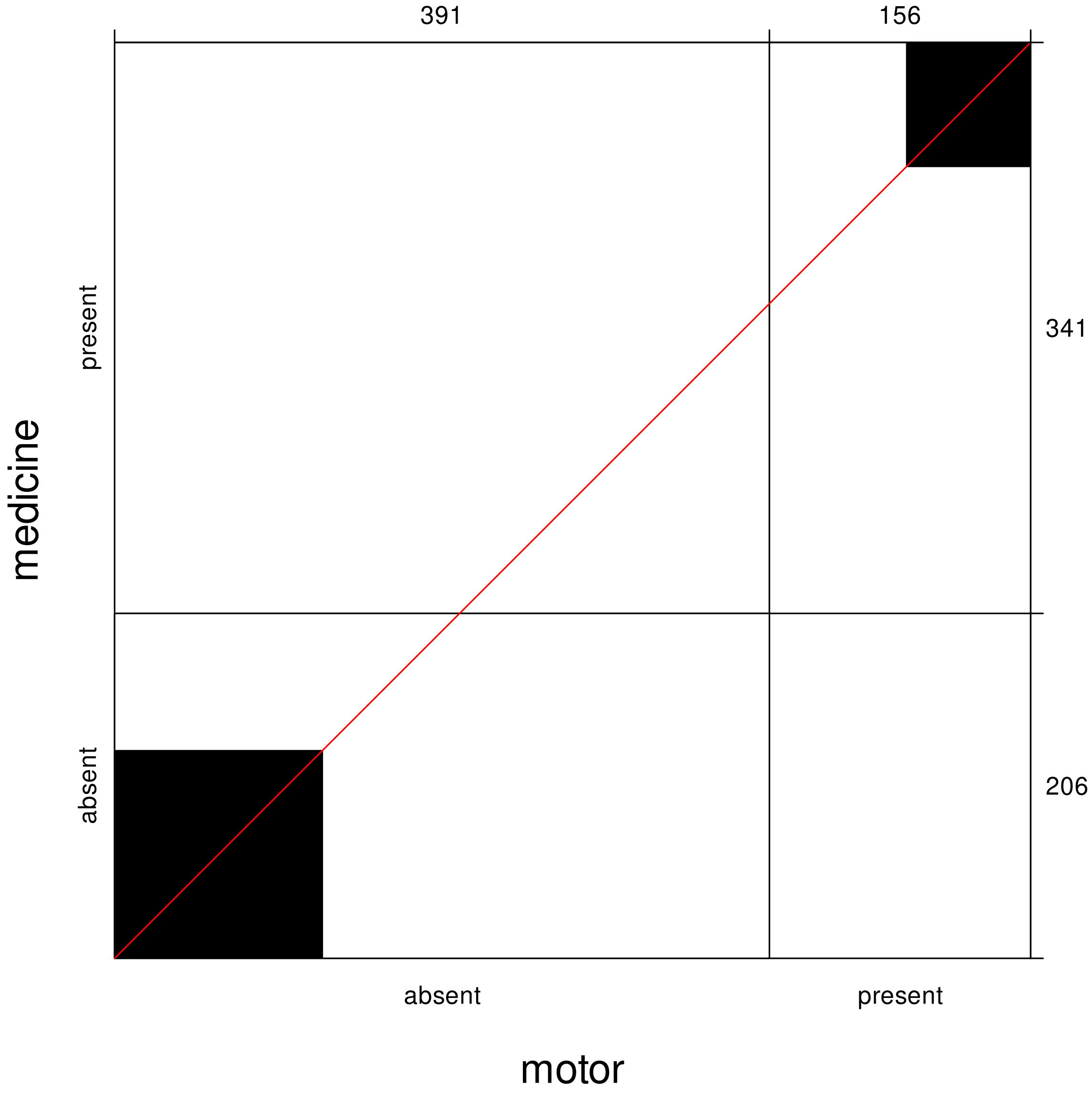

Figure S2: Agreement plot showing the proportions of presence/absence of motor and medicinal domain types, and their levels of overlap, among each of our text records. The dark spaces represent the proportion of overlap (agreement) for the presence and absence these domain types.

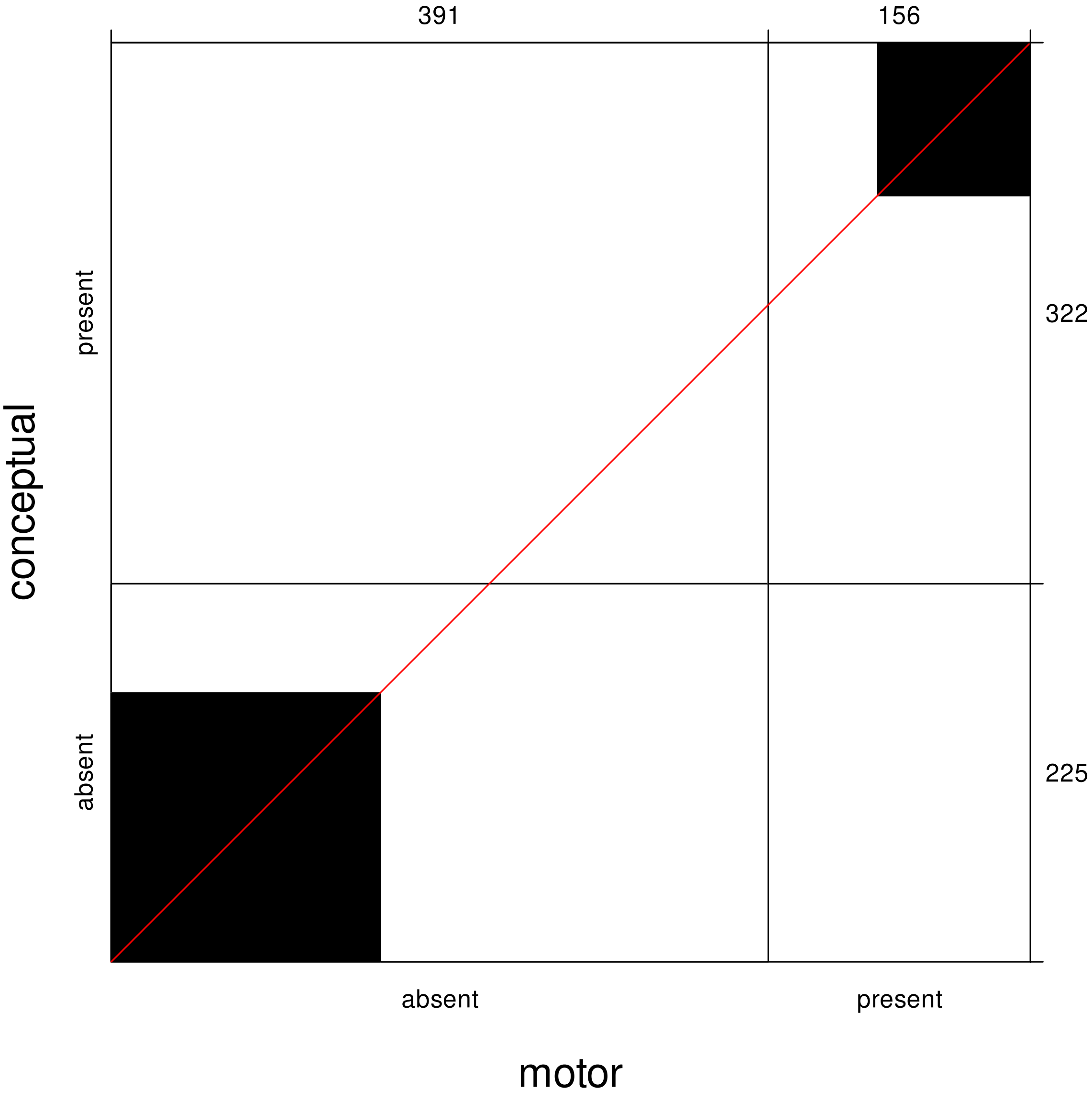

Figure S3: Agreement plot showing the proportions of presence/absence of conceptual and motor domain types, and their levels of overlap, among each of our text records. The dark spaces represent the proportion of overlap (agreement) for the presence and absence these domain types.

6 Interrater reliability

Our dataset was coded independently, using the coding scheme described above, by the first author (AL) and second author (CH) on this study. After the independent coding phase, we checked the interrater reliability and reported these as percentage agreement in our data matrix and Cohen's kappa in the main text. An agreement plot visualizes our level of agreement, prior to reconciling the differences in our codes (figure S4). After the independent coding and interrater reliability analysis, ADL and CH went through each disagreement of each text record and agreed on how they should be reconciled.

7 Explanation of the elasticnet regression model

Here, we briefly describe the elasticnet regression model. Standard regression models are fit by minimizing an objective function. In ordinary least squares regression the objective function is the residual sum of squares (RSS), and in logistic regression it is the negative log-likelihood, $-loglik(\beta)$. Penalized regression models instead minimizes the objective function plus a penalty term based on the magnitude of the coefficient vector [@le_cessie1992ridge]. For linear regression this is:

$$\frac{1}{2}RSS/n + \lambda * penalty$$

and for logistic regression:

$$-loglik(\beta)/n + \lambda * penalty$$

There are two popular forms of penalized regression: ridge regression and lasso regression. For ridge regression the penalty is $||\beta||_2^2 = \sum_{j=1}^{p} \beta_j^2$, where the β_j are the regression coefficients, and for lasso regression the penalty is $||\beta||_1 = \sum_{j=1}^{p} |\beta_j|$. When $\lambda = 0$, this reduces to the standard estimation. As $\lambda \to \infty$, the coefficients β_j are "shrunk" to 0. Thus, when λ is small, the βs are relatively unrestricted, which can result in a good fit to the current sample (low bias), but a poor fit on future samples (high variance); roughly, the model will tend to be over-fitted. When λ is large, the βs tend to shrink toward 0, which reduces fit on the current sample (high bias), but results in a more stable fit across samples (low variance); roughly, the model will tend to be under-fitted. The optimal value of λ is typically found by minimizing cross-validation error.

With the lasso penalty, some coefficients might be set to 0, i.e., dropped from the model, which aids interpretation, but when variables are correlated, the lasso might drop some that are genuinely related to the outcome. In ridge regression, in contrast, the coefficients of correlated variables are shrunk to similar values; although the coefficients of some predictors might be very small, all predictors are retained in the model, which can make interpretation difficult.

Elastic net regression combines the advantages of ridge and lasso penalties using an additional tuning parameter α, $0 \leq \alpha \leq 1$:

$$penalty = (1-\alpha)/2||\beta||_2^2 + \alpha||\beta||_1.$$

Thus, $\alpha = 0$ is the ridge penalty and $\alpha = 1$ is the lasso penalty. With intermediate values of α, there is a 'grouping' effect in which strongly correlated variables tend to enter or leave the model together (i.e., have their coefficients set to 0).

In the main text, we used elastic net regression to fit a logistic regression model of each domain type (conceptual, motor, medicine) as functions of our other coded (binary) variables. Following standard procedure, we used 10-fold cross-validation to find the optimum value of λ, i.e., the one that minimized cross-validation error.

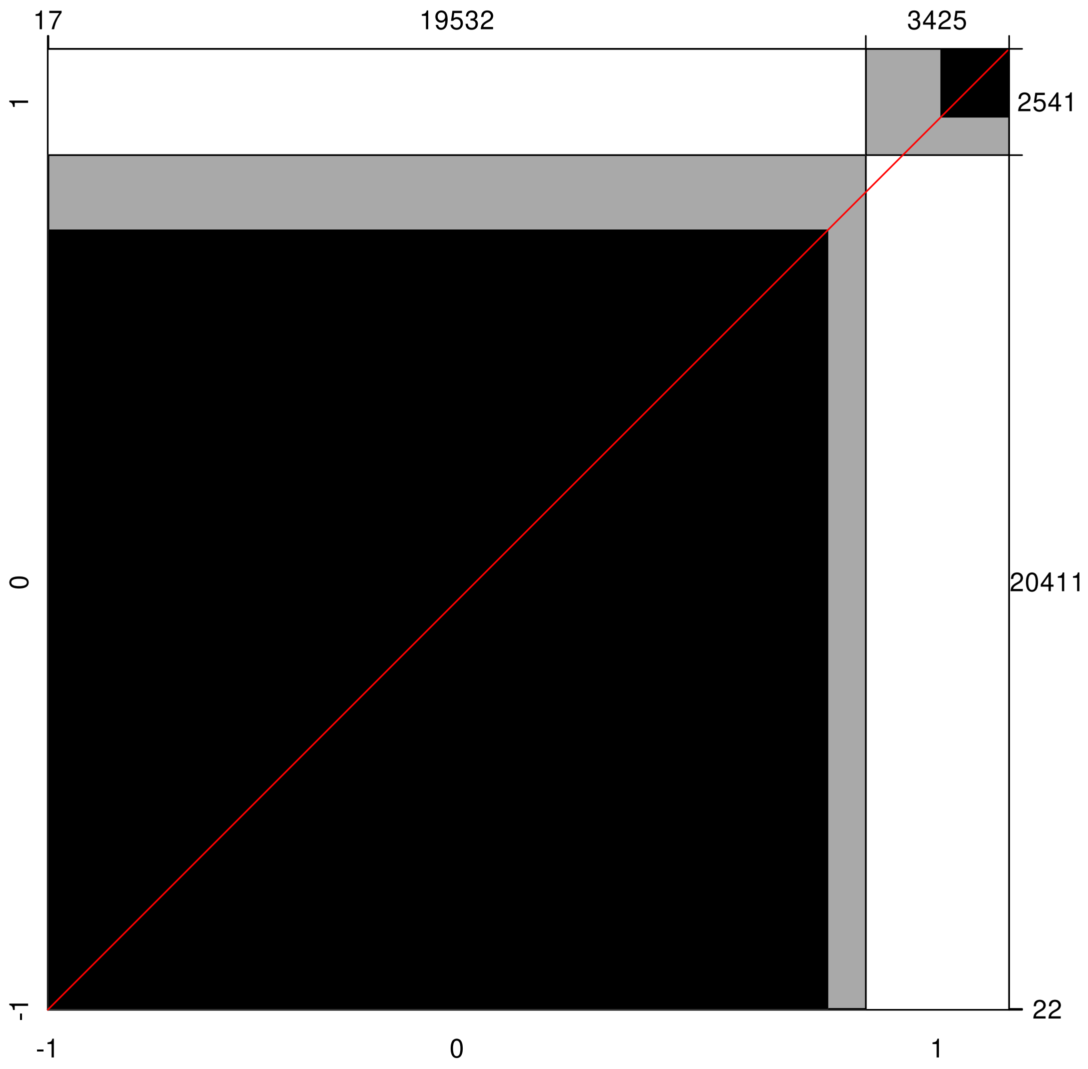

Figure S4: Agreement plot showing the proportions of agreement between the two independent coders (ADL and CH). The dark spaces represent the proportion of agreement for the presence and absence of all aggregated variables in the entire dataset, and the gray spaces represent the proportions of disagreement for presence and absence.

8 Filtering extremely sparse variables in our elasticnet regression

When we included all of the variables from our theoretical models in the elasticnet regression models, extremely sparse variables were prone to spurious and extremely large associations in a few cases. For example, the "mate fidelity" variable was only supported in one text record, and that text record also involved a specialist with medicinal knowledge (which was an outcome variable of one of our elasticnet regression models). Strictly speaking, presence of this variable was associated with medicinal domains, but with an extremely large regression coefficient and standard error. Although ridge regression is generally suitable for fitting models with many sparse predictor variables, exceptionally sparse predictors are nevertheless prone to large regression coefficients, with similarly large standard errors, that dominate and overshadow results among other (less sparse) predictor variables. Associations based on sparser predictors are not only theoretically uninteresting (because they are more likely to reflect noise than a signal in the data), but they create a practical problem when plotting them next to smaller regression coefficients with smaller standard errors.

We therefore chose to filter extremely sparse variables in our elasticnet regression. To do so, we filtered predictor variables that had evidence present in less than 5% of all text records in the dataset, which is a sum of about 26. Hence, because each variable consists of 1's and 0's, we included variables with a sum of at least 26 as predictors in the elasticnet regressions. These variables, along with those that were excluded due to insufficient evidence, are shown in figure S5.

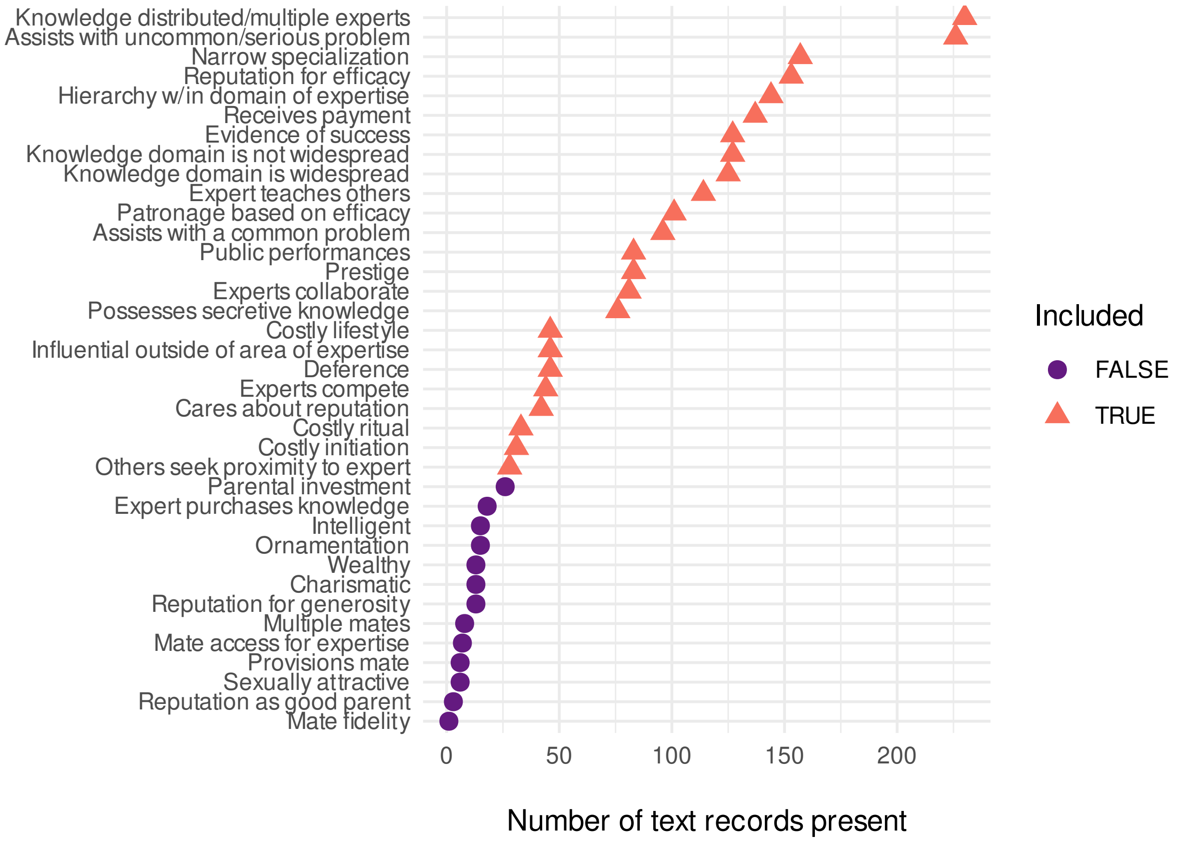

Figure S5: Dot chart showing the number of text records present for each variable in our theoretical models. The total number of text records present in each variable is shown along the x-axis, and colors and shapes correspond to whether or not this number was greater than or equal to 5% of the number of observations (rows) in our dataset. If they were, then they were included in our elasticnet regression.

9 Publication dates

The dates of publication for our ethnographic sources were mostly in the 20th century, though a few were in the 19th century and one was in the 18th century. We report key statistics about the publication dates and basic characteristics, but we show the distribution of publication dates here, both in the aggregate (figure S6) and in the 20th century only (figure S7).

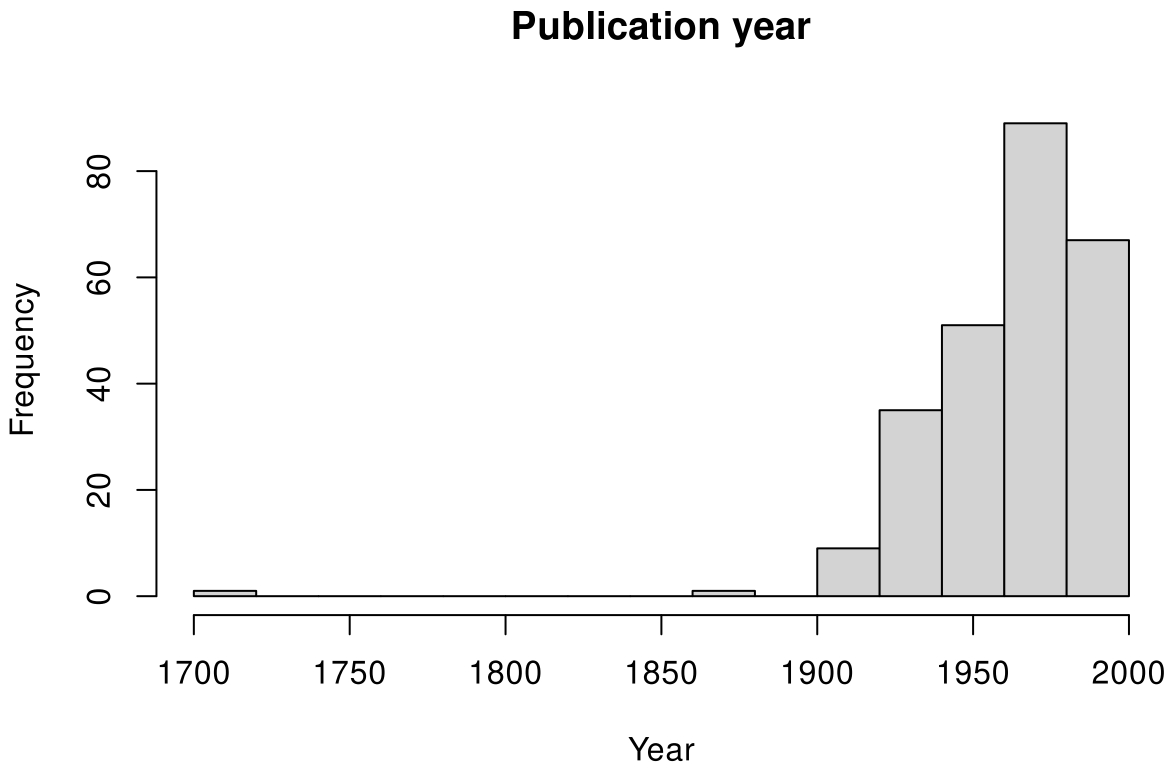

Figure S6: Histogram showing the number of text records by year in our entire dataset.

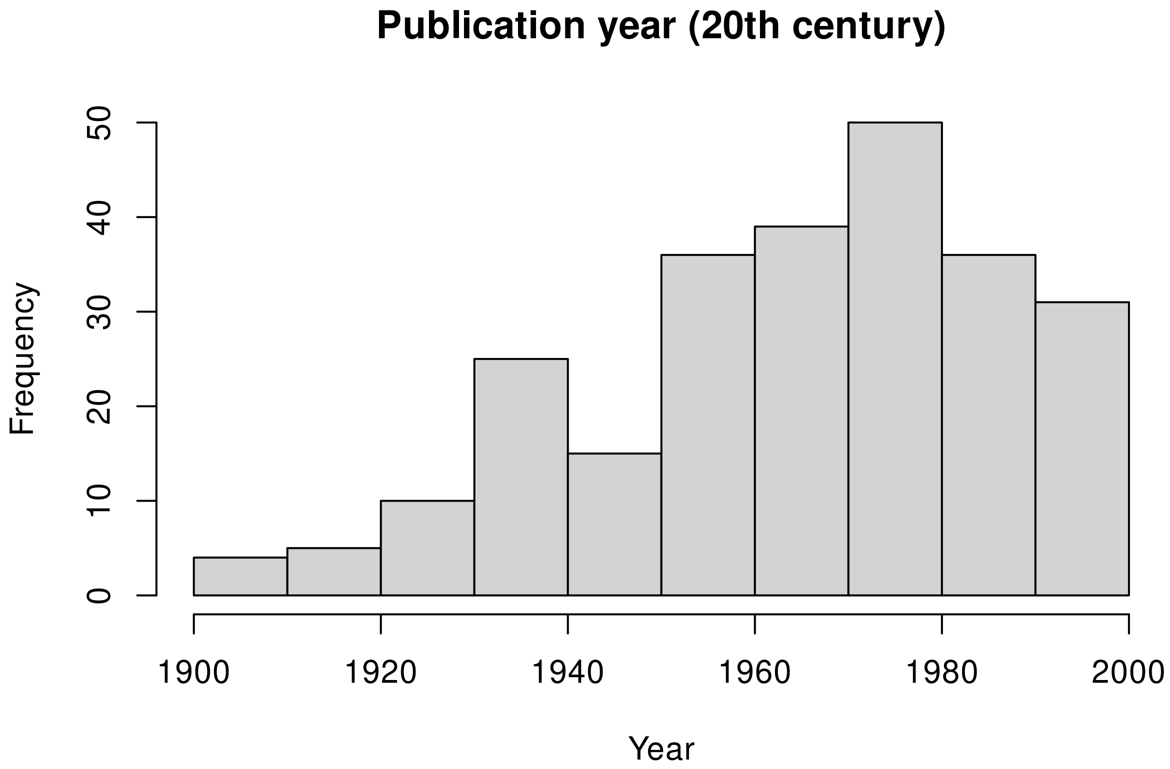

Figure S7: Histogram showing a closer look at the number of text records by year, among the publications that were from the 20th century only.

10 Analyzing the raw text in our dataset

To analyze our text record data for additional insights, we created a document term matrix of all of raw text in our dataset. This involves compiling all unique and lemmatized word types (e.g., "ran", "running", "runs" would all be collapsed into a single lemmatization of "run") and removes stop words, such as "a", "and", and "the". (Text records were generally short: after lemmatizing each word, text records ranged in unique word counts from 9 to 684, with a mean of 108, standard deviation of 87, and median of 80.) The resulting document term matrix is a large and sparse data matrix, where each row is a unique text record ID, each column is a unique and lemmatized word that occurred at least once in our dataset, and each element is the number of times that a lemmatized word occurred in a given text record. Our document term matrix dimensions were 547 by 9325.

We then compiled the level of support for each theoretical model in each text record. In a lasso regression, which is designed for penalizing exceptionally large numbers of predictor variables, we used the word frequencies of our 9325 lemmatized words in our document term matrix as predictors of model support for each theoretical model (our outcomes variables). In other words, this analysis asks what types of words tend to be associated with text records that are supportive of each model. In our results, many of the predictive words were noisy or culturally specific, but many appeared to correspond to model predictions, validating our models and their relevance to the coded texts. See figures S8 through S12 for results.

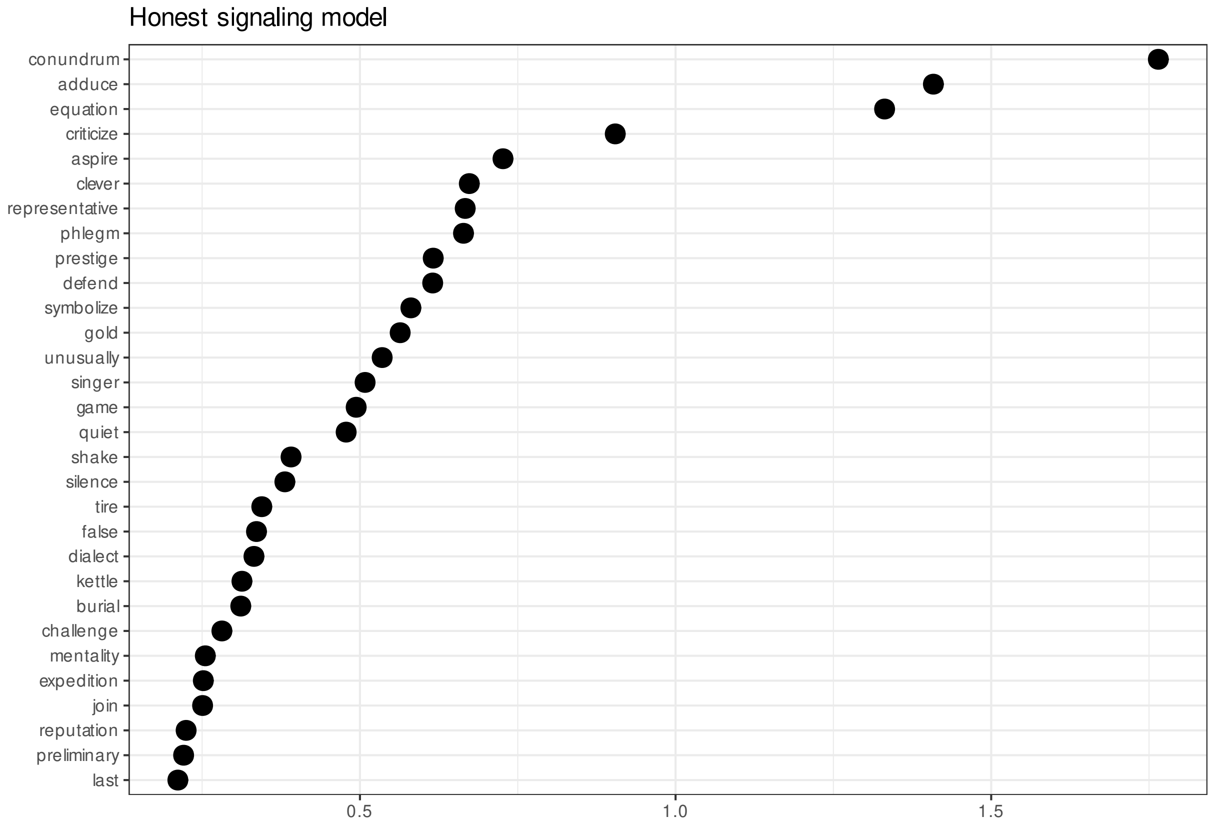

Figure S8: Lasso regression coefficients for lemmatized words in our document term matrix, which were predictive of support for the honest signaling model in each text record.

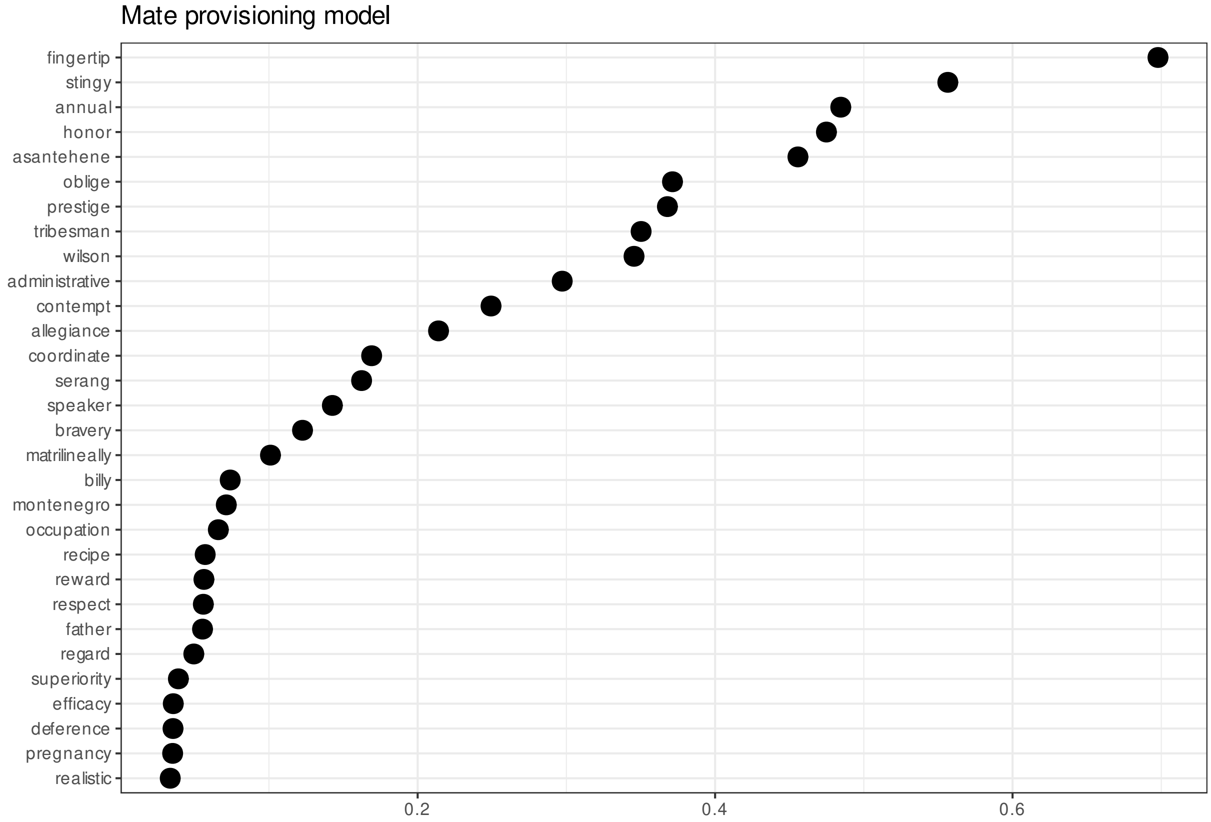

Figure S9: Lasso regression coefficients for lemmatized words in our document term matrix, which were predictive of support for the mate provisioning model in each text record.

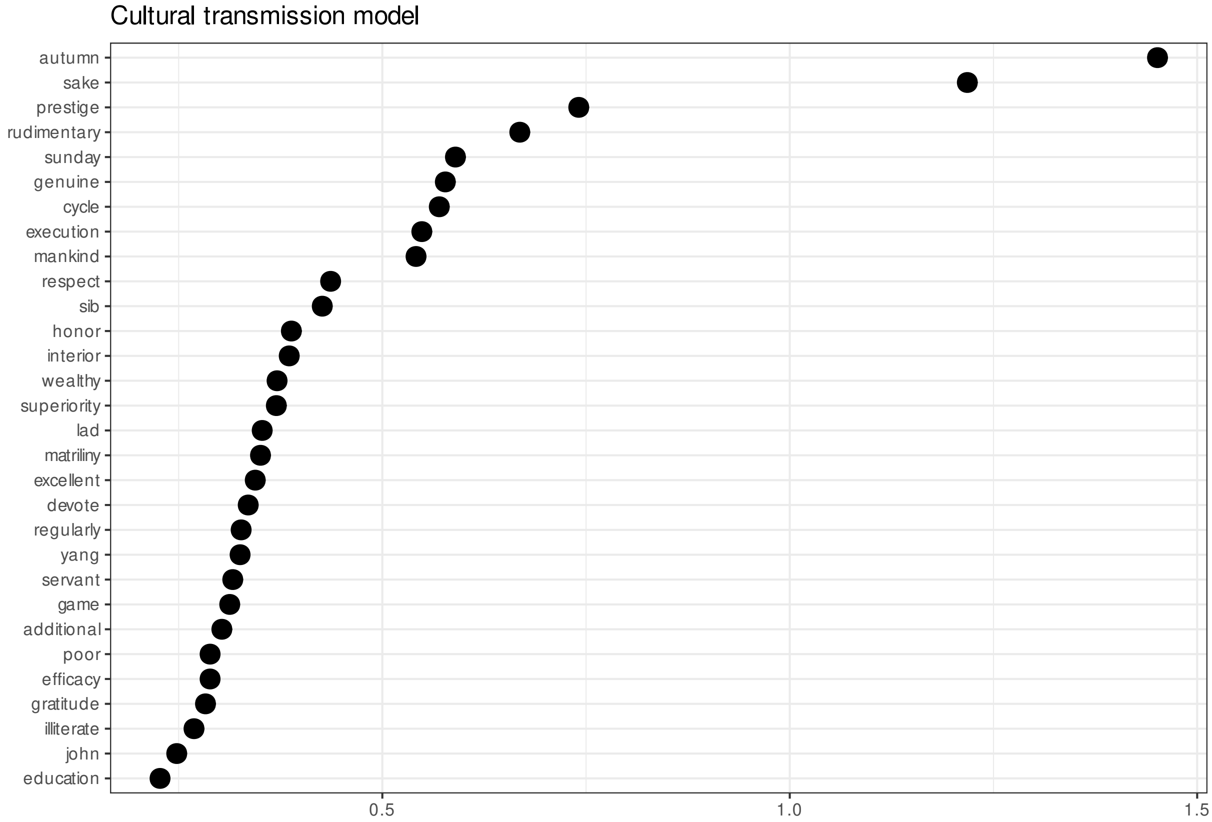

Figure S10: Lasso regression coefficients for lemmatized words in our document term matrix, which were predictive of support for the cultural transmission model in each text record.

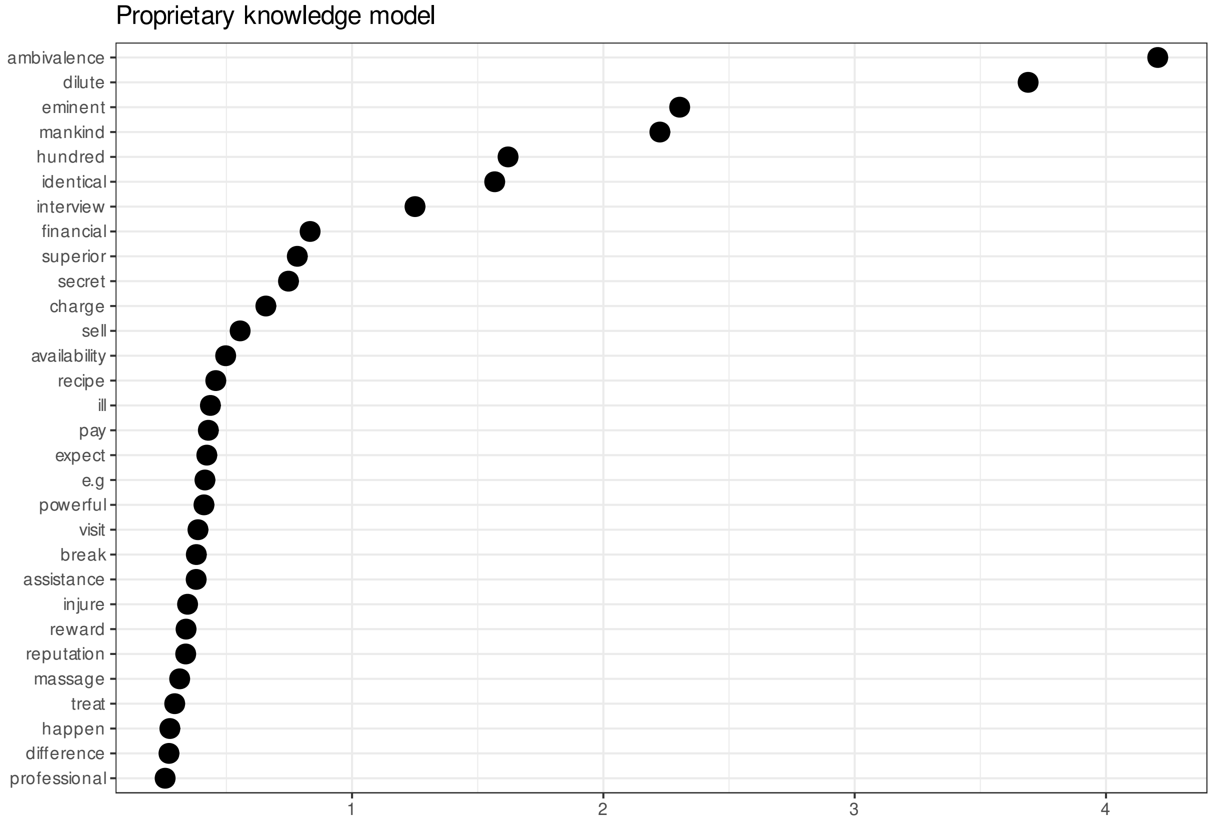

Figure S11: Lasso regression coefficients for lemmatized words in our document term matrix, which were predictive of support for the proprietary knowledge model in each text record.

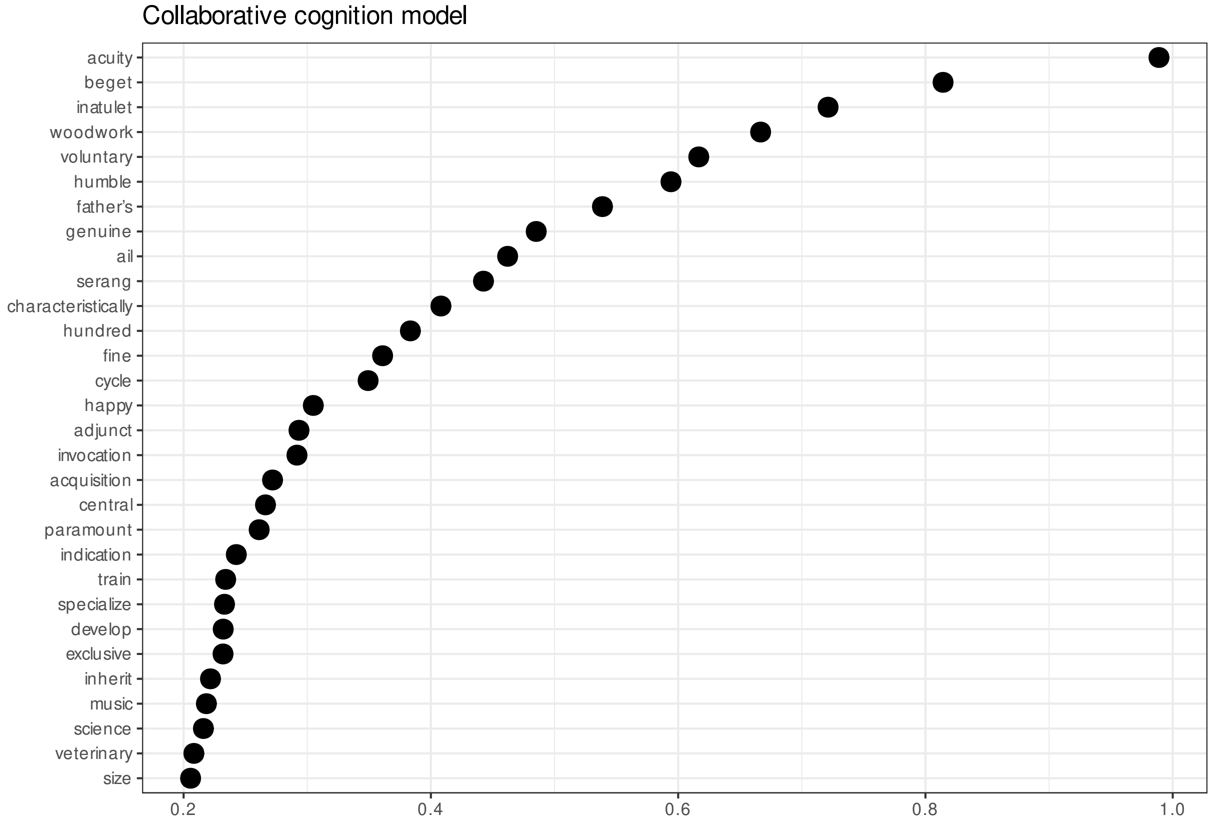

Figure S12: Lasso regression coefficients for lemmatized words in our document term matrix, which were predictive of support for the collaborative cognition model in each text record.

11 Raw count data for each variable

Although these values are shown in the main text as proportions of each variable, we include counts of support for each variable in our coding scheme in the table below for each variable in our theoretical models. See table S1.

Table S1: Number of text records coded as supporting each variable included in our coding scheme are shown here, and plotted in the main text.

Variable	Evidence
Knowledge distributed/multiple experts	230
Assists with uncommon/serious problem	226
Narrow specialization	157
Reputation for efficacy	153
Hierarchy w/in domain of expertise	144
Receives payment	137
Knowledge domain is not widespread	127
Evidence of success	127
Knowledge domain is widespread	125
Expert teaches others	114
Patronage based on efficacy	101
Assists with a common problem	96
Prestige	83
Public performances	83
Experts collaborate	81
Possesses secretive knowledge	76
Broad generalist	69
Deference	46
Influential outside of area of expertise	46
Costly lifestyle	46
Experts compete	44
Cares about reputation	42
Costly ritual	33
Costly initiation	31
Others seek proximity to expert	28
Private performances	26
Parental investment	26
Expert purchases knowledge	18
Anti-prestige/low status	17
Ornamentation	15
Intelligent	15
Reputation for generosity	13
Charismatic	13
Wealthy	13
Multiple mates	8
Mate access for expertise	7
Sexually attractive	6
Provisions mate	6
Reputation for selfishness	4
Mate infidelity	4

12 Shamans and lower class individuals in our spanning tree results

In the minimum spanning tree in main text, we found a variable cluster that did not resemble any of our theoretical models, which we referred to as "shamans and lower class". This label was determined by further investigation of the text records that were associated with support for the variables in the cluster (e.g., low status, anti-charasmatic individuals).

12.1 Examples of shamans and other spiritual figures in this cluster

Some of the specialists linked to this cluster were shamans, witchdoctors, and medicine-men who were disliked, distrusted, and/or avoided by members of the community. (We used "shamans" as shorthand in the main text, despite the nuance that this term and other religious/spiritual leadership terms actually entail.) Here are three examples of texts supporting this aspect of our interpretation.

Example #1:

> The love magic specialist makes many of the objects described in the preceding section: love dolls, tied and buried statuettes, flower insects, corpse oil, amulets and charms, Montra and Khaatha, and sacralized cosmetics. ... A majority of the specialists are males of lower status and income who are literate but not well educated. Most specialists know some Pali and Sanskrit words. They are consulted and paid by their customers, but they are not publicly and permanently respected. They are considered disgusting and are avoided by the majority of people in the society.

Example #2:

> One also finds, as in all other Buddhist lands, bonzesses living together in convents not far from the pagodas where they are forbidden to reside. Like the bonzes, they are supposed to remain continent during their stay in the bonzeries, and the penalty of death awaits those who give birth to children. For this reason, it is maintained, they are greatly skilled in the infamous art of abortion. Their morals have the reputation of being abominable.

Example #3:

> As they [the laymen] see it, the debtera begins his career as an ordinary farmer-priest, who performs in the mass in his natal parish. He is no different from his fellow priests, except for a weakness in his character which makes him unable to keep within the proscriptions of the priestly life. Eventually, it leads him to commit a serious moral offense (badal), after which he can no longer perform the mass. Most often, it is adultery that spoils the priest's identity. The theft of church tithes is another frequently mentioned act. Once discovered, he finds it impossible to continue his life in the parish. He is filled with shame; he is scorned by kinfolk and neighbors; and he loses his usufruct to church lands. But how is such a man to support himself once he has left the parish? Since his only resources now are his ability to read and write Amharic and Ge'ez (the language of the church) and his knowledge of the liturgy, he takes up a solitary wandering life as a debtera. His flawed nature gives him no peace, however, and he progresses from sin to sin until at last he seals his perdition by seeking the help of demons. At this point, he begins his career as a great magician, sorcerer, and healer.

12.2 Examples of low status occupations in this cluster

Others in this cluster were people in low status roles, but were not described as shamans or other spiritual figures. These were generally individuals who were either born into a lower strata of society, or were specializing in skill domains that were, themselves, considered demeaning or associated with a lower class. Here are three examples of texts supporting this aspect of our interpretation.

Example #1:

> Few herbalists claim to master this method fully. It is generally believed that the method is mostly used by those endogamous social groups who are descendants of slaves or who are outcasts. The baxaari, whom I have mentioned, is such a group, but in every Southern Somali village there are groups or individuals with a corresponding social position. Just like the baxaari such people may have an inferior social position and be feared for their magical skills, although they are often only talked about as dhiryaqaan, "the one who knows about herbs" ... We should note here, then, that there is some connection between the lower levels of society and knowledge of medical plants. This does not imply that the plants as such are seen as belonging to these groups, nor that using plants for curative purposes is defiling, only that certain socially peripheral groups are often believed to be particularly skilled in handling them.

Example #2:

> Wagoosh also taught Rogers's family how to market snakeroot by escorting his mother to town to exchange it for a "goodly supply of groceries." After that, he and his family "dug snake root every day, making several trips to town with it—so we always had a good supply of edibles." Snakeroot had become a significant source of income, and the Anishinaabeg learned how to get the best price for it. Some observers regarded collecting snakeroot and ginseng (Panax quinquefolius L.) as a demeaning way to make a living.

Example #3:

> The other important akombo performed for women with child is called the akombo swende. It is an act for the expulsion of evil and the transference of it into the body of a scape-goat. It is always performed by a male slave who must always disappear immediately afterwards.

13 Culture level support for each model

We analyzed model scores by three culture-level characteristics: Geographic region, subsistence strategy, and cultural complexity. We did not find any variation in levels of support (model score) by any of these predictors, suggesting consistent levels of evidence for each model cross-culturally. See figure S13 for model scores by geographic region, and figure S14 for model scores by subsistence strategy.

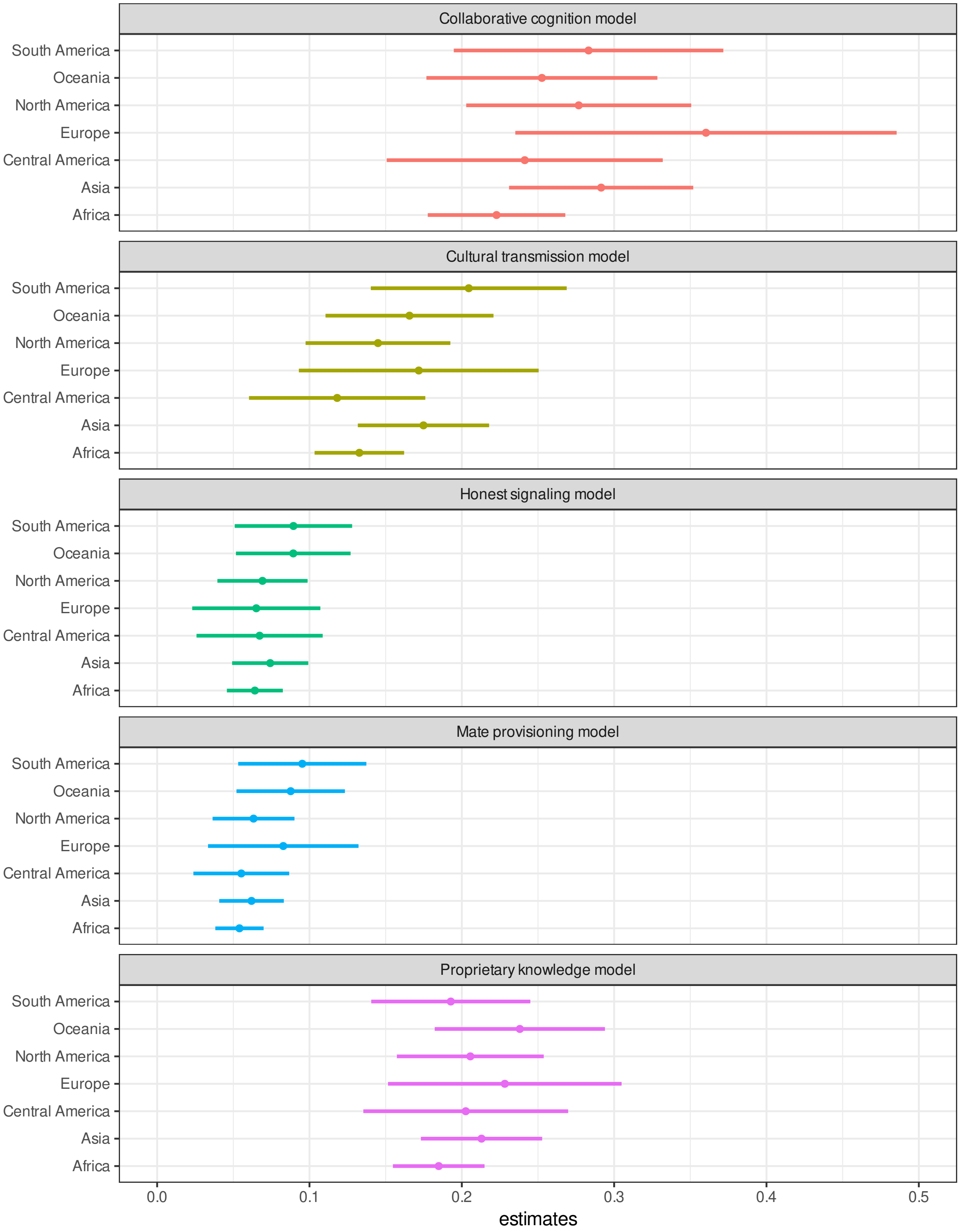

Figure S13: Culture level support for each model score by geographic region, computed as estimated margin means of the generalized linear mixed model estimates. Error bars are 95% confidence intervals.

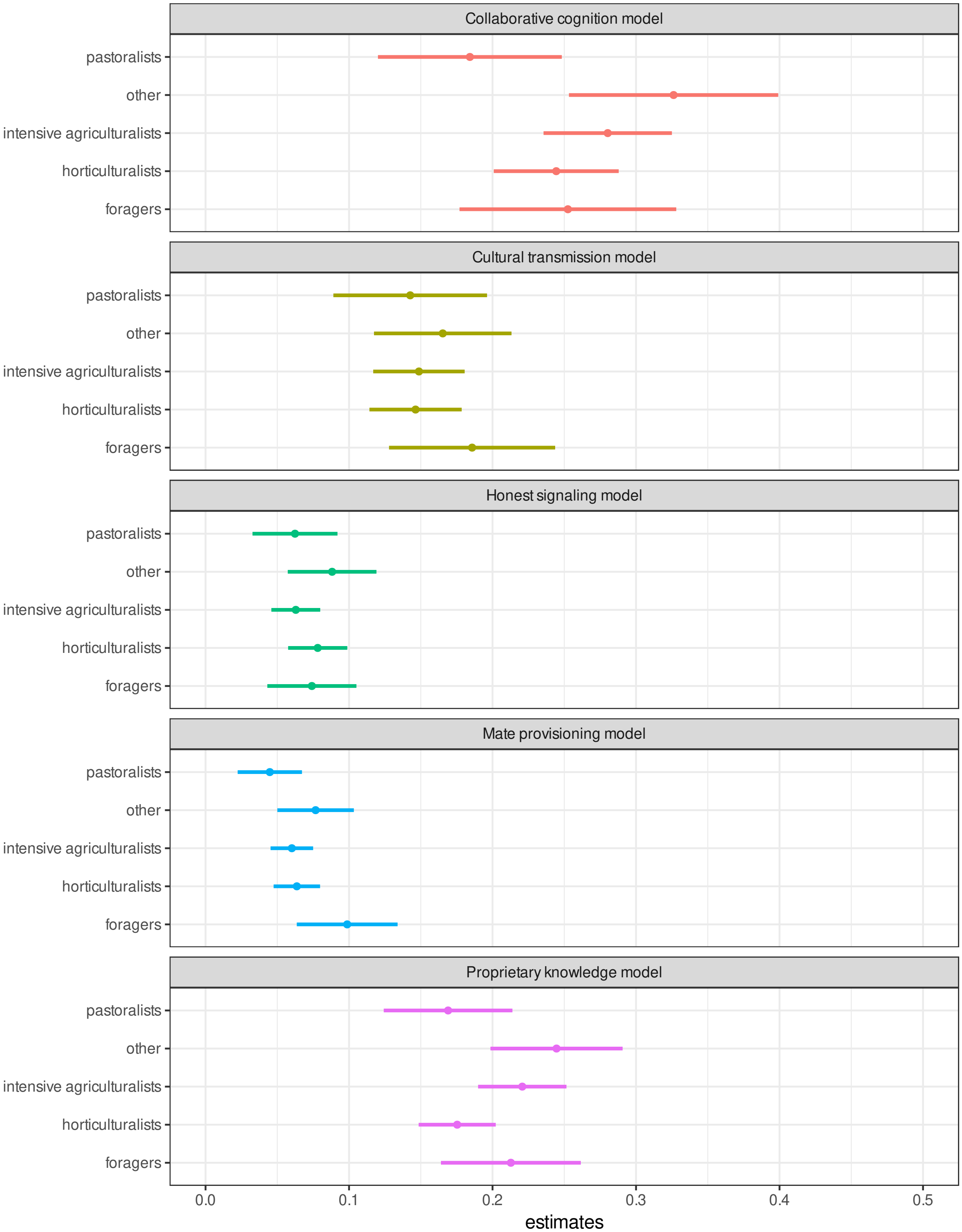

Figure S14: Culture level support for each model score by type of subsistence strategy, computed as estimated margin means of the generalized linear mixed model estimates. Error bars are 95% confidence intervals.

14 Analysis of sex differences

In this section, we analyze our data to address the possibility of sex-specific patterns. We report the relative levels of evidence of males and females in the main results, but, as we show in this section, sex-specific trends were less clear. At the text record level, and to broadly analyze how our coded variables might be associated with the presence of males and the presence of females, we used two separate ridge regression models. (See our explanation of this method in the elasticnet regression section above.) The outcome variable in one regression model was presence of males, and the outcome variable in the other regression model was presence of females. Males were generally more positively associated with prestige, public performances, and narrow specialization (figures S15 and S16), but we did not find any meaningful associations among females (figures S17 and S18).

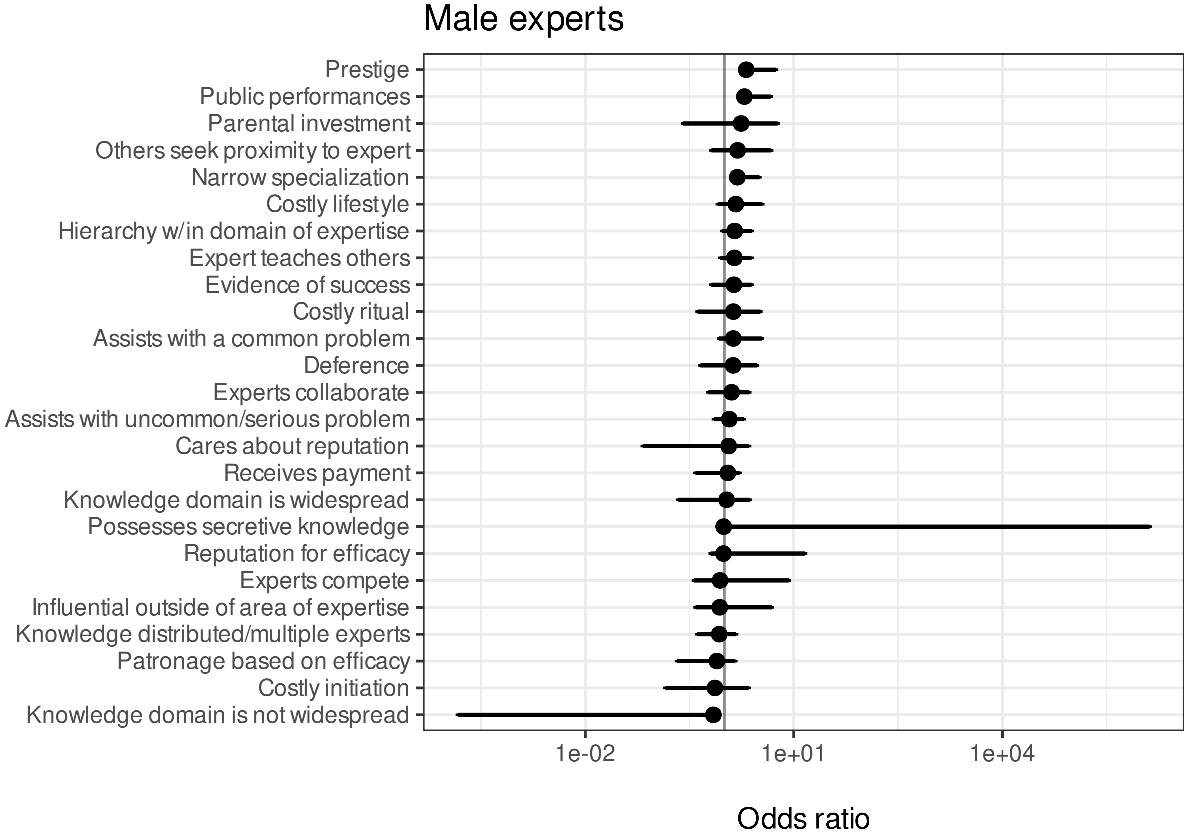

Figure S15: Ridge regression model of variables predicting evidence for males at the text record level. Regression coefficients are reported as odds ratios along the x-axis, and error bars are 95% confidence intervals. Note that the x-axis is log-scaled. This figure includes all variables that were included in the regression model for males, listed along the y-axis.

Replicating the heatmap from the main results (i.e., seriating rows and columns with PCA angle), annotating each text record to show the presence of males (figure S19) and the presence of females (figure S20) does not show any clear patterns. Females do appear to be slightly clustered into the rightmost section of the right cluster in figure S20, which is associated with uncommon and serious problems (e.g., medicine, illness). It is difficult to make strong inferences from the high female presence here, however, because these particular text records substantially overlap with the left cluster.

To further investigate sex-specific patterns, we recreated the heatmap using sex-specific data matrices (i.e., a "male" data matrix of *only* text records with males present, and a "female" data matrix of *only* text

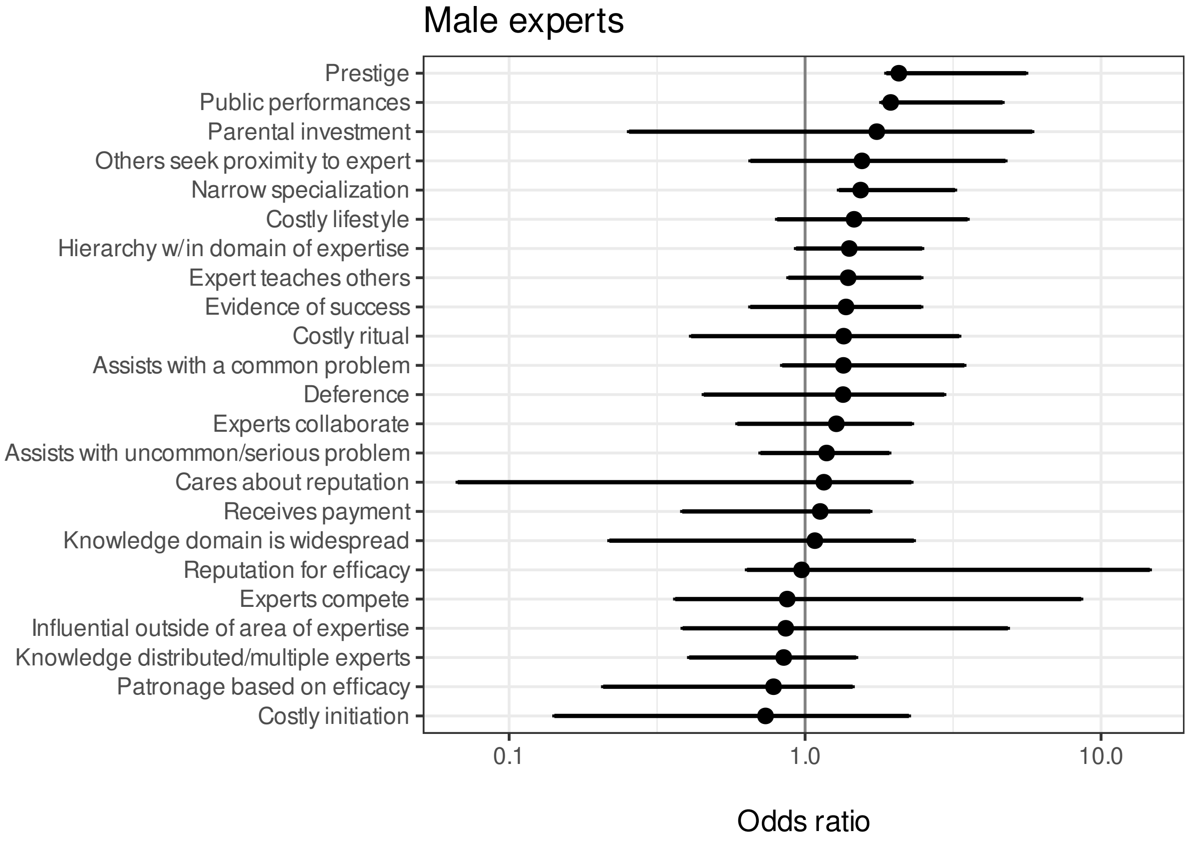

Figure S16: Ridge regression model of variables predicting evidence for males at the text record level. Regression coefficients are reported as odds ratios along the x-axis, and error bars are 95% confidence intervals. Note that the x-axis is log-scaled. This figure replicates the previous figure, but removes variables with extremely large CI's to make our results more interpretable.

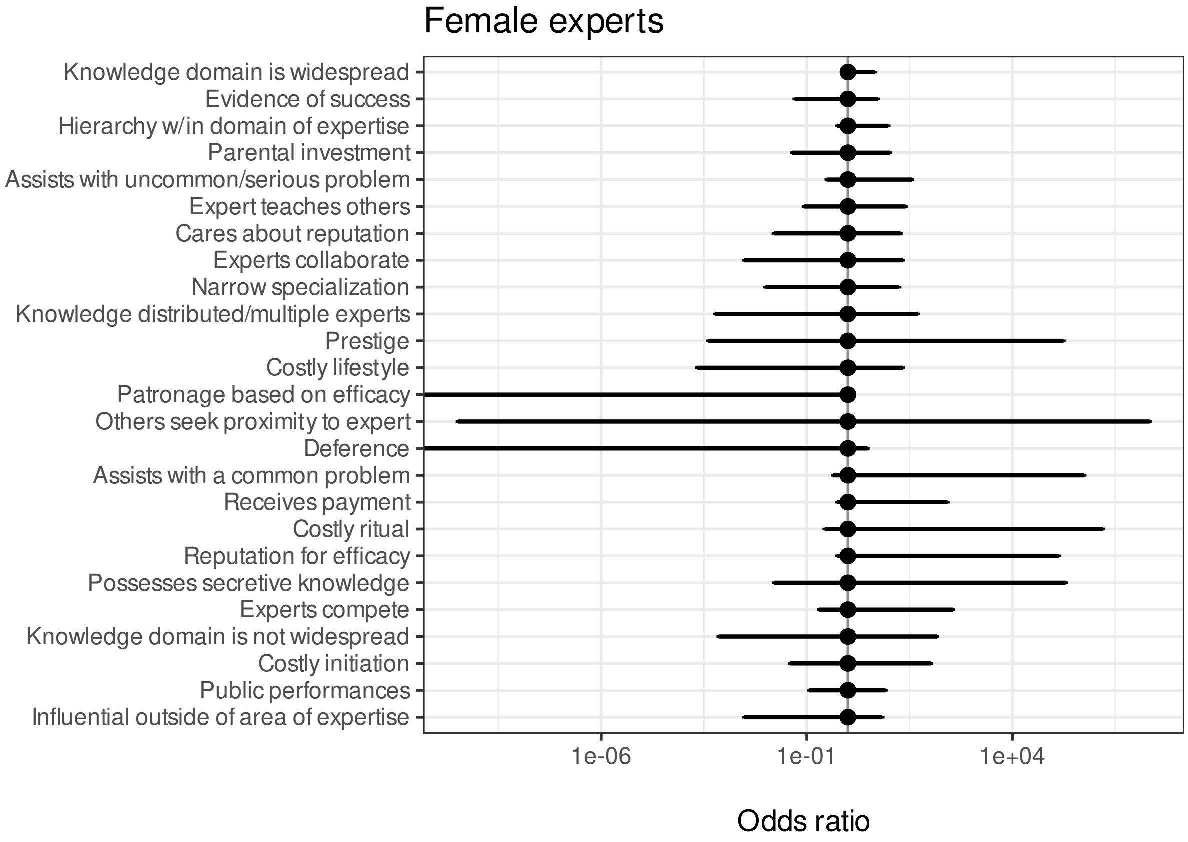

Figure S17: Ridge regression model of variables predicting evidence for females at the text record level. Regression coefficients are reported as odds ratios along the x-axis, and error bars are 95% confidence intervals. Note that the x-axis is log-scaled. This figure includes all variables that were included in the regression model for females, listed along the y-axis.

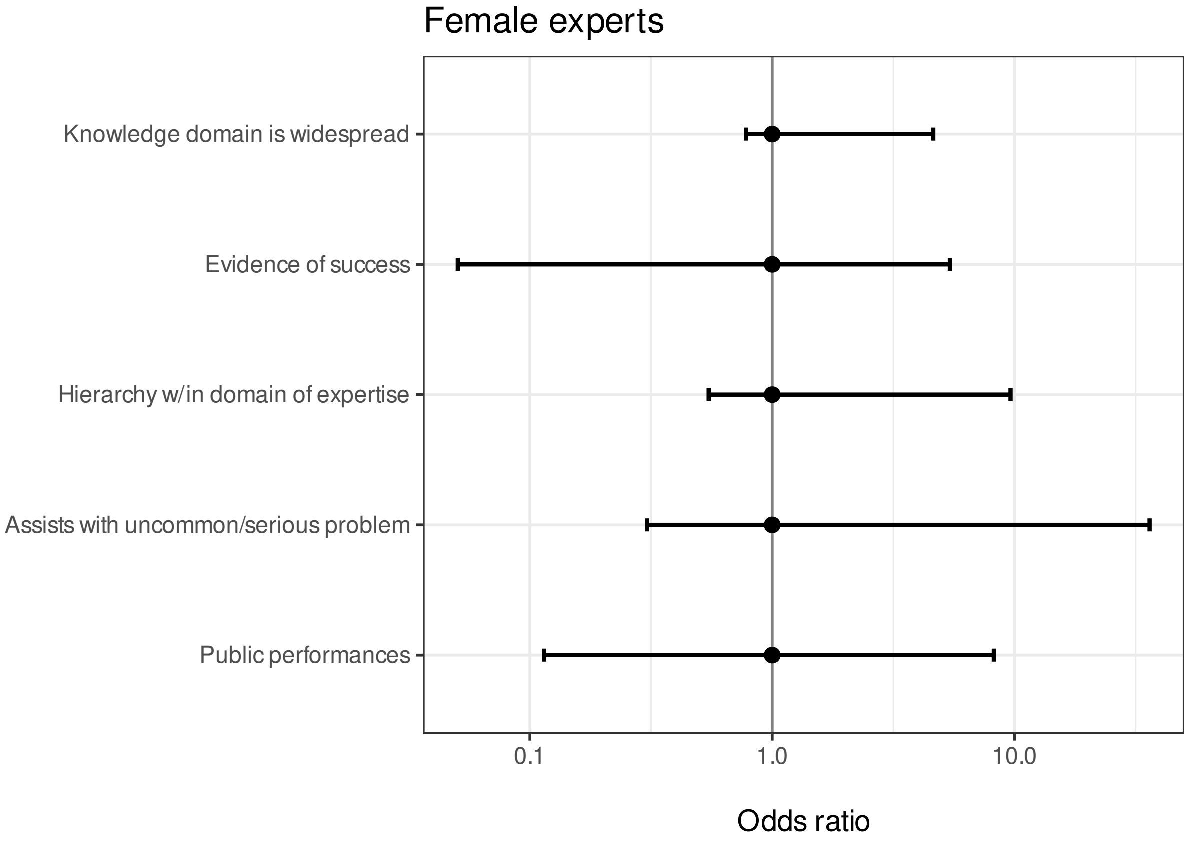

Figure S18: Ridge regression model of variables predicting evidence for females at the text record level. Regression coefficients are reported as odds ratios along the x-axis, and error bars are 95% confidence intervals. Note that the x-axis is log-scaled. This figure replicates the previous figure, but removes variables with extremely large CI's to make our results more interpretable.

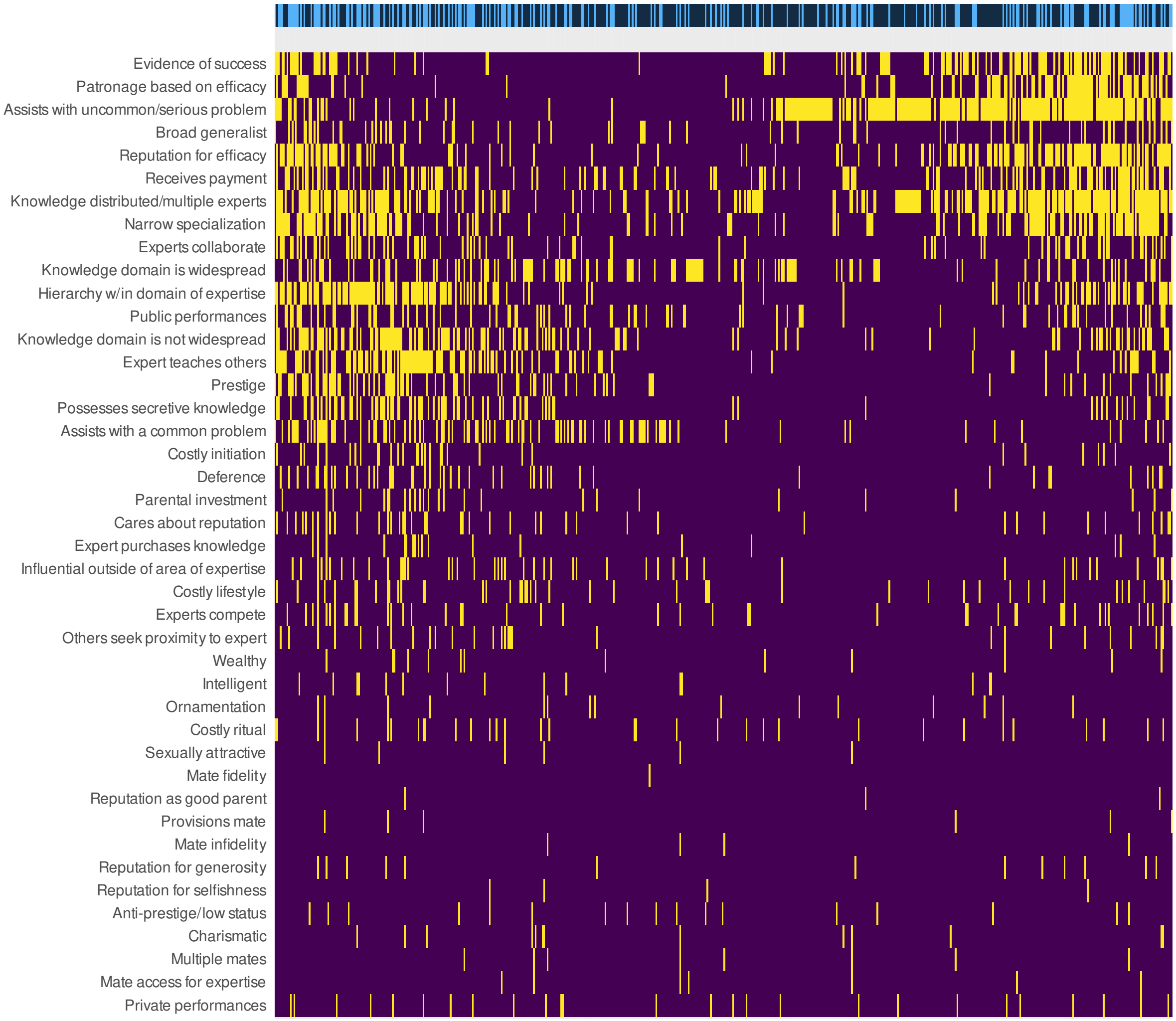

Figure S19: Heatmap visualizing the coded dataset based on presence (light cells) vs. absence (dark cells) of evidence for each variable in each text record. For readability, the dataset shown here is transposed, i.e., each row represents a variable and each column represents a single text record. Rows and columns are ordered using the PCA angle seriation method, as shown in the main text. The annotated uppermost row indicates which text records have male experts present (light blue cells).

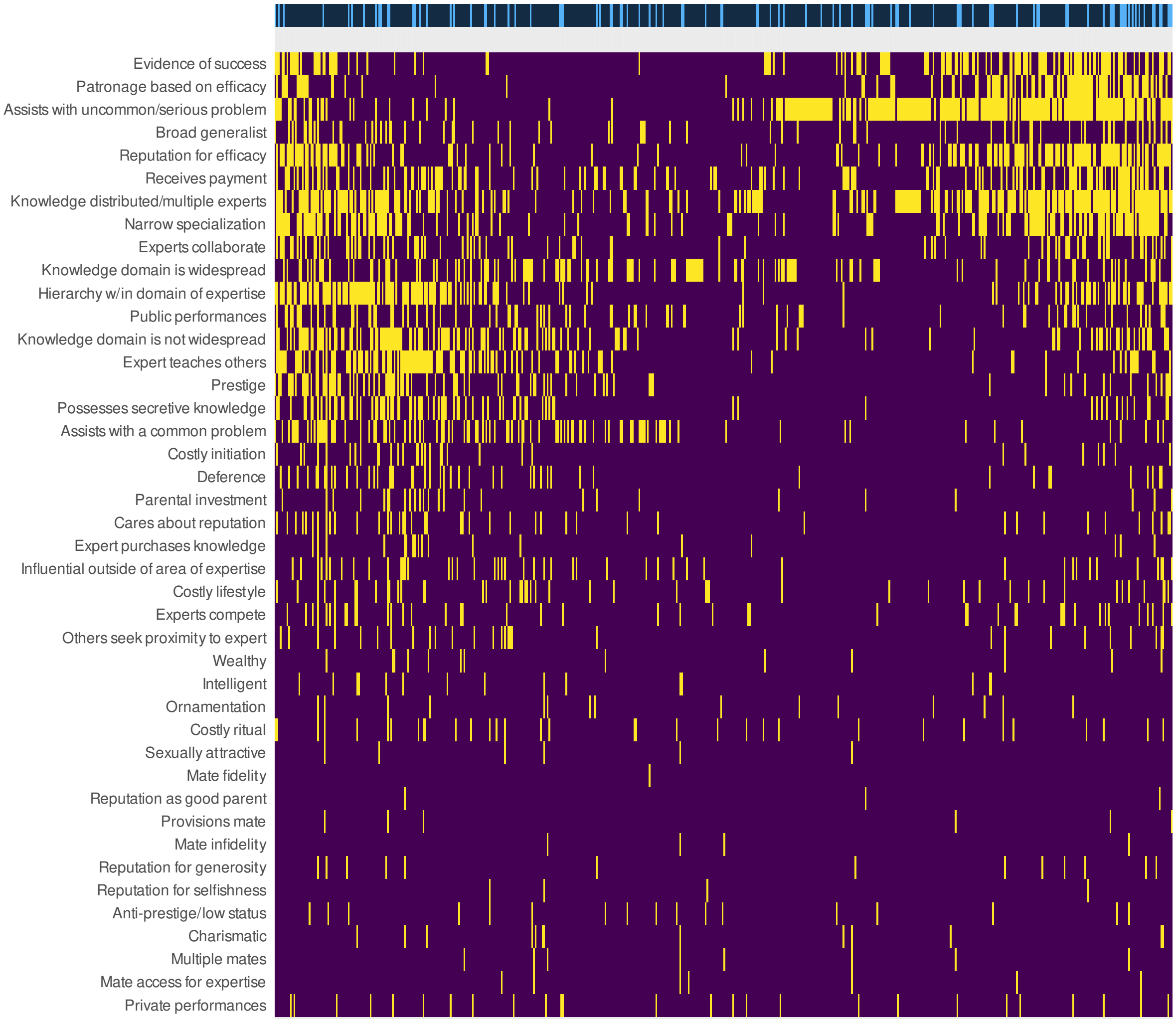

Figure S20: Heatmap visualizing the coded dataset based on presence (light cells) vs. absence (dark cells) of evidence for each variable in each text record. For readability, the dataset shown here is transposed, i.e., each row represents a variable and each column represents a single text record. Rows and columns are ordered using the PCA angle seriation method, as shown in the main text. The annotated uppermost row indicates which text records have female experts present (light blue cells).

records with females present), we see trends in each that are similar to the heatmap based on our aggregated dataset (in the main text). See figures S21 and S22.[1] These trends, especially in the female data matrix, are coarser-grained compared to the original heatmap from the main results. This is because the number of female-present text records ($N = 102$) was lower than the number of male-present text records ($N = 201$) and both were far lower than the number of text records in the entire data matrix from our original heatmap ($N = 547$).

Another key exploratory analysis from our results was the minimum spanning tree (MST). We therefore replicated this analysis using the female data matrix (figure S23) and the male data matrix (figure S24). For females, we saw little-to-no interpretable clustering among the variables, although it is worth noting that assistance with uncommon and serious problems appears to be important to female experts in general. (This is consistent with the suggestive result in the heatmap in figure S22 that females were generally clustered into the uncommon and serious problems cluster, and the observation that most females – about 55% of text records with females present – are associated with medicinal domains). For males, hierarchies within a given domain of expertise, prestige, and teaching were important variables. This is generally consistent with the key results of this section from figure S16, i.e., that males are generally more associated with prestige. Interestingly, the uppermost cluster of figure S24 suggests some coherent male support for variables in the mate provisioning model, but the scarcity of evidence for these variables overall makes this a more speculative findings.

[1]Notice that the clusters are "flipped" to the bottom of these heatmaps, compared to the heatmap in our main results. This is a result of the PCA angle seriation method, and the position of these clusters on the heatmap is arbitrary. The key result to notice is that *which variables* that are clustering together remains largely in tact.

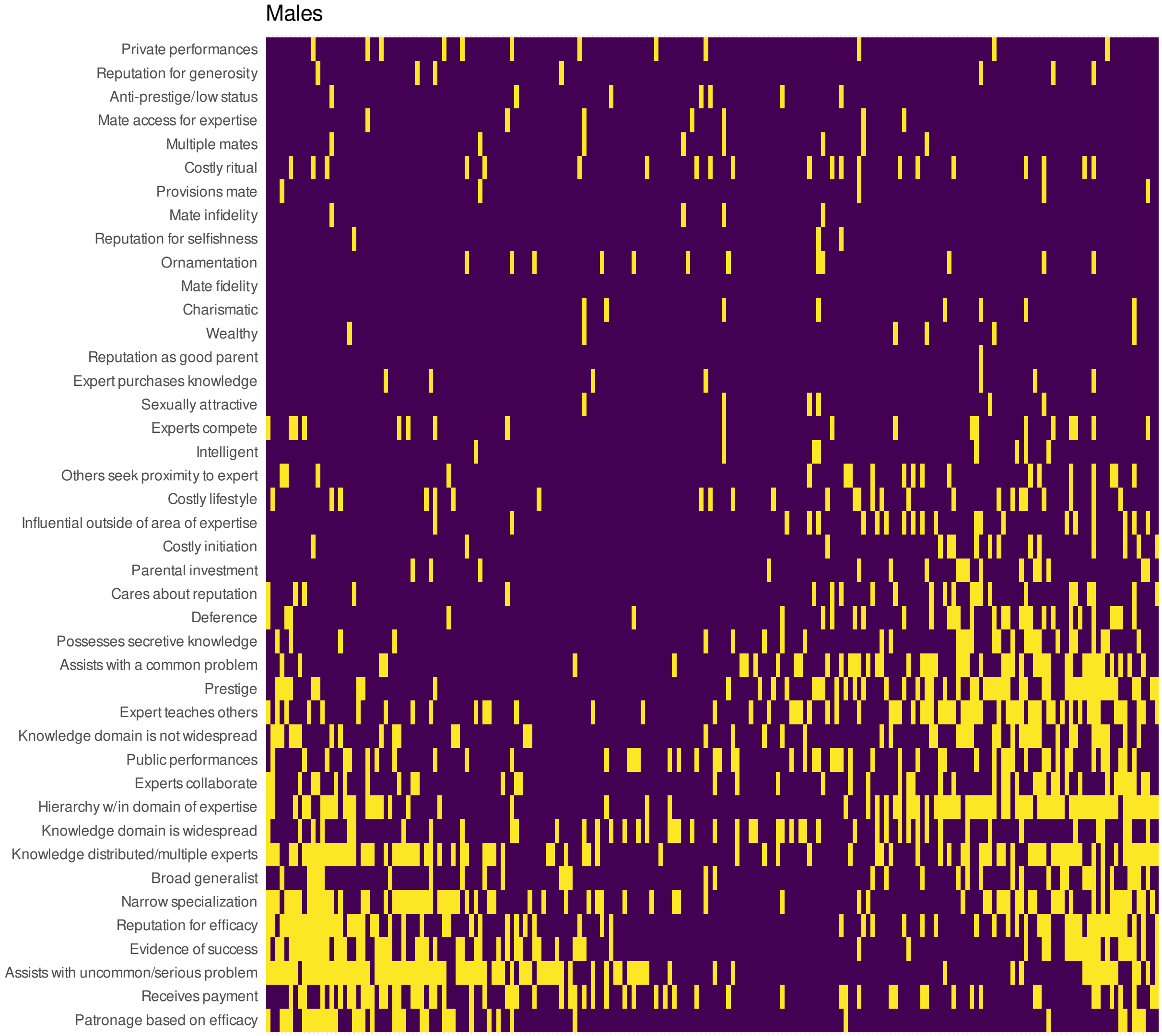

Figure S21: Heatmap visualizing the coded dataset among only text records with male experts present, based on presence (light cells) vs. absence (dark cells) of evidence for each variable in each text record. For readability, the dataset shown here is transposed, i.e., each row represents a variable and each column represents a single text record. Rows and columns are ordered using the PCA angle seriation method, as shown in the main text.

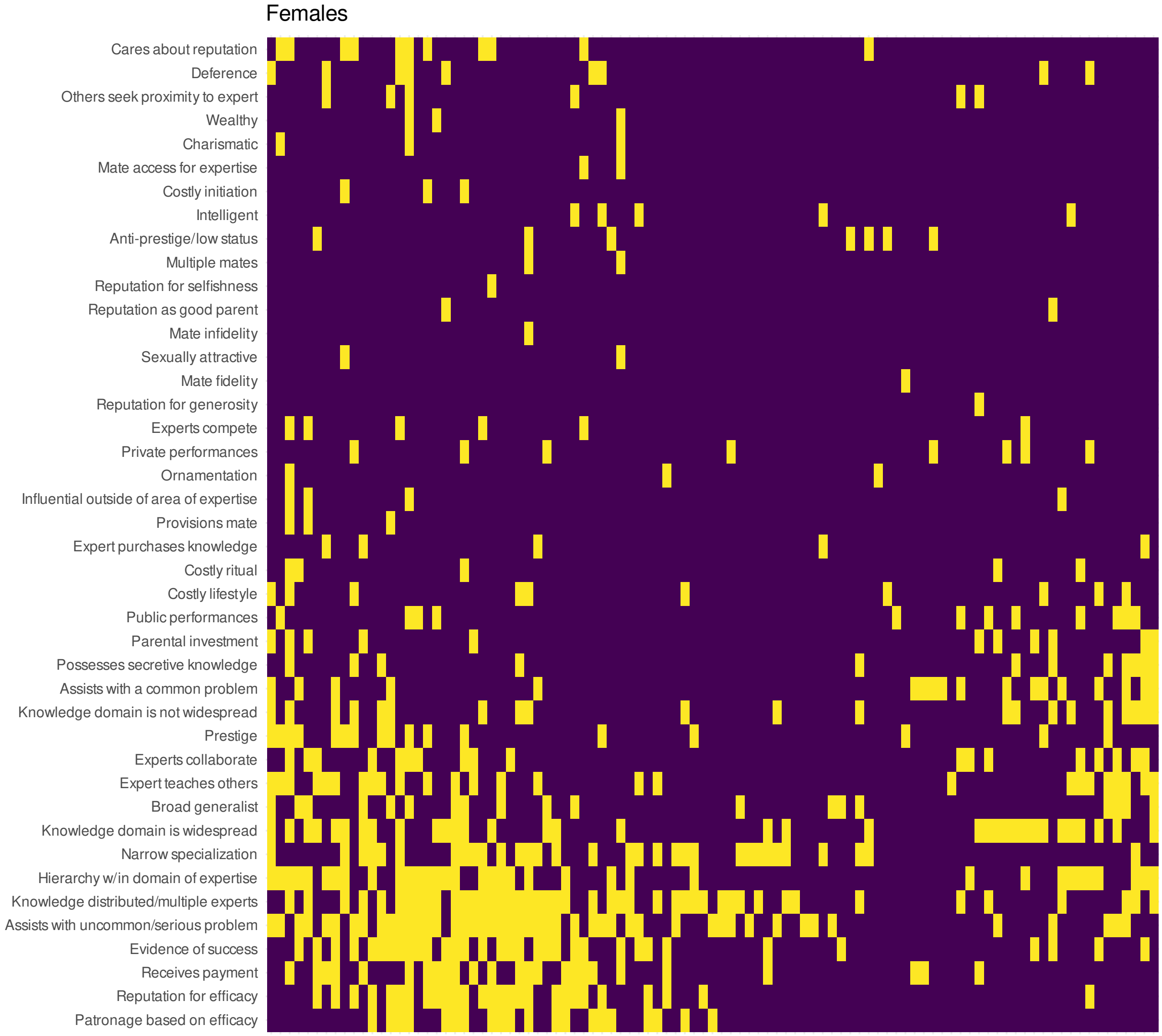

Figure S22: Heatmap visualizing the coded dataset among only text records with male experts present, based on presence (light cells) vs. absence (dark cells) of evidence for each variable in each text record. For readability, the dataset shown here is transposed, i.e., each row represents a variable and each column represents a single text record. Rows and columns are ordered using the PCA angle seriation method, as shown in the main text.

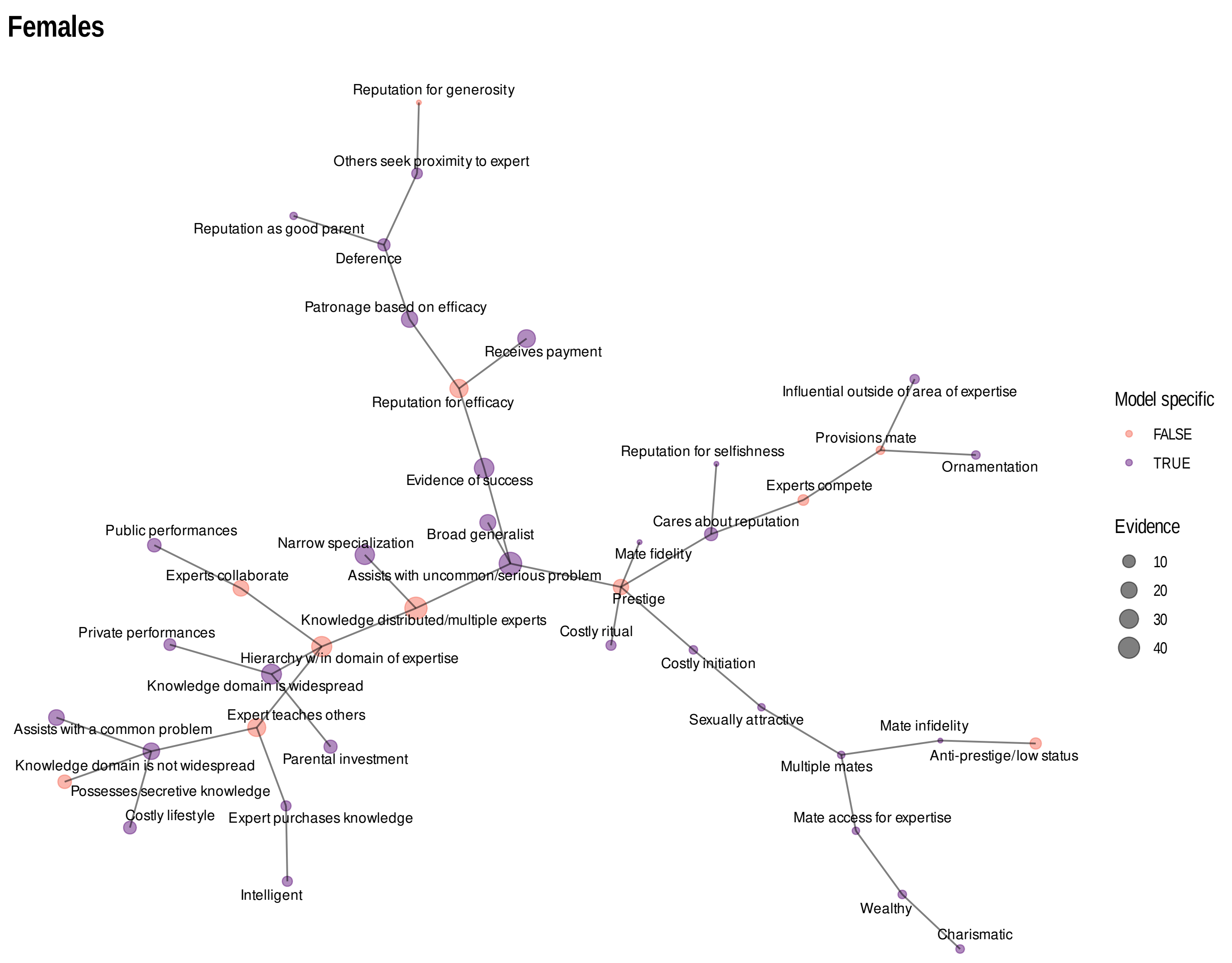

Figure S23: Minimum spanning tree of the variable binary distance matrix for text records with females present. Vertices represent variables, vertex sizes correspond to levels of text record support for each variable, and vertex colors to whether or not the variable is model specific vs. model generic.

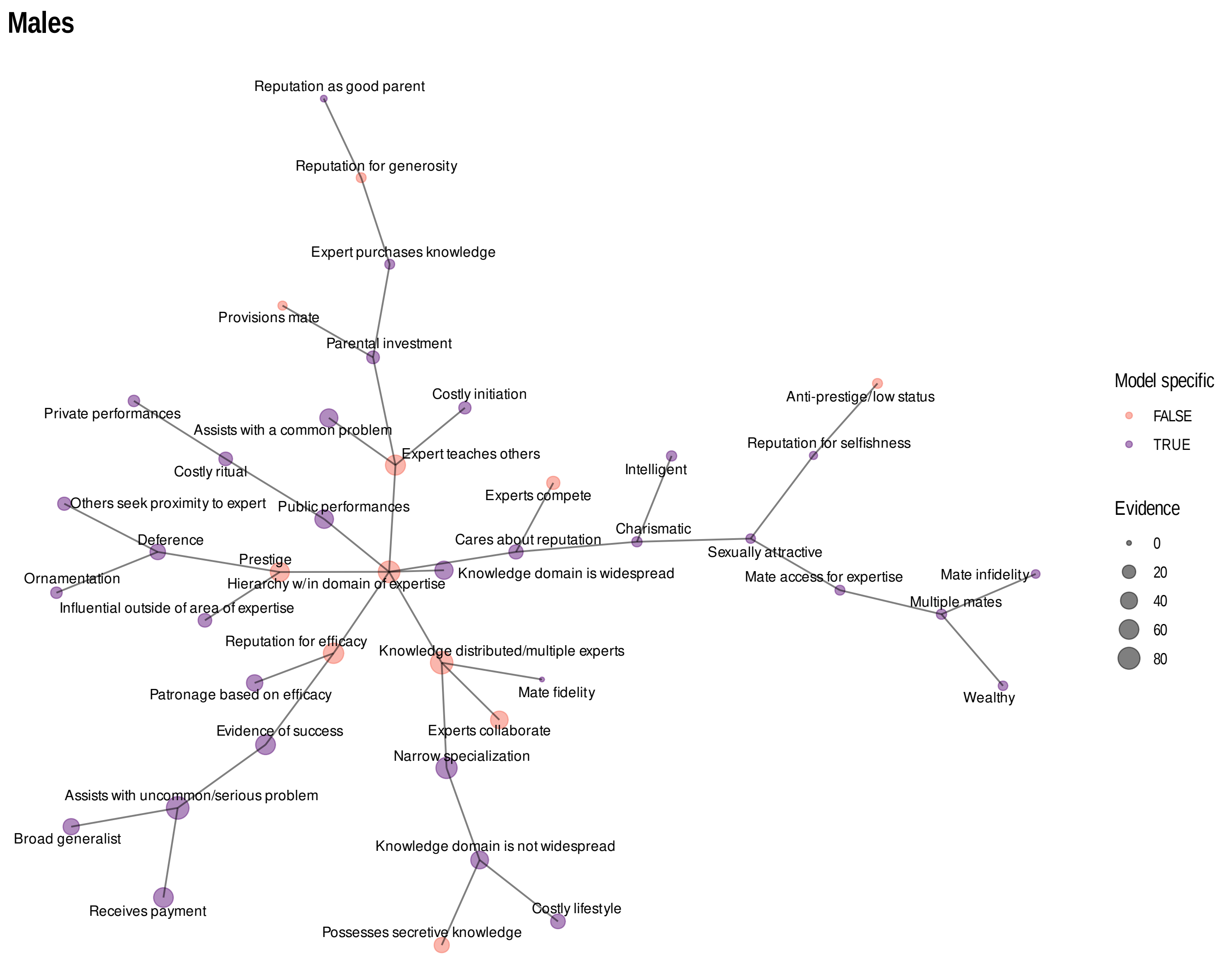

Figure S24: Minimum spanning tree of the variable binary distance matrix for text records with males present. Vertices represent variables, vertex sizes correspond to levels of text record support for each variable, and vertex colors to whether or not the variable is model specific vs. model generic.

Lastly, it is worth addressing the possibility that sex-specific roles co-occurred in some text records where males and females were *both* present. (For example, in a few cases, males had specialized knowledge for healing and divination whereas females had specialized knowledge about midwifery and childbirthing.) This co-occurrence of sex-specific roles and males and females in a single text record occurred in 12 text records (about 2% of our dataset). These text records were not sufficiently descriptive to infer sex-specific trends that were relevant to our theoretical models. Further, recreating the heatmap from our main results, and annotating these 12 text records, did not suggest that they were generally associated with one of our data clusters. See figure S25.

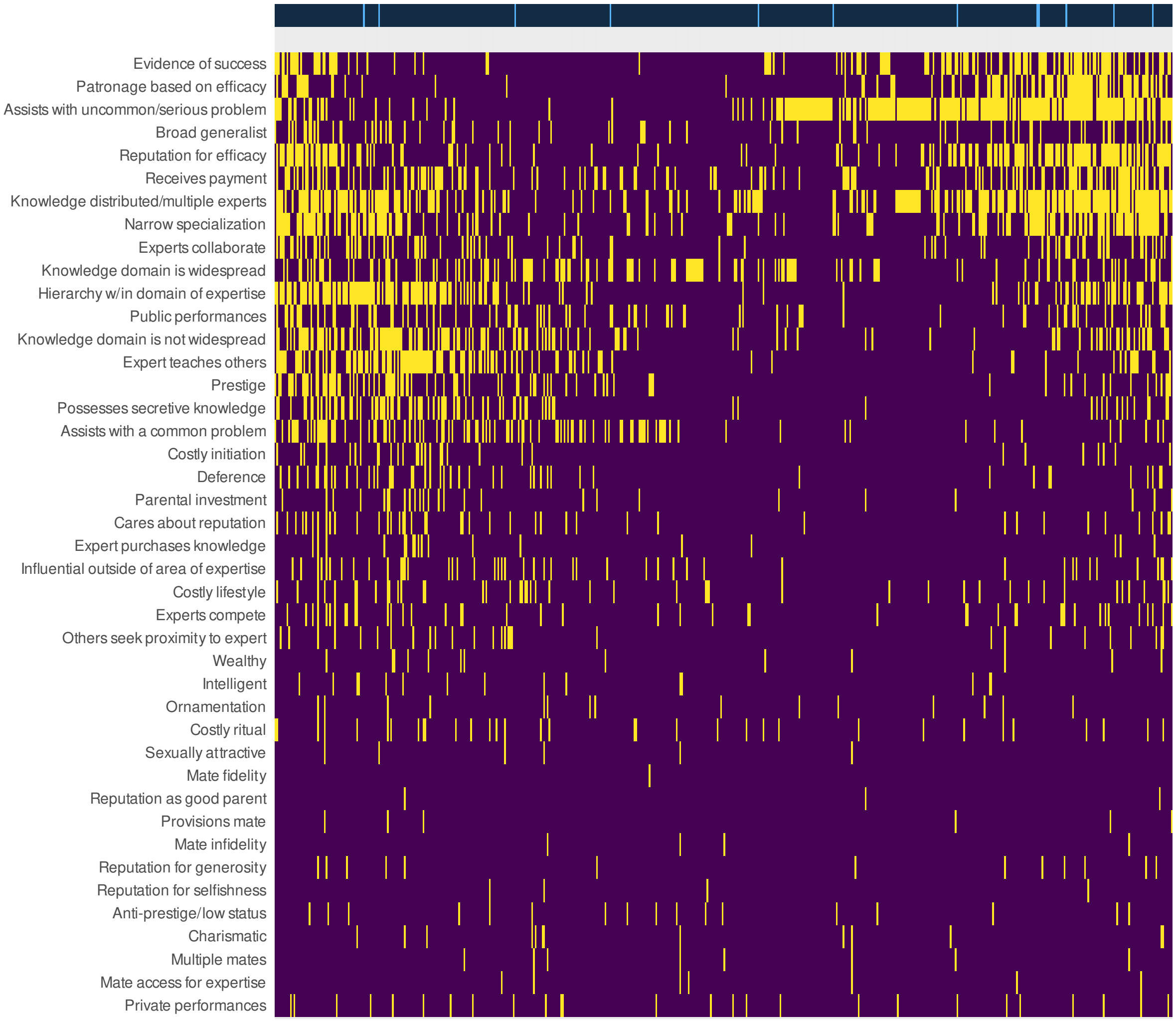

Figure S25: Heatmap visualizing the coded dataset based on presence (light cells) vs. absence (dark cells) of evidence for each variable in each text record. For readability, the dataset shown here is transposed, i.e., each row represents a variable and each column represents a single text record. Rows and columns are ordered using the PCA angle seriation method, as shown in the main text. The annotated uppermost row indicates which text records have both male and female experts present, with some kind of sex-specific specialization involved (light blue cells).
